# Design, Synthesis, Structure–Activity Relationships, and Preliminary Anticancer Properties of Menthol-Modified Coumarin Esters and 3,4-Dihydrocoumarin Derivatives

**DOI:** 10.1021/acsomega.5c01784

**Published:** 2025-10-01

**Authors:** Katarzyna Szwaczko, Paulina Strzyga-Łach, Marta Struga, Ewelina Kiernozek-Kalińska, Krzysztof Szafrański, Adrianna Skiba, Anita Płazińska, Krystyna Skalicka-Woźniak, Anna Bielenica

**Affiliations:** † Department of Organic Chemistry and Crystallochemistry, Institute of Chemical Sciences, Faculty of Chemistry, 49554 Marie Curie-Skłodowska University in Lublin, 20-614 Lublin, Poland; ‡ Chair and Department of Biochemistry, 37803 Medical University of Warsaw, 02-097 Warsaw, Poland; § Department of Immunology, Faculty of Biology, 49605 University of Warsaw, 02-096 Warsaw, Poland; ∥ Department of Organic Chemistry, Faculty of Pharmacy, 195046 Medical University of Gdańsk, 80-416 Gdańsk, Poland; ⊥ Department of Natural Products Chemistry, Faculty of Pharmacy, 427070 Medical University of Lublin, 20-093 Lublin, Poland; # Department of Biopharmacy, Medical University of Lublin, ul. Chodzki 4a, 20-093 Lublin, Poland

## Abstract

Menthol-modified coumarin esters (**1a**–**e**) and 3-phosphorylated coumarins (**2a**, **2b**, **3**) were synthesized. The Michael addition of P­(O)H groups to the coumarin skeleton offered access to 3,4-dihydrocoumarin derivatives (**4a**–**4d**, **5a**–**5b**). The addition reaction proceeded with high yields (89–98%) in a short time under mild temperature conditions and environmentally friendly solvents such as CH_3_CN or water. The resulting compounds (**1a**–**1f**, **2a**, **2b**, **3**, **4a**–**4d**, **5a**–**5b**) were subjected to *in vitro* cytotoxicity evaluation against human cancer cell lines: colorectal (SW480, SW620), prostate (PC3), breast (MDA-MB-231), and human keratinocytes (HaCaT) by MTT assay. Doxorubicin and cisplatin were used as reference compounds. Based on the findings, the three most promising compounds (**2b**, **4a**, **4b**) were selected for further biological studies, which included evaluation of their ability to induce apoptosis, inhibit IL-6 secretion, and antiproliferative activity. Compounds such as **4a** and **4b** were tested as diastereomeric mixtures due to the limited possibility of separating them into individual isomers at this stage. Nevertheless, promising activity and selectivity against selected cancer cell lines were observed. The *in vivo* toxicological evaluation performed on zebrafish larvae indicated that coumarin **2b** did not present toxic effects. In addition, the prediction of physicochemical properties, ADME, and pharmacokinetic profiles was performed by *in silico* methods. The results indicate the significant potential of the selected compounds as candidates for further research toward the design of new bioactive compounds.

## Graphical abstract

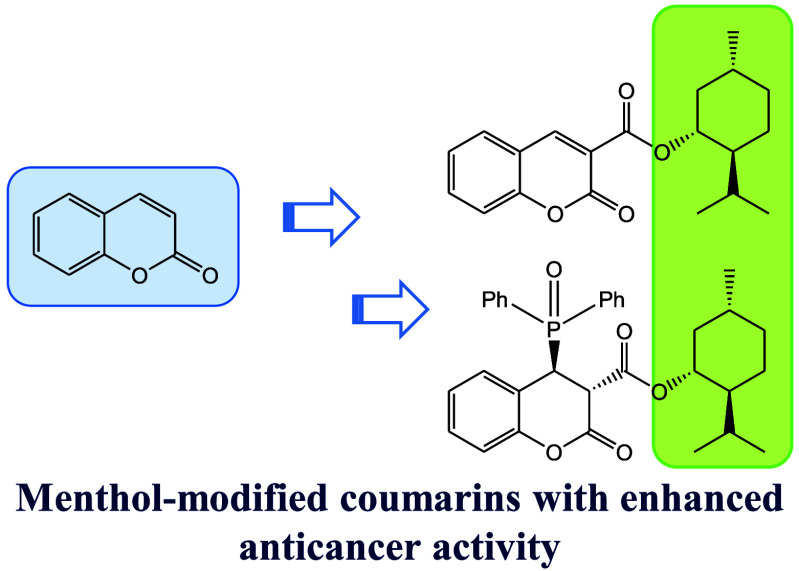

## 1 Introduction

Cancer is one of the most important challenges of today’s medicine. This is related to their ever-increasing incidence, as well as limitations related to the effectiveness and availability of therapies. The main methods of cancer treatment, such as surgery, radiotherapy, chemotherapy, or immunotherapy, cannot completely cure or eliminate the disease. Searching for new compounds with anticancer potential, which can function as single agents or in combination therapy, is highly valuable and desirable. Among the numerous biologically active compounds, our attention is focused on coumarins-natural and synthetic organic compounds with a variety of pharmacological properties including anticancer ones.^1^

Coumarins are key scaffolds in anticancer drug development. Due to capacity to interact with a variety of molecular signaling pathways, they inhibit the growth of human cancer cell lines. Mechanisms of action of coumarin derivatives include inhibition of tumor cell proliferation, modulation of signaling pathways, inhibition of angiogenesis, or induction of apoptosis.^2^ It has been shown, for example, that coumarins can decrease the expression of oncogenes, generate free radical-mediated oxidative stress in cancer cells resulting in a proapoptotic effect, and suppress tumor cell proliferation by arresting cell cycles in the G0/G1 and G2/M phases.^3^ Modifications of coumarins that incorporate a different pharmacophore group into their structure have garnered attention recently. A well-designed modification can produce a compound with multidirectional activity and improve its pharmacokinetic properties such as lipophilicity and bioavailability. Numerous examples of hybrid compounds with a coumarin backbone that demonstrate promising anticancer activity are known from the literature, (Figure 1).^4^

**Figure 1:**
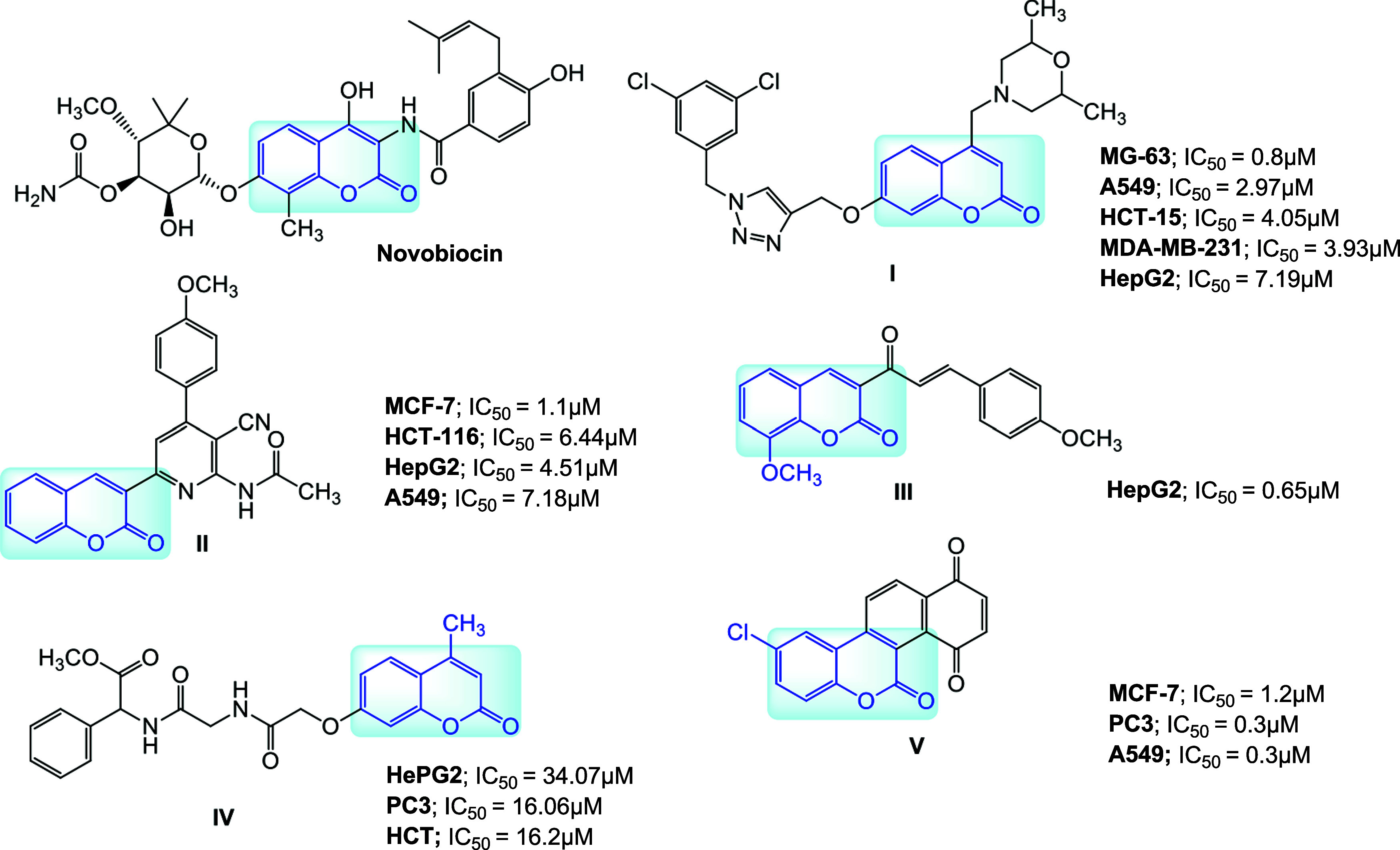
Chemical structures and cytotoxic activity of important coumarin hybrids (see text for explanation).

One example is **novobiocin**, a natural compound consisting of a noviose sugar, a coumarin backbone, and a prenylated benzamide side chain (Figure 1). This compound is registered as an antibiotic but has recently been shown to kill tumors that had become resistant to a PARP inhibitor (cells with mutations in the BRCA1 or BRCA2 genes). 1,2,3-triazoles and 1,2,4-triazoles have different pharmacological properties and are often selected for hybridization with coumarins to enhance their anticancer properties. Goud et al. presented a biological evaluation of morpholines-linked coumarin-triazole hybrids.^5^ Among them, compound **I** (Figure 1) was found to be the most effective against human cancer cell lines: bone (MG-63), lung (A549), colon (HCT-15), breast (MDA-MB-231), and liver (HepG2). Fayed et al. developed coumarin-pyridine hybrids.^6^ Their anticancer efficacy was assessed against human cancer cell lines MCF-7, HCT-116, HepG-2, and A549. Compound **II** had the most significant growth inhibiting activity. Subsequent biological evaluations revealed that the coumarin-pyridine hybrids induce cell cycle arrest at the G2/M phase in MCF-7 cells and promote apoptosis via the activation of caspase-3. It is also worth mentioning that other coumarin hybrids, such as coumarins with chalcones, (**III**),^7^ coumarins with amino acids, (**IV**),^8^ or coumarin-quinolinone hybrids, (**V**),^9^ also display promising anticancer potential.

Menthol is a compound with a broad spectrum of biological activity.^10^ It is utilized in various ailments, including inflammatory illnesses, pain disorders, respiratory disorders, cardiovascular diseases, and dermatological diseases. This natural terpene is also of interest to scientists as a potential anticancer compound. In vitro studies have demonstrated its ability to induce apoptosis in cancer cells, inhibit angiogenesis, and inhibit the cell cycle and its antiproliferative potential against cells such as prostate cancer, skin cancer, gastric cancer, liver cancer, bladder cancer, and leukemia.^11^

Considering the appealing scaffold of coumarin and the anticancer activity of the menthol molecule, we synthesized menthyl esters of coumarin-3-carboxylic acid. The anticancer activity of the compounds was assessed against four cancer cell lines, as well as their cytotoxic effects on human normal cells and *Danio rerio* larvae. We also evaluated the anticancer activity of known 3-phosphorylated coumarins. Subsequently, we incorporated a phosphoryl group at the C-4 position of the coumarin backbone, thus accessing a novel and scarcely explored subclass of 3,4-dihydrocoumarin derivatives (Figure 2). The variety of compounds for bioassays allowed the identification of structural elements that improve the properties of the molecules and enabled the study of synergies between the coumarin backbone, the menthyl group, and the phosphoryl fragment. The research incorporated predictions of physicochemical properties, ADME parameters (absorption, distribution, metabolism, and excretion), and the pharmacokinetic profiles of the compounds employing *in silico* methods.

**Figure 2:**
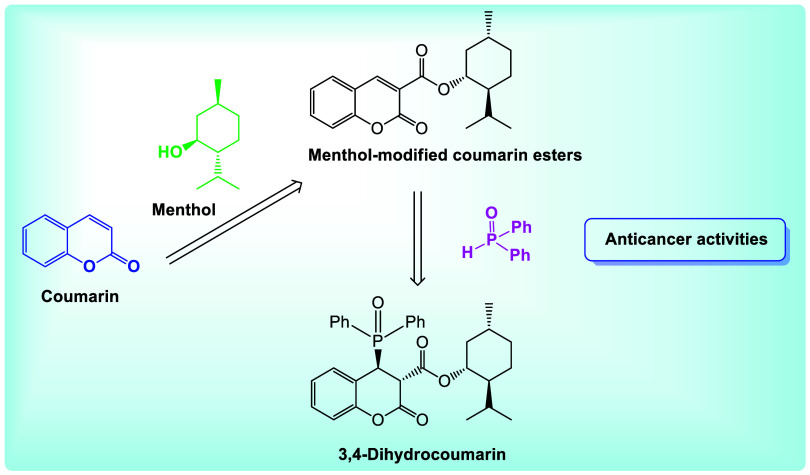
Coumarin hybrid with menthol and fused diphenyl phosphine oxide motif.

## 2 Materials and Methods

### 2.1 Chemistry

#### 2.1.1 Materials

All commercially available chemicals and solvents were acquired in good quality and utilized without additional purification. NMR spectra were recorded using a Bruker AV500 (^1^H 500 MHz, ^13^C 126 MHz, ^31^P 202 MHz) spectrometer. All spectra were obtained in CDCl_3_ solutions, and the chemical shifts (δ) were expressed in ppm using internal reference to TMS. Coupling constants (*J*) were given in Hz. The abbreviations of signal patterns were as follows: s, singlet; d, doublet; t, triplet; q, quartet; m, multiplet; b, broad. Optical rotations were measured on a PerkinElmer 341LC digital polarimeter. Melting points were determined on a Buchi 510 apparatus. Thin-layer chromatography (TLC) was done on silica gel (Kieselgel 60, F254 on aluminum sheets, Merck) using UV light (254 nm). HPLC–HRMS was performed on a Shimadzu LCMS-8030 LCMS System using a reverse-phase stationary phase with water/MeCN (65:35) as an eluent, electrospray ionization (ESI), and an IT-TOF detector (Shimadzu Europa, Duisburg, Germany). All column chromatographic separations and purifications were conducted using Merck silica gel 60 (230–400 mesh).

Compounds **1c**- **1e**, **4a**–**4c**, and **5a**–**5b** are newly synthesized derivatives, reported here for the first time.

#### 2.1.2 Experimental Procedures

##### 2.1.2.1 General Procedure for the Synthesis of Coumarin Menthyl Esters **1a**–**f**

To a solution of coumarin-3-carboxylic acid (1.0 g, 5.26 mmol) in dry dichloromethane (DCM, 15 mL) *N,N*′-dicyclohexylcarbodiimide (DCC, 1.7 equiv 8.94 mmol), 4-dimethyl-amino-pyridine (DMAP, 5.0 mol %, 0.26 mmol), and menthol or cyclohexanol (2.0 equiv 10.25 mmol) were added. The reaction mixture was stirred at room temperature for 12 h. The resulting byproduct DCU (*N,N′*-dicyclohexylurea) precipitate was eliminated from the reaction mixture by filtration and the filtrate was concentrated. Excess menthol was removed by sublimation and excess cyclohexanol was distilled off. The resulting coumarin esters were purified by column chromatography (hexane/ethyl acetate 15:1) and/or by recrystallization with ethanol.

##### 2.1.2.2 (*1R,2S*,5*R*)-2-Isopropyl-5-methylcyclohexyl 2-oxo-2*H*-chromene-3-carboxylate (**1a**)

Yield 61%, *R*_f_ = 0.6 (hexane/ethyl acetate 5:1), [α]_20_^D^ −58.5 (*c* 0.55, DCM), mp 144–146 °C. ^1^H NMR (500 MHz): δ 8.49 (s, 1H), 7.69–7.62 (m, 2H), 7.41–7.33 (m, 2H), 4.99 (td, *J* = 10.9, 4.4 Hz, 1H), 2.20–1.98 (m, 2H), 1.81–1.53 (m, 6H), 1.23–1.07 (m, 1H), 0.97 (d, *J* = 3.4 Hz, 3H), 0.95 (d, *J* = 3.8 Hz, 3H), 0.83 (d, *J* = 6.9 Hz, 3H). ^13^C NMR (126 MHz): δ 162.5, 156.6, 155.1, 147.9, 134.1, 129.4, 124.7, 118.7, 117.9, 116.7, 76.0, 46.9, 40.7, 34.1, 31.4, 26.1, 23.3, 22.0, 20.8, 16.2. The NMR signal patterns are consistent with previously reported data.^12^

##### 2.1.2.3 (1*S*,2*R*,5*S*)-2-Isopropyl-5-methylcyclohexyl 2-oxo-2*H*-chromene-3-carboxylate (**1b**)

Yield 58%, [α]_20_^D^ +59.3 (*c* 0.60, DCM), *R*_f_ = 0.6 (hexane/ethyl acetate 5:1), mp 145–146 °C. ^1^H NMR (500 MHz): δ 8.49 (s, 1H), 7.71–7.61 (m, 2H), 7.39–7.33 (m, 2H), 4.99 (td, *J* = 10.9, 4.4 Hz, 1H), 2.20–2.15 (m, 1H), 2.08–1.98 (m, 1H), 1.84–1.70 (m, 2H), 1.66–1.53 (m, 3H), 1.22–1.10 (m, 2H), 0.97 (d, *J* = 3.4 Hz, 3H), 0.96 (d, *J* = 3.9 Hz, 3H), 0.83 (d, *J* = 6.9 Hz, 3H). ^13^C NMR (126 MHz): δ 162.5, 156.5, 155.0, 147.9, 134.1, 129.4, 124.7, 118.7, 117.9, 116.6, 76.0, 46.9, 40.7, 34.1, 31.4, 26.1, 23.3, 22.0, 20.8, 16.2. The NMR signal patterns are consistent with previously reported data.^12^

##### 2.1.2.4 (1*R*,2*S*,5*R*)-2-Isopropyl-5-methylcyclohexyl 7-methoxy-2-oxo-2*H*-chromene-3-carboxylate (**1c**)

Yield 66%, *R*_f_ = 0.6 (hexane/ethyl acetate 5:1), [α]_20_^D^ −104.5 (*c* 0.7, DCM), mp 102–103 °C. ^1^H NMR (500 MHz): δ 8.47 (s, 1H), 7.53 (d, *J* = 8.7 Hz, 1H), 6.91 (dd, *J* = 8.7, 2.4 Hz, 1H), 4.97 (d, *J* = 4.4 Hz, 1H), 3.93 (s, 3H), 2.20–1.95 (m, 2H), 1.80–1.71 (m, 2H), 1.62–1.54 (m, 3H), 1.15 (t, *J* = 11.7 Hz, 2H), 0.96 (dd, *J* = 6.8, 1.9 Hz, 6H), 0.82 (d, *J* = 6.9 Hz, 3H). ^13^C NMR (126 MHz, CDCl_3_) δ 164.99, 162.91, 157.53, 157.03, 148.38, 130.63, 114.63, 113.54, 111.66, 100.32, 75.70, 55.99, 47.05, 40.80, 34.23, 31.48, 26.21, 23.35, 22.04, 20.88, 16.26. HRMS (ESI-IT-TOF) *m*/*z* calcd for C_22_H_27_O_5_ [M + H]^+^: 359.1858; found: 359.1859.

##### 2.1.2.5 (1*R*,2*S*,5*R*)-2-Isopropyl-5-methylcyclohexyl 8-Methoxy-2-oxo-2*H*-chromene-3-carboxylate (**1d**)

Yield 72%, *R*_f_ = 0.6 (hexane/ethyl acetate 5:1), [α]_20_^D^ −78.0 (*c* 1.0, DCM), mp 108–109 °C. ^1^H NMR (500 MHz): δ 8.42 (s, 1H), 7.33–7.22 (m, 1H), 7.17–7.15 (m, 2H), 4.93 (td, *J* = 10.9, 4.4 Hz, 1H), 3.95 (s, 3H), 2.16–2.09 (m, 1H), 2.02–1.96 (m, 1H), 1.76–1.66 (m, 2H), 1.60–1.48 (m, 2H), 1.17–1.08 (m, 2H), 0.91 (dd, *J* = 6.8, 3.6 Hz, 6H), 0.79 (d, *J* = 6.9 Hz, 3H). ^13^C NMR (126 MHz): δ 162.51, 156.05, 148.11, 147.03, 144.81, 124.60, 120.57, 118.91, 118.46, 115.78, 77.36, 77.11, 76.86, 75.98, 56.32, 46.96, 40.70, 34.17, 31.45, 26.20, 23.34, 22.00, 20.82, 16.26. ^13^C NMR (126 MHz) δ 162.51, 156.05, 148.11, 147.03, 144.81, 124.60, 120.57, 118.91, 118.46, 115.78, 77.36, 77.11, 76.86, 75.98, 56.32, 46.96, 40.70, 34.17, 31.45, 26.20, 23.34, 22.00, 20.82, 16.26. HRMS (ESI-IT-TOF) *m*/*z* calcd for C_22_H_27_O_5_ [M + H]^+^: 359.1858; found: 359.1857.

##### 2.1.2.6 (1*R*,2*S*,5*R*)-2-Isopropyl-5-methylcyclohexyl 7-hydroxy-2-oxo-2*H*-chromene-3-carboxylate (**1e**)

Yield 45%, *R*_f_ = 0.6 (hexane/ethyl acetate 5:1), [α]_20_^D^ −120.8 (*c* 1.0, DCM), mp 198–199 °C. ^1^H NMR (500 MHz): δ 8.56 (s, 1 H) 7.53 (d, *J* = 8.83 Hz, 1 H) 7.06 (d, *J* = 2.21 Hz, 1 H) 7.00 (dd, *J* = 8.51, 2.21 Hz, 1 H) 4.98 (td, *J* = 10.88, 4.41 Hz, 1 H) 2.09–2.18 (m, 1 H) 1.99 (td, *J* = 6.94, 2.84 Hz, 1 H) 1.69–1.77 (m, 2 H) 1.51–1.63 (m, 2 H) 1.07–1.20 (m, 2 H) 0.93 (dd, *J* = 6.78, 3.00 Hz, 6 H) 0.81 (d, *J* = 6.94 Hz, 3 H). ^13^C NMR (126 MHz): δ 163.9, 163.1, 158.8, 157.3, 149.8, 131.3, 115.1, 112.7, 111.3, 103.2, 75.9, 47.0, 40.8, 34.2, 31.5, 26.3, 23.4, 22.0, 20.8, 16.3. HRMS (ESI-IT-TOF) *m*/*z* calcd for C_21_H_25_O_5_ [M + H]^+^: 345.1702; found: 345.1702.

##### 2.1.2.7 Cyclohexyl 2-Oxo-2*H*-chromene-3-carboxylate (**1f**)

Yield 68%, *R*_f_ = 0.5 (hexane/ethyl acetate 5:1), mp 107–108 °C, ^1^H NMR (500 MHz,): δ 8.49 (s, 1H), 7.68–7.63 (m, 2H), 7.40–7.36 (m, 2H), 5.09–5.06 (m, 1H), 2.02–1.28 (m, 10H). ^13^C NMR (126 MHz): δ 162.4, 156.7, 155.1, 147.9, 134.1, 129.4, 124.7, 118.9, 117.9, 116.8, 74.4, 56.9, 31.5, 25.4, 23.6. The NMR signal patterns are consistent with previously reported data.^12^

##### 2.1.2.8 General Procedure for the Synthesis of 3-Phosphorylated Coumarins (**2a**, **2b**)

2-(Diethoxyphosphoryl)­acetate (1.0 mmol), 2-hydroxybenzaldehyde or 3-methoxy-2-hydroxybenzaldehyde (1.5 mmol, 1.5 equiv) with piperidine (30 mol %) and acetic acid (30 mol %) were dissolved in 15 mL of acetonitrile and refluxed for 18 h. The reaction mixture was then allowed to cool and was treated with an aqueous saturated NaHCO_3_ solution (25 mL). The organic layer was collected, dried, and concentrated under vacuum, and purified using column chromatography, eluting with CHCl_3_/ethyl acetate (24:1).

##### 2.1.2.9 Diethyl (2-Oxo-2*H*-chromen-3-yl)­phosphonate (**2a**)

Yield 70%, *R*_f_ = 0.4 (DMC/CH_3_OH 50:1), pale yellow oil. ^1^H NMR (500 MH): δ 8.51 (d, *J* = 17.2 Hz, 1H), 7.63–7.58 (m, 2H), 7.38–7.30 (m, 2H), 4.33–4.17 (m, 4H), 1.36 (td, *J* = 7.1, 0.5 Hz, 6H). ^13^C NMR (126 MHz): δ 158.2 (d, *J* = 14.4 Hz), 155.2, 153.5 (d, *J* = 6.7 Hz), 134.2, 129.7, 124.9, 118.5, 117.9 (d, *J* = 14.2 Hz), 116.9, 116.8, 116.6, 63.43 (d, *J* = 6.0 Hz), 16.3 (d, *J* = 6.3 Hz). ^31^P NMR (202 MHz): δ 10.92 (s). The NMR signal patterns are consistent with previously reported data.^13^

##### 2.1.2.10 Diethyl (8-Methoxy-2-oxo-2*H*-chromen-3-yl)­phosphonate (**2b**)

Yield 84%, *R*_f_ = 0.4 (DMC/CH_3_OH 50:1), viscous oil. ^1^H NMR (500 MHz): δ 8.32 (d, *J* = 17.2 Hz, 1H), 7.18–6.94 (m, 3H), 4.21–4.04 (m, 4H), 3.82 (s, 3H), 1.23 (t, *J* = 7.1 Hz, 6H). ^13^C NMR (126 MHz): δ 157.5 (d, *J* = 14.7 Hz), 153.4 (d, *J* = 6.5 Hz), 146.9, 144.6, 124.7, 120.33, 120.3, 118.5, 118.3 (d, *J* = 14.0 Hz), 116.9, 115.78, 63.3 (d, *J* = 6.0 Hz), 56.2, 16.3 (d, *J* = 6.3 Hz). ^31^P NMR (202 MHz): δ 10.89 (s). The NMR signal patterns are consistent with previously reported data^13^

##### 2.1.2.11 Synthesis of 3-(Diphenylphosphinyl)-8-methoxy-2*H*-chromen-2-one (**3**)

2-(Diphenylphosphoryl)­acetic acid (1.0 mmol), 3-methoxy-2-hydroxybenzaldehyde (1.5 mmol, 1.5 equiv) with piperidine (30 mol %) and acetic acid (30 mol %) were dissolved in 15 mL of acetonitrile and refluxed for 18 h. The reaction mixture was then allowed to cool and was treated with an aqueous saturated NaHCO_3_ solution (25 mL). The organic layer was collected, dried, and concentrated under vacuum, and purified using column chromatography, eluting with DCM/CH_3_OH (50:1). Yield 90%, *R*_f_ = 0.5 (DCM/CH_3_OH 20:1), mp 210–211 °C. ^1^H NMR (500 MHz): δ 8.90 (d, *J* = 14.2 Hz, 1H), 7.94–7.90 (m, 4H), 7.60–7.56 (m, 2H), 7.51–7.47 (m, 4H), 7.30–7.25 (m, 2H), 7.18 (dd, *J* = 7.6 and 1.9 Hz, 1H), 3.96 (s, 3H). ^13^C NMR (126 MHz): δ 158.6 (d, *J* = 13.6 Hz), 154.2 (d, *J* = 4.5 Hz), 147.2, 145.1 (d, *J* = 1.8 Hz), 132.4 (d, *J* = 3.1 Hz), 132.1 (d, *J* = 11.7 Hz), 130.5 (d, *J* = 109.9 Hz), 128.5 (d, *J* = 12.7 Hz), 124.8, 121.8 (d, *J* = 101.7 Hz), 120.7, 119.1 (d, *J* = 11.8 Hz), 115.8, 56.4. ^31^P NMR (202 MHz): δ 23.28 (s). The NMR signal patterns are consistent with previously reported data^14^

##### 2.1.2.12 General Procedure for the H–P­(O)­Ph_2_ Addition to the Coumarin Core

To a solution of coumarin **1–2** (0.5 g) in CH_3_CN (10 mL), H–P­(O)­Ph_2_ (1.0 equiv) was added. The reaction mixture was stirred at room temperature for 4–8 h. Then CH_3_CN was evaporated to give the white solid of the product. In the case of a reaction carried out in water, the product was filtered off and the water was extracted twice with DCM.

##### 2.1.2.13 (1*R*,2*S*,5*R*)-2-Isopropyl-5-methylcyclohexyl-4-(diphenylphosphoryl)-2-oxochromane-3-carboxylate (**4a**)

Compound **4a** was obtained as a mixture of 2 diastreoisomers (*dr* 1:1.2). Yield 90%, *R*_f_ = 0.5 (DCM/CH_3_OH 10:1), mp 107–108 °C.

##### 2.1.2.14 Major Isomer (1*R*,2*S*,5*R*)-2-Isopropyl-5-methylcyclohexyl (4*R*)-4-(Diphenylphosphoryl)-2-oxochromane-3-carboxylate (*trans*)

^1^H NMR (500 MHz): δ 8.02–7.90 (m, 2H), 7.74–7.48 (m, 6H), 7.46–7.39 (m, 2H), 7.31–7.21 (m, 1H), 7.09–7.02 (m, 1H), 6.86 (dd, *J* = 17.5, 7.5 Hz, 1H), 6.53 (dd, *J* = 7.7, 6.2 Hz, 1H), 4.57 (dt, *J* = 7.9, 5.2 Hz, 1H), 4.36 (t, *J* = 10.7 Hz, 1H), 4.12 (dd, *J* = 9.4, 1.2 Hz, 1H), 1.88–1.83 (m, 1H), 1.69–1.51 (m, 3H), 1.42–1.22 (m, 2H), 1.03–0.90 (m, 2H), 0.90–0.72 (m, 6H), 0.62 (dd, *J* = 10.4, 6.9 Hz, 3H), 0.23 (d, *J* = 6.9 Hz, 1H).

##### 2.1.2.15 Minor Isomer (1*R*,2*S*,5*R*)-2-Isopropyl-5-methylcyclohexyl (4S)-4-(diphenylphosphoryl)-2-oxochromane-3-carboxylate (*cis*)

^1^H NMR (500 MHz): δ 8.02–7.90 (m, 2H), 7.74–7.48 (m, 6H), 7.46–7.39 (m, 2H), 7.31–7.21 (m, 1H), 7.09–7.02 (m, 1H), 6.86 (dd, *J* = 17.5, 7.5 Hz, 1H), 6.53 (dd, *J* = 7.7, 6.2 Hz, 1H), 4.57 (dt, *J* = 7.9, 5.2 Hz, 1H), 4.36 (t, *J* = 10.7 Hz, 1H), 4.09 (dd, *J* = 9.7, 0.95 Hz, 1H), 1.88–1.83 (m, 1H), 1.69–1.51 (m, 3H), 1.42–1.22 (m, 2H), 1.03–0.90 (m, 2H), 0.90–0.72 (m, 6H), 0.62 (dd, *J* = 10.4, 6.9 Hz, 3H), 0.23 (d, *J* = 6.9 Hz, 1H).

^13^C NMR (126 MHz): δ 166.7 (d, *J* = 15.2 Hz), 166.4 (d, *J* = 15.2 Hz), 162.1, 162.0, (d, *J* = 7.4 Hz), 152.2 (d, *J* = 4.0 Hz), 152.1 (d, *J* = 4.1 Hz), 132.8 (d, *J* = 2.4 Hz), 132.7 (d, *J* = 2.5 Hz), 132.6 (d, *J* = 2.9 Hz), 131.8, 131.7 (d, *J* = 1.2 Hz), 131.6, 131.6, 131.5, 129.7 (d, *J* = 3.7 Hz), 129.6, 129.6, 129.6, 129.5, 129.5, 129.3 (d, *J* = 2.7 Hz), 129.2 (d, *J* = 2.8 Hz), 128.8, 128.5 (d, *J* = 2.1 Hz), 128.4 (d, *J* = 2.2 Hz), 117.6, 117.5, 117.50, 115.4 (d, *J* = 4.8 Hz), 115.3 (d, *J* = 5.1 Hz), 46.5, 46.50, 46.4, 46.3, 42.6 (d, *J* = 19.0 Hz), 42.1 (d, *J* = 19.4 Hz), 40.1, 39.8, 33.8, 31.4, 31.1,30.9, 25.9, 25.2, 23.0, 22.5, 21.8, 21.7, 20.7, 20.6, 15.9, 15.0. ^31^P NMR (202 MHz): δ 30.44 (s), 29.92 (s). HRMS (ESI-IT-TOF) *m*/*z* calcd for C_32_H_36_O_5_P [M + H]^+^: 531.23004; found: 531.23014. Crystal data **4a**: crystal system monoclinic, space group *P*2_1_; unit cell dimensions *a* = 21.0777(9) Å, *b* = 6.2017(3) Å, *c* = 23.2389(9) Å, β = 107.06(1)°, *V* = 2904.1(3) Å^3^, *Z* = 4, *F*(000) = 1128. Cu Kα, λ = 1.54184 Å, index ranges −26 ≤ *h* ≤ 26, −7 ≤ *k* ≤ 4, −27 ≤ *l* ≤ 28; reflections collected/independent 20278/8705 [*R*(int) = 0.1236]. Goodness-of-fit on *F* ^2^ 1.066, final *R* indices [*I* > 2σ­(*I*)] *R*_1_ = 0.0722, w*R*_2_ = 0.1736. Deposition Number CCDC No. 2410610 (These data are provided free of charge by the joint Cambridge Crystallographic Data Centre).

##### 2.1.2.16 (1*R*,2*S*,5*R*)-2-Isopropyl-5-methylcyclohexyl-4-(diphenylphosphoryl)-8-methoxy-2-oxochromane-3-carboxylate (**4b**)

Compound **4b** was obtained as a mixture of 2 diastreoisomers (*dr* 1:1.1). Yield 93%, *R*_f_ = 0.45 (DCM/CH_3_OH 10:1), mp 107–108 °C.

##### 2.1.2.17 Major Isomer (1*R*,2*S*,5*R*)-2-Isopropyl-5-methylcyclohexyl (4*R*)-4-(Diphenylphosphoryl)-8-methoxy-2-oxochromane-3-carboxylate (*trans*)

^1^H NMR (500 MHz) δ 7.97–7.88 (m, 2H), 7.64–7.52 (m, 6H), 7.43–7.39 (m, 2H), 6.85–6.74 (m, 2H), 6.14–6.08 (m, 1H), 4.55 (qd, *J* = 10.8, 4.3 Hz, 1H), 4.38 (d, *J* = 8.8 Hz, 1H), 4.08 (dd, *J* = 9.1, 1.2 Hz, 1H), 3.84 (s, 3H), 1.88–1.80 (m, 1H), 1.67–1.55 (m, 2H), 1.39–1.22 (m, 3H), 1.10–1.02 (m, 1H), 0.87–0.72 (m, 6H), 0.85 (d, *J* = 6.6 Hz, 3H), 0.19 (d, *J* = 6.9 Hz, 2H).

##### 2.1.2.18 Minor Isomer (1*R*,2*S*,5*R*)-2-Isopropyl-5-methylcyclohexyl (4*S*)-4-(Diphenylphosphoryl)-8-methoxy-2-oxochromane-3-carboxylate (*cis*)

^1^H NMR (500 MHz) δ 7.97–7.88 (m, 2H), 7.64–7.52 (m, 6H), 7.43–7.39 (m, 2H), 6.85–6.74 (m, 2H), 6.14–6.08 (m, 1H), 4.55 (qd, *J* = 10.8, 4.3 Hz, 1H), 4.34 (dd, *J* = 10.0, 0.9 Hz, 1H), 4.04 (dd, *J* = 9.4, 0.9 Hz, 1H), 3.84 (s, 3H), 1.88–1.80 (m, 1H), 1.67–1.55 (m, 2H), 1.39–1.22 (m, 3H), 1.10–1.02 (m, 1H), 0.87–0.72 (m, 6H), 0.85 (d, *J* = 6.6 Hz, 3H), 0.19 (d, *J* = 6.9 Hz, 2H).

^13^C NMR (126 MHz,): δ 166.7 (d, *J* = 17.6 Hz), 166.5 (d, *J* = 17.2 Hz), 161.5 (d, *J* = 1.5 Hz), 148.0 (d, *J* = 18.5 Hz), 141.7, 141.5, 132.7 (d, *J* = 5.6 Hz), 132.6 (d, *J* = 2.7 Hz), 132.5 (d, *J* = 2.8 Hz), 131.8, 131.6, 131.5, 129.2 (d, *J* = 2.7 Hz), 129.1 (d, *J* = 2.7 Hz), 128.5 (d, *J* = 4.1 Hz), 128.4 (d, *J* = 4.1 Hz), 123.9 (d, *J* = 5.8 Hz), 121.0 (d, *J* = 8.9 Hz), 116.5 (d, *J* = 48.1 Hz), 112.2 (d, *J* = 59.6 Hz), 56.0 (d, *J* = 42.2 Hz), 42.8 (d, *J* = 19.2 Hz), 42.3 (d, *J* = 19.9 Hz), 40.1, 39.81, 33.8, 31.4, 31.1, 26.9, 25.9, 25.2, 23.0, 22.4, 21.8 (d, *J* = 2.6 Hz), 20.7, 20.6, 15.9, 14.92. ^31^P NMR (202 MHz): δ 30.30 (s), 29.69 (s). HRMS (ESI-IT-TOF) *m*/*z* calcd for C_33_H_38_O_6_P [M + H]^+^: 561.24060; found: 561.24067

##### 2.1.2.19 Ethyl 4-(Diphenylphosphoryl)-2-oxochroman-3-carboxylate (**4c**)

Yield 98%, *R*_f_ = 0.40 (DCM/CH_3_OH 10:1), mp 182–183 °C. ^1^H NMR (500 MHz): δ 7.99–7.91 (m, 2H), 7.68–7.37 (m, 6H), 7.28–7.22 (m, 4H), 7.07 (d, *J* = 8.1 Hz, 1H), 6.86 (t, *J* = 7.5 Hz, 1H), 6.54 (d, *J* = 7.7 Hz, 1H), 4.38 (dd, *J* = 10.0 Hz, 1H), 4.15–4.08 (m, 2H), 1.09 (t, *J* = 7.1 Hz, 3H). ^13^C NMR (126 MHz): δ 166.7 (d, *J* = 17.3 Hz), 161.8, 152.0 (d, *J* = 4.2 Hz), 132.7 (d, *J* = 18.5, Hz), 131.6 (d, *J* = 9.0 Hz), 129.6, 129.1, 129.2 (d, *J* = 11.7 Hz), 128.8, 128.5 (d, *J* = 11.9 Hz), 128.0, 124.1 (d, *J* = 2.6 Hz), 117.5 (d, *J* = 2.5 Hz), 115.3 (d, *J* = 5.0 Hz), 62.9, 46.2, 42.2, 41.7, 13.8. ^31^P NMR (202 MHz): δ 30.37 (s). HRMS (ESI-IT-TOF) *m*/*z* calcd for C_24_H_22_O_5_P [M + H]^+^: 421.1204; found: 421.1205.

##### 2.1.2.20 Diethyl (4-(Diphenylphosphoryl)-2-oxochroman-3-yl)­phosphonate (**5a**)

Yield 89%, *R*_f_ = 0.40 (DCM/CH_3_OH 10:1), mp 182–183 °C. In order to obtain a pure product, an additional crystallization from hexane/DCM was performed. 1H NMR (500 MHz) δ 7.97–7.90 (m, 1H), 7.69–7.36 (m, 7H), 7.25 (t, *J* = 8.5 Hz, 1H), 7.02 (d, *J* = 7.8 Hz, 1H), 6.85 (t, *J* = 7.5 Hz, 1H), 6.55 (d, *J* = 7.7 Hz, 1H), 4.44 (dd, *J* = 17.7, 9.7 Hz, 1H), 4.18–4.04 (m, 1H), 3.86–3.70 (m, 1H), 3.58–3.45 (m, 1H), 1.30 (td, *J* = 7.0, 0.6 Hz, 3H), 0.88 (td, *J* = 7.1, 0.4 Hz, 3H). ^13^C NMR (126 MHz): δ 161.6, 152.5, 132.8 (d, *J* = 2.7 Hz), 132.6 (d, *J* = 2.7 Hz), 131.8, 131.7, 131.71, 131.6, 129.5 (d, *J* = 3.9 Hz), 129.4, 129.2, 129.2, 128.4 (d, *J* = 12.0 Hz), 123.9, 117.1 (d, *J* = 2.8 Hz), 115.0, 63.8 (d, *J* = 6.8 Hz), 63.3 (d, *J* = 7.1 Hz), 39.8, 39.3, 38.8, 16.1 (d, *J* = 6.2 Hz), 15.7 (d, *J* = 6.3 Hz). ^31^P NMR (202 MHz): δ 32.5 (d, *J* = 56.5 Hz), 18.5 (d, *J* = 56.5 Hz). HRMS (ESI-IT-TOF) *m*/*z* calcd for C_25_H_27_O_6_P_2_ [M + H]^+^: 485.12829; found: 4851.12819.

##### 2.1.2.21 Diethyl (4-(Diphenylphosphoryl)-8-methoxy-2-oxochroman-3-yl)­phosphonate (**5b**)

Yield 92%, *R*_f_ = 0.5 (DCM/CH_3_OH 10:1), mp 182–183 °C. ^1^H NMR (500 MHz): δ 8.03–7.90 (m, 2H), 7.81–7.72 (m, 2H), 7.66–7.49 (m, 6H), 7.41–7.37 (m, 2H), 6.90–6.72 (m, 2H), 6.14 (d, *J* = 7.6 Hz, 1H), 4.44 (dd, *J* = 17.7, 9.9 Hz, 1H), 4.12 (ddd, *J* = 8.3, 7.1, 3.7 Hz, 1H), 3.88 (s, 3H), 3.83–3.70 (m, 1H), 3.59–3.51 (m, 1H), 1.29 (t, *J* = 7.0 Hz, 3H), 0.89 (t, *J* = 7.0 Hz, 3H). ^13^C NMR (126 MHz): δ 161.0 (d, *J* = 6.0 Hz), 147.6 (d, *J* = 2.8 Hz), 142.0 (d, *J* = 4.5 Hz), 132.8 (d, *J* = 2.7 Hz), 132.6 (d, *J* = 2.8 Hz), 131.8, 131.7, 131.6, 131.6, 130.7 (d, *J* = 11.5 Hz), 129.2 (d, *J* = 11.7 Hz), 128.9, 128.8, 128.4 (d, *J* = 11.9 Hz), 123.8 (d, *J* = 2.6 Hz), 121.0 (d, *J* = 3.8 Hz), 115.9 (d, *J* = 5.2 Hz), 112.3 (d, *J* = 2.9 Hz), 63.8 (d, *J* = 6.8 Hz), 63.3 (d, *J* = 7.1 Hz), 56.2, 39.74, 39.3, 38.9 (d, *J* = 4.7 Hz), 38.5, 16.1 (d, *J* = 6.2 Hz), 15.6 (d, *J* = 6.4 Hz). ^31^P NMR (202 MHz): δ 32.3 (d, *J* = 56.5 Hz), 18.3 (d, *J* = 56.5 Hz). HRMS (ESI-IT-TOF) *m*/*z* calcd for C_26_H_29_O_7_P_2_ [M + H]^+^: 515.13886; found: 515.13873.

##### 2.1.2.22 Synthesis of Ethyl 4-(diethoxyphosphoryl)-2-oxochroman-3-carboxylate (**4d**)

To a solution of ethyl 2-oxo-2H-chromene-3-carboxylate (0.5 g, 2.29 mmol) in CH_3_CN (20 mL), H–P­(O)­(OEt)_2_ (1.0 equiv, 0.316 g) and DABCO (20 mol %, 0.051 g) were added. The reaction mixture was stirred at room temperature for 12 h. Then CH_3_CN was evaporated and a mixture was purified by column chromatography (hexane/ethyl acetate 1:3). Yield 78%, *R*_f_ = 0.2 (hexane/ethyl acetate 1:1). ^1^H NMR (500 MHz): δ 7.33–7.29 (m, 2H), 7.19–7.14 (m, 1H), 7.09 (d, *J* = 8.1 Hz, 1H), 4.20–4.08 (m, 5H), 4.04–3.95 (m, 3H), 3.91 (d, *J* = 22.8 Hz, 1H), 1.32 (t, *J* = 7.1 Hz, 3H), 1.22 (t, *J* = 7.1 Hz, 3H), 1.11 (t, *J* = 7.1 Hz, 3H). ^13^C NMR (126 MHz): δ 166.2 (d, *J* = 21.6 Hz), 162.3 (d, *J* = 2.2 Hz), 151.2 (d, *J* = 5.5 Hz), 130.1 (d, *J* = 4.8 Hz), 129.7 (d, *J* = 3.6 Hz), 124.9 (d, *J* = 3.2 Hz), 117.1 (d, *J* = 3.3 Hz), 115.6 (d, *J* = 7.3 Hz), 63.6 (d, *J* = 7.2 Hz), 63.3 (d, *J* = 7.1 Hz), 62.9, 46.9 (d, *J* = 2.8 Hz), 39.1, 37.9, 16.3 (d, *J* = 5.6 Hz), 16.1 (d, *J* = 5.6 Hz), 13.8. ^31^P NMR (202 MHz): δ 21.4 (s). The NMR signal patterns are consistent with previously reported data.^15^

### 2.2 Biology

#### 2.2.1 Cell Cultures

The human primary (SW480) and metastatic (SW620) colon cancer, metastatic prostate cancer (PC3), breast adenocarcinoma (MDA-MB-231) and human immortal keratinocyte (HaCaT) cell lines were recruited from the American Type Culture Collection (ATCC). The cells were cultured in the recommended medium according to protocols (RPMI 1640 for PC3, MEM for SW480 and SW620, as well as DMEM for MDA-MB-231 and HaCaT cells) with addition of 10% FBS, penicillin (100 U/mL), and streptomycin (100 μg/mL) and cultured in 37 °C/5% CO_2_ in a humidified incubator. Then, the cells were passaged at a confluence of 80–90% by a treatment with 0.25% trypsin (Gibco Life Technologies) and used for studies.

#### 2.2.2 MTT Tests

Studied compounds and standard drugs-doxorubicin and cisplatin, were tested at various concentrations (ranged from 1 to 100 μM). They were added to 96-well plates with seeded normal and cancer cells (1 × 10^4^ cells per well) and incubated for 72 h. MTT analysis was performed according to a previous study.^16^

#### 2.2.3 Trypan Blue Assay

After 72 h incubation with IC_50_ concentrations of studied compounds **2b**, **4a**, **4b**, cells were washed twice with PBS (Phosphate Buffered Saline) and harvested. The total cell count was assessed by trypan blue exclusion dye assay using automated cell counter (Countess Invitrogen). Untreated cells were used as the control. Each experiment was repeated 5 times.

#### 2.2.4 Annexin Binding Assay

The apoptotic and necrotic cells treated with derivatives **2b**, **4a**, **4b** were detected using FITC Annexin V Apoptosis Detection Kit I (BD Biosciences Pharmingen). Briefly, cells (2 × 105 cells/well) were seeded in 6-well plates in supplemented medium. After 24 h, cells were incubated with studied compounds at their IC_50_ concentrations for 72 h. Then cells were harvested, washed, and labeled with Annexin V-FITC and propidium iodide (PI) according to the manufacturer’s protocol. The stained cells were analyzed by flow cytometry. The cells were identified as early apoptotic (Annexin V+/PI−) or late apoptotic/necrotic (Annexin V+/PI+).

#### 2.2.5 Interleukin-6 Assay

The concentration of Interleukin-6 (IL-6) was measured in cell culture supernatant using Interleukin IL-6 High Sensitivity ELISA kit (Diaclon SAS, Besancon Cedex, France). Cells (1 × 105 cells/well) were seeded in 12-well plates with medium for 24 h. Then, cells were treated with synthesized compounds **2b, 4a, 4b** at their IC_50_ concentrations for 72 h. After this incubation, the supernatants were collected and 100 μL of supernatant samples, control solutions and blank were added to precoated ELISA plate with the preceding plotting of the standard curve. Then 50 μL of diluted biotinylated antihuman interleukin-6 antibody was added and incubated at room temperature for 3 h. Afterward, the plate was washed three times and 100 μL streptavidin-HRP solution was added to each well. After 30 min incubation, the plate was washed again three times and 100 μL of tetramethylbenzidine (TMB) solution was added to each well. The plate was incubated for 15 min in the dark. The reaction was stopped by adding 100 μL of H_2_SO_4_ solution. The absorbance was estimated using a spectrophotometer at 450 nm (Microplate Spectrophotometer Thermo Scientific Multiskan GO).

#### 2.2.6 *In Vivo* Toxicity Assessment Using *Danio rerio* Larvae

##### 2.2.6.1 Zebrafish Maintenance

Zebrafish (*Danio rerio*, *strain AB*) was maintained at the Experimental Medicine Center, Medical University of Lublin, Poland. Embryos and larvae up to 120 h postfertilization (hpf) were kept under generally accepted environmental conditions, i.e., in incubators at 28.5 °C, with 14/10 h light/dark cycles. The embryos of the Zebrafish were harvested after the natural reproduction process.

##### 2.2.6.2 Ethical Declarations

The *in vivo* experiments were conducted in accordance with the National Institutes of Health guidelines for the care and use of laboratory animals and the European Community Council Directive on the Care and Use of Laboratory Animals of September 22, 2010. All methods involving zebrafish embryos and larvae were in compliance with Animal Research: Reporting of *In Vivo* Experiments (ARRIVE) guidelines. Approval from the Local Ethics Committee was not required for the experiment on larvae up to 120 h post fertilization (hpf). For euthanasia, a solution of tricaine methanesulfonate (MS-222) (Sigma-Aldrich, St. Louis, MO, USA) was employed immediately after completion of the experiments. The research methods are selected to minimize pain and suffering.

##### 2.2.6.3 Chemical Treatment

Three compounds showing low IC_50_ values determined on cancer lines in *in vitro* tests (**2b**, **4a**) and compound **1a** characterized by low cytotoxicity were used for *in vivo* tests. We also conducted research for coumarin. These compounds were dissolved in dimethyl sulfoxide (DMSO) (Sigma-Aldrich, St. Louis, MO, USA) and diluted in the zebrafish medium (pH 7.1–7.3; 5.0 mM NaCl, 0.17 mM KCl, 0.33 mM CaCl_2_, 0.33 mM MgSO_4_) to achieve a final DMSO concentration of 1% w/v. Twenty-four hpf zebrafish embryos were investigated under a light microscope, and 24 viable fertilized embryos (*n* = 60) were selected and transferred into 48-well plates (5 embryos per well). Larvae were incubated in 1 mL of the zebrafish medium for up to 3 days after fertilization.

##### 2.2.6.4 Determination of the Maximum Tolerable Concentration

On the fourth day postfertilization (96 hpf), the larvae (10 individuals per group) were incubated in solutions of coumarin or its three derivatives (**1a**, **2b**, **4a**) at selected concentrations (5, 10, 25, 50, 75, 100, 150 μM), along with control solutions: negative control (zebrafish medium) and solvent control (1% DMSO in zebrafish medium). The maximum tolerated concentration (MTC) was calculated to assess the toxicity of the compounds and extracts. MTC is defined as the highest concentration that neither caused mortality nor resulted in locomotor impairments, such as a diminished response to touch, after 18 h (120 hpf) of immersion in more than 2 out of 10 larvae. Larvae were individually inspected under a microscope for signs of acute locomotor dysfunction, including a weak reaction to light tail stimulation with a fine needle, disrupted posture, body deformities, protruding eyes, slowed or absent heartbeat, and mortality.

## 3 Results and Discussion

### 3.1 Chemistry

The sequence of reactions employed in the synthesis of new biologically active compounds based on the coumarin backbone is illustrated in Schemes 1–4. Full ^1^H NMR, ^13^C NMR, and ^31^P NMR were used to confirm the structures and purity of all synthesized compounds (see Supporting Information).

**Scheme 1:**
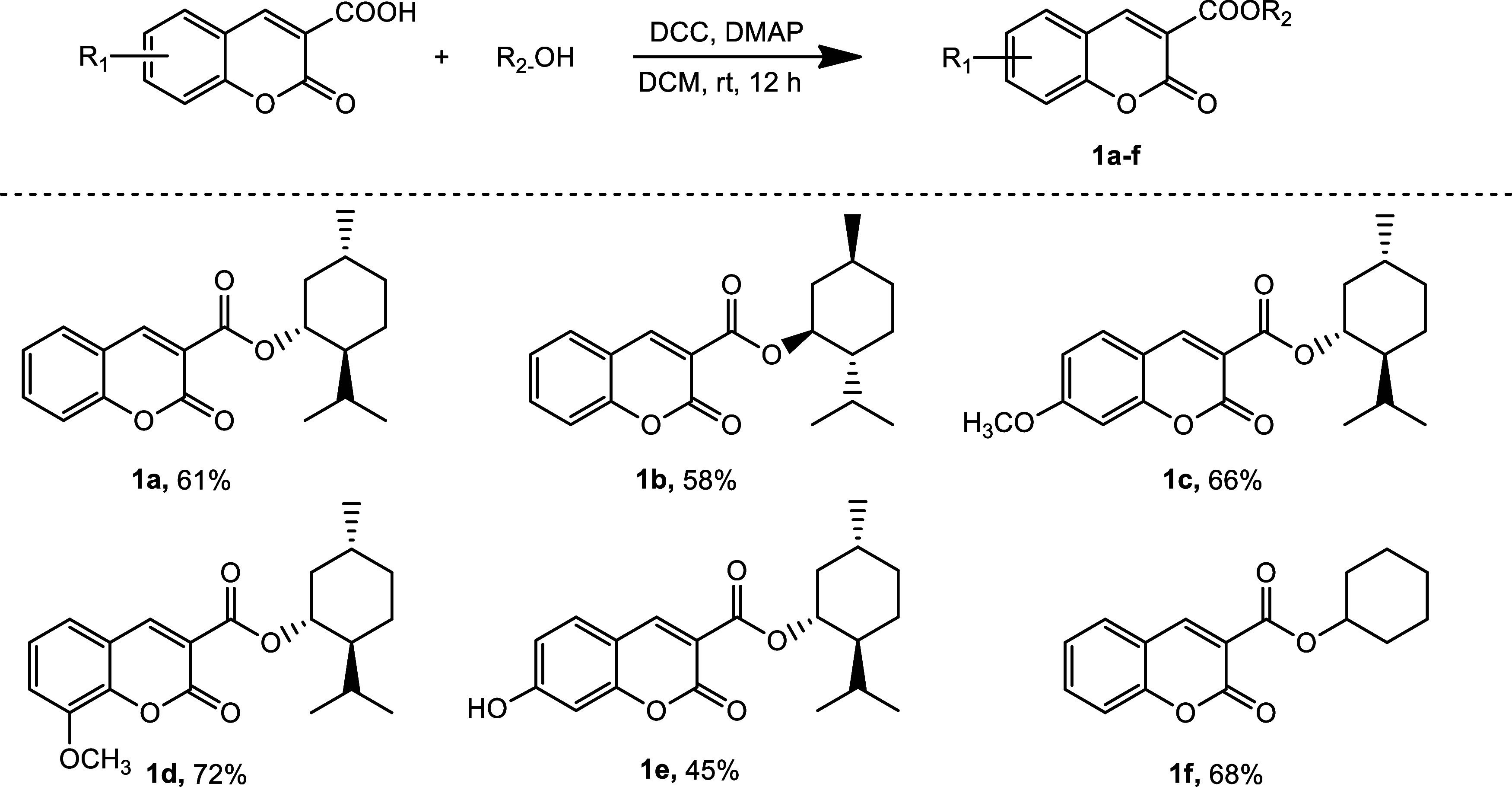
Synthesis of Coumarin Esters **1a**–**f**

The first synthetic procedures were initiated with the synthesis of coumarin-3-carboxylic acid esters (Scheme 1). Several methods for the synthesis of such compounds have been reported in the literature. One of these ways is to use reactions like (a) combining malonic acid esters with salicylaldehyde,^17^ (b) FeCl_3_-catalyzed multicomponent reactions with Meldrum’s acid, alcohol, and salicylaldehyde,^18^ (c) esterification of coumarin-3-carboxylic acid with alcohol while potassium hexafluorophosphate (KPF_6_) is present,^19^ or (d) using DCC (*N,N′*-dicyclohexylcarbodiimide) with DMAP (Steglich esterification),^20^ and (e) in reaction of coumarin-3-carboxylic acid chloride with alcohol.^21^ We recently presented the synthesis of a menthyl and cyclohexyl ester of coumarin-3-carboxylic acid (**1a** and **1f**) successfully with Steglich esterification,^12^ which we consider to provide a very versatile and readily manipulable synthetic approach. The reaction of coumarin-3-carboxylic acid derivatives with various alcohols, including cyclohexanol, l-menthol, d-menthol (2.0 equiv) in the presence of DCC (1.7 equiv) and DMAP (5.0 mol %) in methylene chloride (DCM) at rt after 12 h yielded the target coumarins **1a**–**f** in 45–72% yield (Scheme 1).

The Knoevenagel condensation reaction was employed in the synthesis of 3-phosphorylated coumarins **2a**, **2b**, and **3**, (Scheme 2). In the presence of piperidine and acetic acid, ethyl 2-(diethoxyphosphoryl)­acetate was subjected to reaction with salicyl aldehyde derivatives. We conducted the reactions in acetonitrile at 80 °C, yielding **2a** and **2b** after 18 h, with high yields of 70 and 84%, respectively. Under the appropriate conditions, the reaction of 2-(diphenylphosphoryl)­acetic acid with 3-methoxy-2-hydroxybenzaldehyde afforded the corresponding coumarin **3** in 90% yield.^14^

**Scheme 2:**
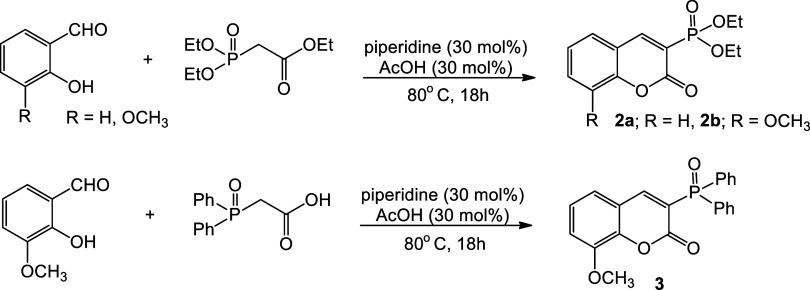
Synthesis of 3-Phosphorylated Coumarins

Biological investigations revealed that the obtained coumarin esters **1a**–**f** displayed good anticancer activity (see Section 3.2). We found this fact unsatisfactory, so we decided to modify the compounds’ structure and introduce an additional phosphoryl group to enhance the biological activity. The presence of a C=C bond in the coumarin backbone allows the introduction of a phosphoryl group through a simple phospha-Michael addition reaction.^22^ Examples of decarboxylative phosphorylation of alkenes and alkynes including coumarin-3-carboxylic acid derivative reactions are known in the literature, which proceed without the use of acids, bases, catalysts, or other additives.^23^ These reactions allow the addition of P–H bonds to alkenes in a simple and efficient route and in accordance with the green chemistry principles. Following these reports, we predicted that similar conditions would enable the addition of a secondary phosphine oxide to the structure of coumarin-3-carboxylic acid esters. To optimize the reaction conditions, we performed the first reaction between menthyl coumarin-3-carboxylate (1.0 equiv) and diphenylphosphine oxide (1.0 equiv) at room temperature without any additives in different solvents like H_2_O, EtOH, DCM, acetonitrile, and DMSO (Table S1, see Supporting Information). After 4 h, we achieved full conversion of the reaction and a yield of 90% of the product (**4a**) in acetonitrile (Scheme 3). Water was also a suitable medium for the reaction, and the water-insoluble reaction product could be obtained in 85% yield after filtration. Similarly, we obtained compounds **4b** and **4c** in 93 and 98% yields, respectively.

**Scheme 3:**
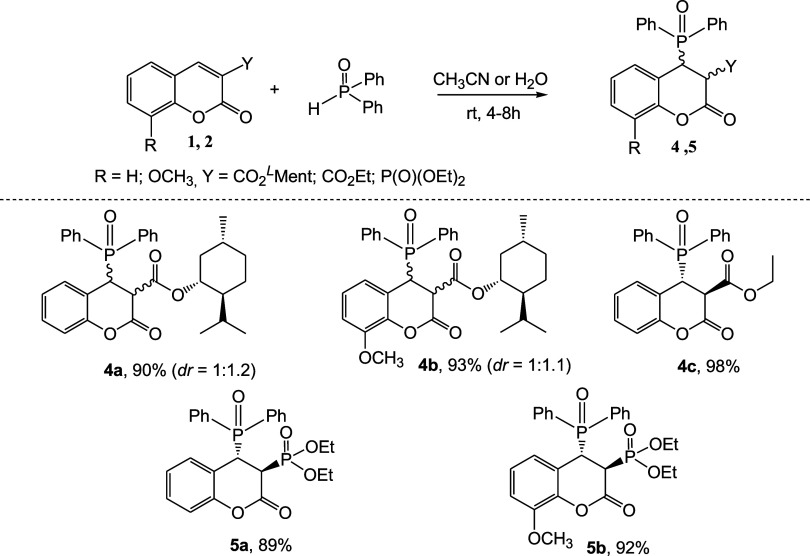
H–P­(O)­Ph_2_ Addition Reactions to Coumarins

The ^31^P NMR spectrum of crude compound **4a** revealed two distinct phosphorus signals with an integral ratio of **1:1.2**, indicating the formation of a diastereomeric mixture. In the ^1^H NMR spectrum of compound **4a**, H-3, located near the menthyl ester group, gave two distinct signals corresponding to the *cis* and *trans* diastereoisomers. The assignment of H-3 and H-4 was based on the expected deshielding effect of the menthyl ester, which shifts H-3 further downfield, and the proximity of H-4 to the P=O group, which induces slightly stronger shielding. This interpretation aligns with the chemical shift trends observed in related chromanone derivatives.^15^

The major isomer (*trans*) appeared at δ = 4.12 ppm with coupling constants of *J* = 1.26 and 9.46 Hz, while the minor isomer (*cis*) gave a signal at δ = 4.09 ppm with *J* = 0.95 and 9.72 Hz. The integral ratio of these signals was consistent with the ratio observed in the ^31^P NMR spectrum. These results were further supported by single-crystal X-ray diffraction analysis of compound **4a**, which confirmed the formation of a diastereomeric mixture composed of two stereoisomers differing in the relative configuration at positions C3 and C4 of the dihydrocoumarin ring. The crystal structure revealed the presence of both *trans* and *cis* isomers in the asymmetric unit (Figure 3). The diphenylphosphine oxide and ester substituents are on opposing sides of the ring system in the *trans* isomer (**4a**-A). In the *cis* isomer (**4a**-B), both groups are on the same side. These findings are in line with the diastereomeric result of the phospha-Michael addition. A similar finding was observed for compound **4b**, which was isolated in 93% yield, with a diastereomeric ratio of 1:1.1, as determined by ^31^P NMR spectroscopy. In the case of compounds **4a** and **4b**, multiplite attempts to separate the individual stereoisomers *(trans* and *cis*) using both recrystallization and column chromatography failed due to their similar physicochemical properties. Therefore, the biological activity data presented herein refer to these mixtures.

**Figure 3:**
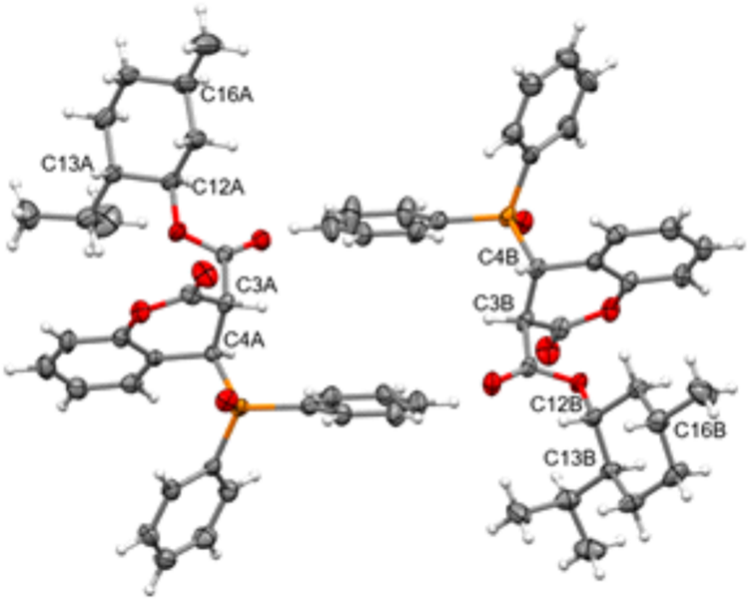
ORTEP representation of diastereomers **4a**-A (*trans*, left) and **4a**-B (*cis*, right), observed in the crystal structure. Thermal ellipsoids are shown at the 50% probability level.

Compound **4c**, on the other hand, was produced in high yield (98%) as a single *trans* diastereoisomer, based on the presence of only one set of signals in the NMR spectra. Moreover, the methodology was successfully extended to 3-phosphorylated coumarins, yielding compounds **5a** and **5b** as single *trans* isomers in 89 and 92% yields, respectively.

It is worth highlighting the simplicity and practicality of this reaction, which does not require any catalyst or additive. The key in this case is probably the architecture of the substrate, i.e., the presence of a C=O or P=O group, which enables the Michael addition reaction. The reaction proceeds in a short time under mild temperature conditions and environmentally friendly solvents such as CH_3_CN or water and is not oxygen-sensitive. In addition, the reaction products do not require purification by column chromatography, just evaporation of the solvent and, if necessary, crystallization. In the forthcoming publication, we will outline the utility and limitations of our new procedure.

The introduction of the phosphonate group into the coumarin skeleton in an analogous procedure was unsuccessful. To obtain compound **4d**, it was necessary to add DABCO in an amount of 20 mol % (Scheme 4). After 12 h at room temperature, the compound yielded 78%.

**Scheme 4:**
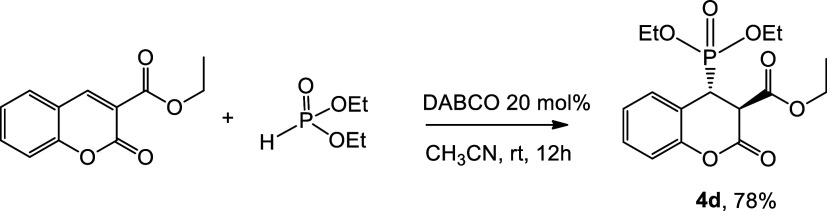
Coupling Reaction of Ethyl Coumarin-3-carboxylate and H–P­(O)­(OEt)_2_

### 3.2 Biological Studies

#### 3.2.1 Cytotoxic Activity

Cell cultures originating from human cancer are the predominant models employed to examine the *in vitro* efficacy of new chemicals as potential therapeutic candidates. To estimate antitumor properties of synthesized compounds **1a**-**1f**, **2a**, **2b**, **3**, **4a**–**4d**, **5a**–**5b**, *in vitro* cytotoxic studies has been carried out on human colon (SW480, SW620) and prostate (PC3) cancer cell lines, breast adenocarcinoma (MDA-MB-231) and human keratinocytes (HaCaT), by the MTT method (Table 1). Doxorubicin and cisplatin were used as reference chemotherapeutics. As established, both metastatic SW620 and PC3 cells turned out to be the most sensitive to the presence of selected derivatives, whereas MDA-MB-231 cell line was the most resistant. Three derivatives (**2b**, **4a**, **4b**) were active against cancer cells at doses lower than 10 μM, more efficient than cisplatin. The dihydrocoumarin derivative **4a** proved to be the strongest cytotoxic agent for the entire panel of tested cells. Its IC_50_ values toward SW480 and PC3 cell lines varied from 4.6 to 9.8 μM, being 1.3–2.3 lower than these of cisplatin. That compound, with IC_50_ of 6.8 μM for SW620 cell line, was equipotent to the reference drug. The calculated selectivity indexes (SI) over normal cells ranged from 1.53 to 3.28. The structurally related derivative **4b** inhibited the growth of PC3 cells at concentration of 9.9 μM, with satisfactory selectivity (2.78), as compared to the references. As one, its potency against other studied cancer lines was visibly weaker (IC_50_ above 19.8 μM). On the other hand, the phosphonate **2b** exhibited high cytotoxicity against SW620 cell line at 10.2 μM, with excellent SI (9.8) over other tested cell lines. Besides, close structural analogs **1d** and **1e** were similarly moderately active against SW480 (25.4–27.7 μM) and PC3 (29.3–21.8 μM) cell lines. Other compounds of the series (**1a**–**1c**, **1f**, **3**, **4c**, **4d**, **5a**, **5b**), at doses exceeding 36.6 μM, exerted weak cytotoxic activity. The human breast adenocarcinoma appeared to be vulnerable to short-term contact with studied compounds, only when the concentration used was above 25.3 μM. Considering obtained results, the most promising derivatives **2b**, **4a**, **4b** were selected for further studies of the mechanisms of activity.

**Table 1:**
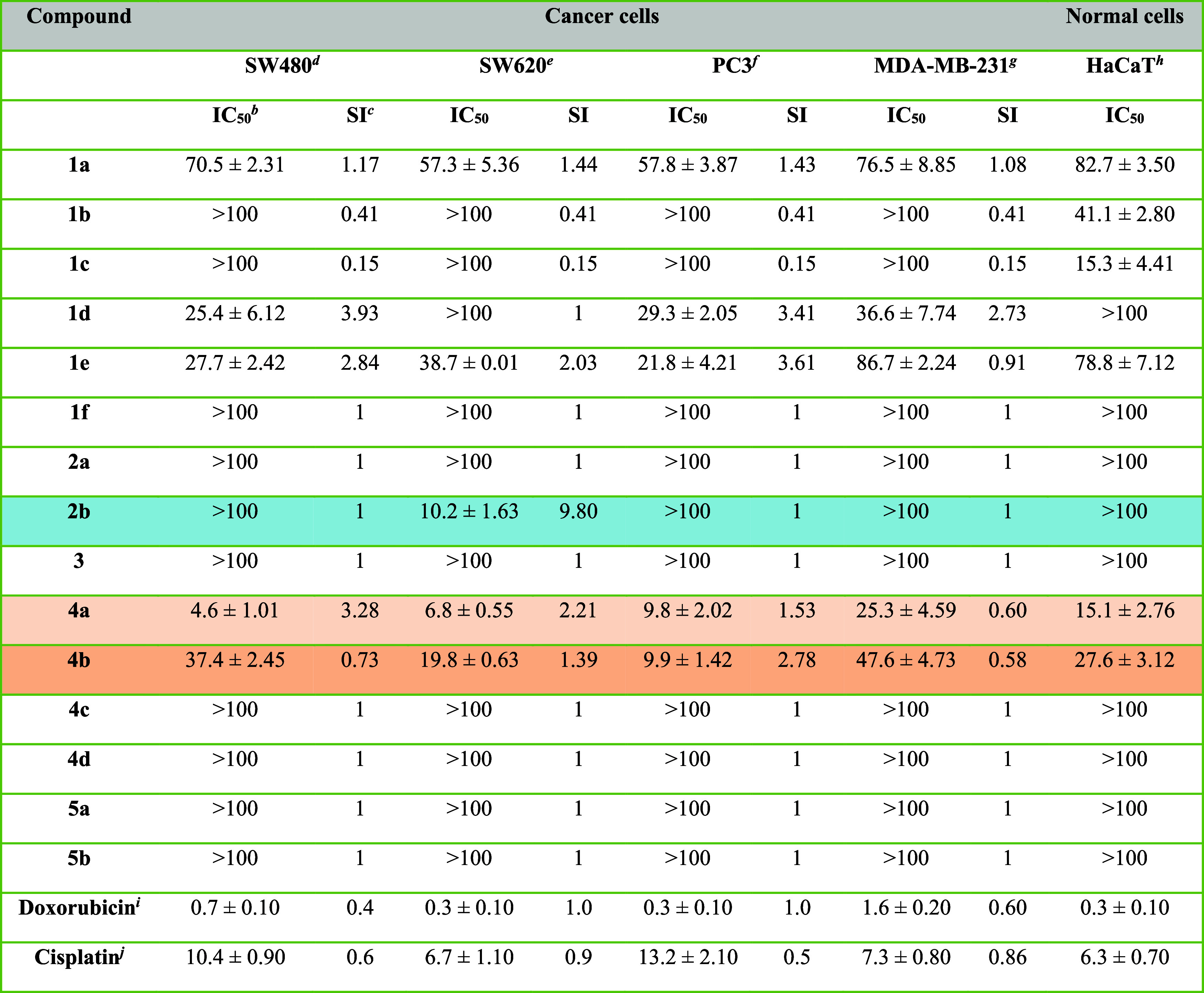
Cytotoxic Activity (IC_50_, μM) of Studied Compounds Estimated by the MTT Assay^a^

a Data are expressed as mean SD.

b IC_50_ (μM)—the half-maximal growth inhibitory concentration after cultured the cells for 72 h with the individual compound.

c The SI (Selectivity Index) was calculated using formula: SI = IC_50_ for normal cell line/IC_50_ cancer cell line.

d Human primary colon cancer (SW480).

e Human metastatic colon cancer (SW620).

f Human metastatic prostate cancer (PC3).

g Human breast adenocarcinoma (MDA-MB-231).

h Human immortal keratinocyte cell line from adult human skin (HaCaT).

i,j The reference compounds.

#### 3.2.2 Antiproliferative Activity

In order to get information about the total number of cells and the rate of viable cells in studied cell populations treated with compounds **2b**, **4a**, and **4b**, the trypan blue dye assay was accomplished. Although the viability of cancerous cells incubated for 72 h with examined derivatives remained nearly unchanged, their number has been significantly reduced, in comparison with control probes (Figure 4, Table S2). These observations have proved cytostatic properties of tested substances toward mentioned types of cells. The dihydrocoumarin derivative **4a** diminished the number of live SW480 and PC3 cells by 79.9 and 62.1%, respectively. The strong decline in SW620 cells population in the presence of this compound, accounted for 47.6%, was also denoted. In addition, the derivative **2b** inhibited the growth and proliferation of SW620 cells, reducing their amount by 36.4%. Similarly, the population of PC3 cells was lowered by 35.3%, when incubated with the compound **4b**, comparing to controls. As expected, human HaCaT cell line was less sensitive to target derivatives. Tested compounds, administrated at their IC_50_, did not affect normal cells viability. The reducing influence of the phosphonate **2b** on the number of human keratinocytes was weak (below 10%), whereas the impact of the dihydrocoumarin **4b**-only moderate (equaled 16.6%). The most bioactive compound **4a** reduced the number of HaCaT cells by 31.9%, however it is at least twice as selective compared to the effect on tumor cells, and several times less cytotoxic than reference chemotherapeutics.

**Figure 4:**
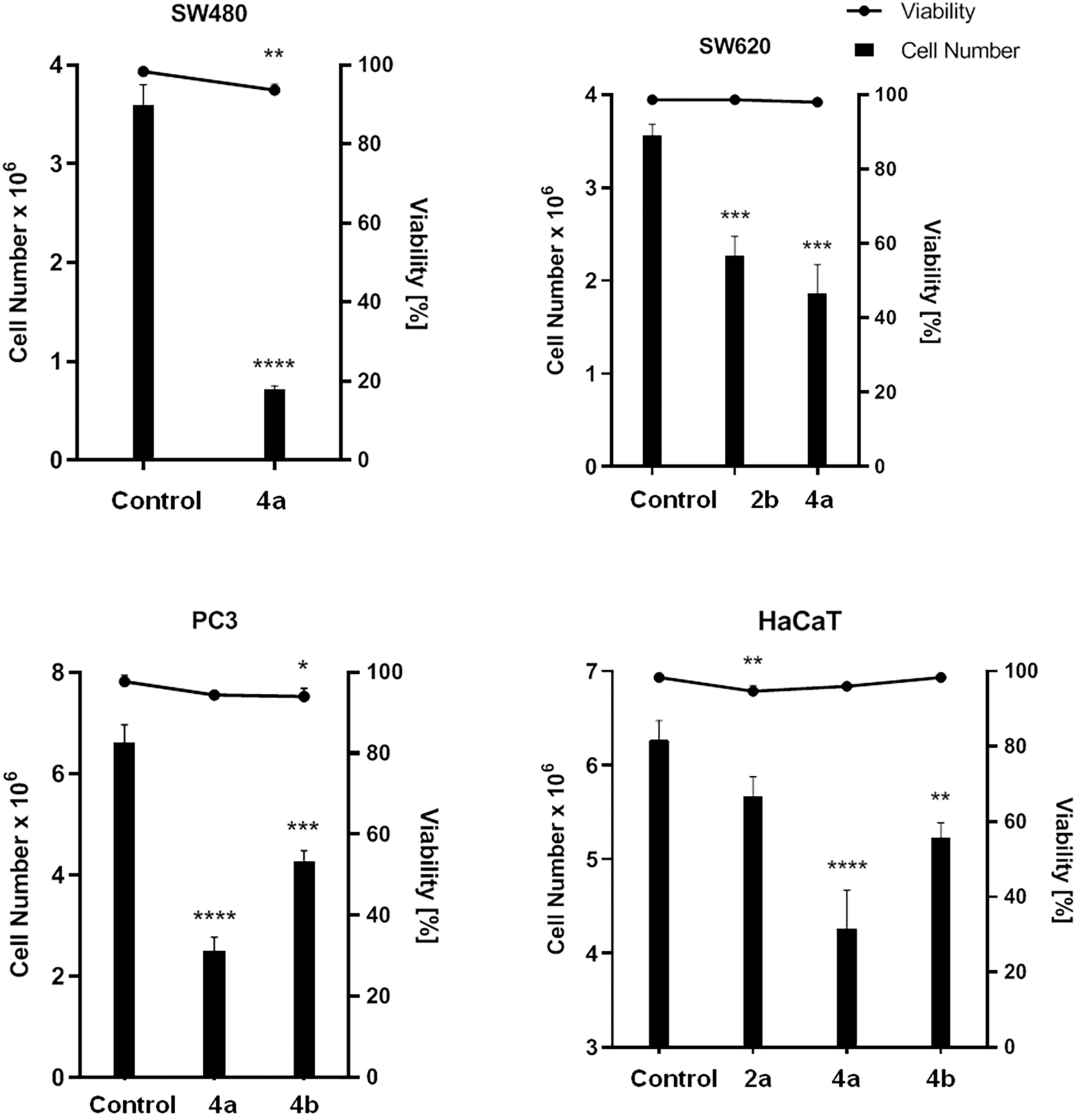
Effect of selected compounds (**2b, 4a, 4b**) on the live cell number and viability (%), measured by trypan blue assay. Cells were treated with studied compounds at their IC_50_ for 72 h. Data are expressed as the mean ± SD ****p ≤ 0.0001; ***p ≤ 0.001; **p ≤ 0.01; *p ≤ 0.05, as compared to the control.

#### 3.2.3 Apoptotic Activity

Apoptosis, one of the natural mechanisms of the cell death, is a hopeful target for cancer treatment. Thus, as the next stage of studies of activity mechanisms, the ability of the most cytostatic compounds **2b**, **4a**, and **4b** to trigger the cell death by apoptosis or necrosis was evaluated, using flow cytometry analysis (Figure 5).

**Figure 5:**
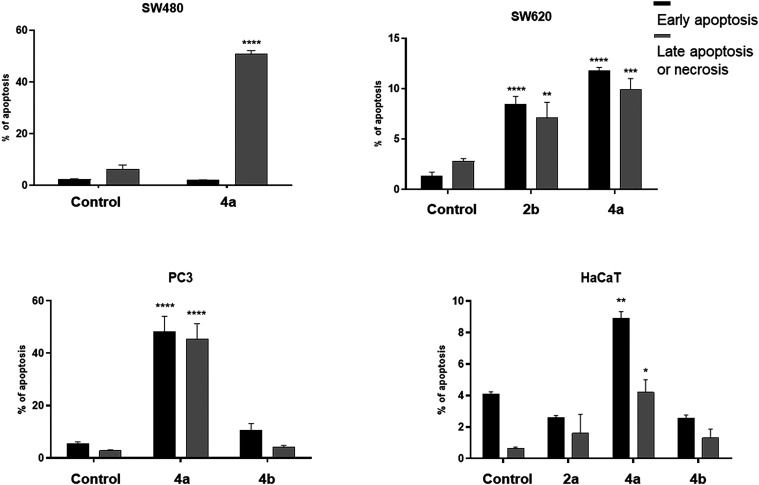
Effect of compound **2b**, **4a**, and **4b** on early and late apoptosis or necrosis in SW480, SW620, PC3, and HaCaT cells. Cells were incubated for 72 h with the tested compounds used in their IC_50_ concentrations, then cells were harvested, stained with Annexin V-FITC and PI, and analyzed using flow cytometry. Data are expressed as % of cells in the early stage of apoptosis, and as % of cells in the late stage of apoptosis. Data are expressed as the mean ± SD ****p ≤ 0.0001; ***p ≤ 0.001; **p ≤ 0.01; *p ≤ 0.05 as compared to the control.

As observed, new derivatives, applied in their IC_50_ doses, revealed early and/or late apoptosis-inducing properties in studied cancer cell lines (Figure 6, Table S3). The molecule **4a** showed a high percentage of PC3 cells in both early (48.30%) and late (44.75%) apoptosis, comparing to untreated cells. It influenced also pro-apoptotic processes in SW480 cells, inducing late apoptosis in 48.95% of cell population. Unexpectedly, that derivative **4a** has not activated remarkably cellular mechanisms leading to apoptotic/necrotic cell death in metastatic SW620 cells. These observations were registered only for 11.75% (early apoptosis) and 10.25% (both late apoptosis and necrosis) of the above-mentioned type of cancer cells. Similarly, less than 10% of SW620 cells entered early and late apoptosis in the presence of phosphonate derivative **2b**. The percentage of PC3 cells in early apoptosis after incubation with the product **4b** equaled 10.50%. None of the derivatives promoted necrosis in studied tumor cells, as compared to controls. However, the impact of synthesized compounds on cell death processes in human normal HaCaT cells was negligible, including also the most cytostatic compound **4a**. The observed percentage of keratinocytes in apoptosis and necrosis did not exceed 8.90 and 0.45% of total number of cells, respectively, comparing with control experiments. The obtained data comply with the IC_50_ values of the lead dihydrocoumarin **4a**, found for selected cancer cell lines.

**Figure 6:**
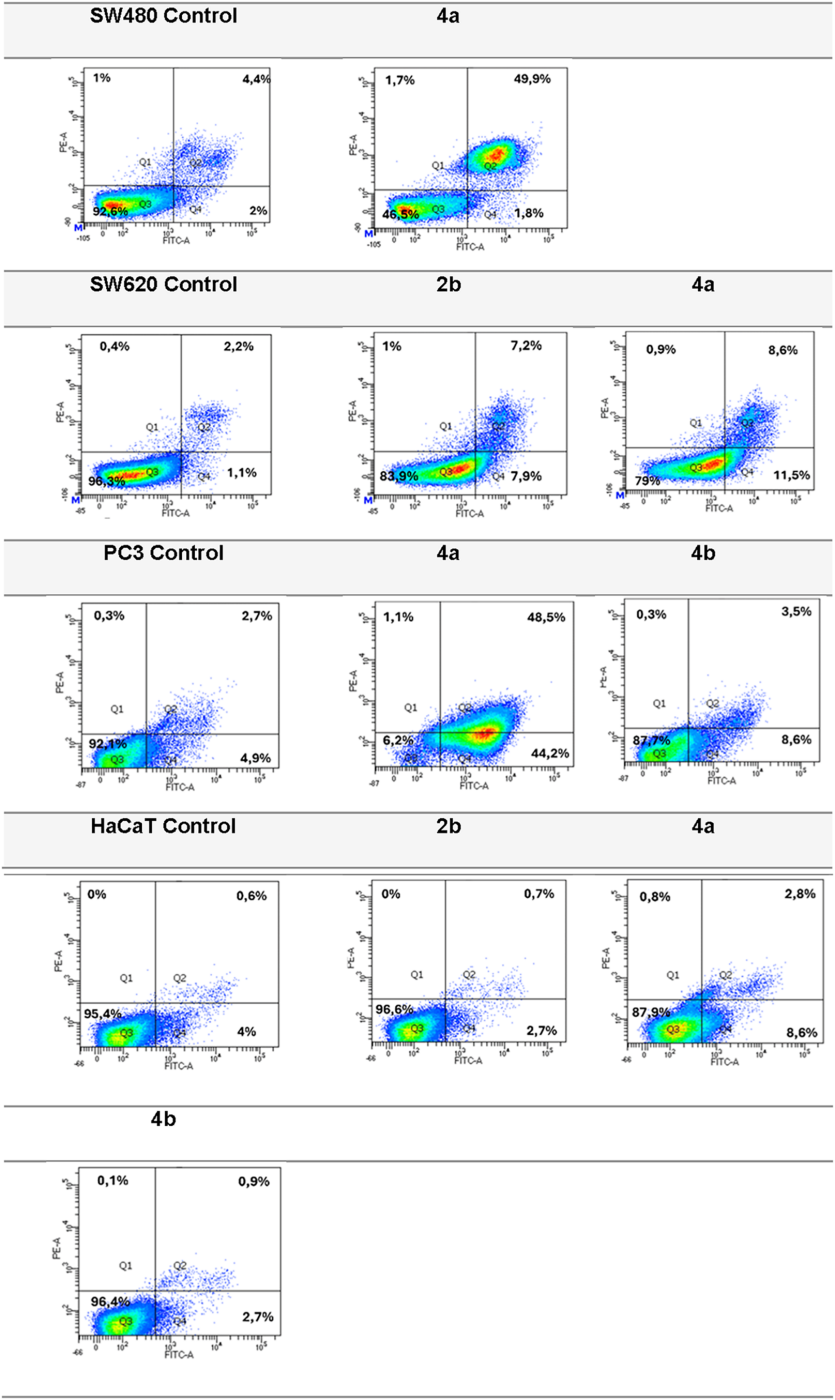
Representative results (%) as dot plots from apoptosis analysis of SW480, SW620, PC3, and HaCaT cells after treatment with compound **2b**, **4a**, and **4b** detected with Annexin V-FITC/PI by flow cytometry. Q1—necrotic cells, Q2—late apoptotic cells, Q3—live cells, Q4 - early apoptotic cells.

#### 3.2.4 Inhibition of IL-6 Release

A multifunctional interleukin-6 (IL-6) is a cytokine implicated in many inflammatory and autoimmune disorders.^24^ It is also considered as a factor promoting tumor growth, such as prostate, colon, renal or ovarian cancer, as well as lymphoma and glioma.^25^ Increased expression of IL-6 in tumor cells can be related to cancer growth and aggressiveness, as this cytokine promotes cell proliferation, angiogenesis and metastasis.^26^ In the search for substances able to attenuate cytokine secretion, IL-6 concentrations in prostate and both colorectal cancer cell lines were measured in the presence of IC_50_ of compounds **2b**, **4a**, and **4b** (Figure 7, Table S4). Notable inhibitory effects were found for all derivatives and cell types. The dihydrocoumarin **4a** reduced the IL-6 level in PC3, SW480 and SW620 cells by 87.2, 77.6, and 69.1%, respectively. The derivative **4b** expressed significant potency in PC3 cells, achieving an 79.2% decrease in interleukin concentration, comparing to control experiments. On the other hand, the phosphonate **2b** inhibited the cytokine release in SW620 cells with less strength (by 33.5%), in comparison with other studied analogs. Considering compounds influence on normal keratinocytes, the derivative **4b** did not alter IL-6 secretion, while the incubation of HaCaT cells with the coumarin **2b** resulted in a 10.7% increase in interleukin level. Although the most potent compound **4a** diminished the IL-6 concentration in normal cells by 26.5%, tested cancer cell lines were 2.6–3.3 more susceptible to the short-term contact with this agent.

**Figure 7:**
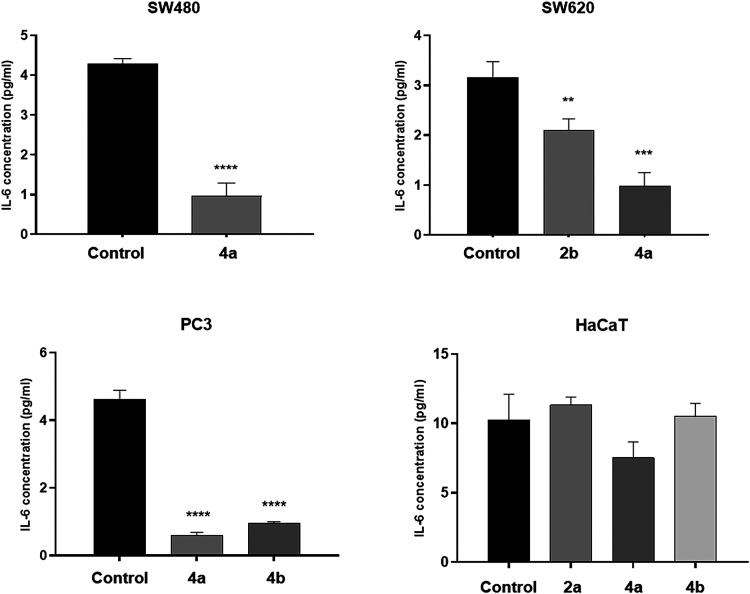
Effects of compounds **2b**, **4a**, and **4b** on IL-6 levels, measured by the ELISA test. Data are expressed as the mean ± SD from primary colon cancer (SW480), metastatic colon cancer (SW620), metastatic prostate cancer (PC3) and immortal keratinocyte cell line from adult human skin (HaCaT). ****p ≤ 0.0001; ***p ≤ 0.001; **p ≤ 0.01; *p ≤ 0.05 as compared to the control. ELISA—enzyme-linked immunosorbent assay; IL-6—interleukin.

#### 3.2.5 *In Vivo* Toxicity Assessment Using *Danio rerio* Larvae

The zebrafish (*Danio rerio*) used as an embryonic and larval model for conducting *in vivo* investigations and developmental toxicity assessments. Zebrafish can be employed to assess the toxicity of materials in preliminary screening experiments.^27^

Coumarin (2*H*-chromen-2-one), coumarin menthyl ester (**1a**), phosphorylated coumarin (**2b**), and 3,4-dihydrocoumarin (**4a**) were selected for in vivo cytotoxicity studies. The maximum tolerated concentration (MTC) was calculated to assess the toxicity of the tested compounds. *In vivo* studies using zebrafish (120 hpf) demonstrated that coumarin and 3-phosphorylated coumarin **2b** exhibited lower toxicity compared to **1a** and **4a**. The highest MTC of 100 μM was observed for both coumarin and compound **2b**. The MTC value for dihydrocoumarin **4a** was 75 μM, slightly lower than those of coumarin and compound **2b**. In contrast, the MTC for compound **1a** was 10 times lower than that of coumarin or compound **2b** (MTC = 0 μM). Thus, compound **2b** showed low toxicity in *in vivo* studies on zebrafish larvae.

Coumarin **2b** also displayed low cytotoxicity in *in vitro* studies, inhibiting the growth of SW620 cells only at a concentration of 10.2 μM. Thus, it exhibited a selective effect on metastatic colon cancer cells. Compared to **1a** and **4a**, compound **2b** did not show any cytotoxic effects on healthy cells (HaCaT), which is consistent with the *in vivo* MTC value of 100 μM. Dihydrocoumarin **4a**, on the other hand, demonstrated significant inhibition of colon cancer and human prostate cancer cell growth, as well as high cytotoxicity toward healthy cells and greater *in vivo* toxicity compared to coumarin and **2b**. Compound **1a** showed 10-fold higher toxicity in *in vivo* studies compared to coumarin and **2b**. However, *in vitro* tests revealed that it did not exhibit anticancer properties.

### 3.3 *In Silico* Prediction of Physicochemical and ADME Properties

Ensuring control over the physicochemical parameters of compounds at the initial stages of drug discovery and active substance optimization is crucial for steering the molecular structure of new candidates toward desired pharmacokinetic properties, facilitating subsequent in vivo testing. To determine if our compounds exhibit suitable physicochemical, ADME, and toxicity characteristics, we performed an *in silico* evaluation using the SwissADME online platform^28^ with the results summarized in Tables S7–S9 (see Supporting Information).

Given that anticancer activity was demonstrated against tested cancer cell lines only by compounds **4a**, **4b**, **1e**, **1d**, **1a**, **2b** with average IC_50_ values of 11.62, 28.67, 43.72. 47.82 47.82, 65.52, 77.55 μM, respectively, further analysis was confined to these compounds. Log *P* (partition coefficient) is a commonly used descriptor of a compound’s lipophilicity, defined as the logarithm of the ratio of its concentrations in octanol and water at equilibrium. It reflects the compound’s potential to permeate biological membranes and is often correlated with its absorption and distribution properties. An analysis of the physicochemical parameters (Table S5), reveals a trend, with the exception of compound **1e**, suggesting that increased lipophilicity correlates positively with enhanced bioactivity, independent of the algorithm used to calculate Log *P*. The two most active derivatives **4a** and **4b** which contain a bulky oxodiphenylphosphonium group exhibit the highest lipophilicity with average Log *P* values of 5.54 and 5.51, respectively. Moreover, a statistically significant negative correlation was observed between compound activity and both the number of heavy atoms (or molecular weight) as well as the molar refractive index.

*In silico* ADME profiling results (Table S8 and “BOILLED egg” diagram in Table S9) predict that all active compounds should exhibit high oral absorption potential. However, only coumarins **1a** and **1d**, characterized by a favorable balance of lipophilicity (WLOGP, an algorithm developed by Wildman and Crippen for estimating Log *P* values based on molecular fragments) and topological polar surface area (TPSA), are likely to penetrate the blood-brain barrier.^29^ Radar diagram of bioavailability (Table S9) indicating the influence of lipophilicity, molecular weight, polarity, solubility, and shape descriptors, suggests that minor structural modifications aimed at slightly reducing lipophilicity and enhancing solubility could improve the absorption of highly active dihydrocoumarins **4a** and **4b**. However, compounds **4a** and **4b** are predicted to be substrates for P-glycoprotein, a major ATP-binding cassette transporter, potentially impacting gastrointestinal absorption, increasing central nervous system efflux, and rendering them prone to multidrug resistance. SwissADME’s support vector machine-based predictions also indicate that compounds **4a**, **4b**, and **1d** are likely inhibitors of CYP3A4, the principal cytochrome P450 isoform involved in drug metabolism. The second crucial isoform CYP2D6 responsible for metabolizing approximately 20–25% of drugs^30^ is predicted to be inhibited solely by compound **4b**.

Analysis of the most frequently used bioavailability and drug-likeness molecular filters^31^ shows that none of the compounds violate Veber’s criteria. On the other hand again compounds **4a** and **4b** violate the Lipinski’s (2 violations), Ghose’s (4 violations) and Egan’s (1 violation) rules (Table S7). However, using the above filters to reject certain compounds must be done with caution especially if compounds already proven to be active. Expanding beyond the conventional rule-of-five framework like in a case of compound **4a** and **4b** may uncover promising active chemotypes otherwise overlooked.^32^

### 3.4 Structure–Activity Relationship

Numerous review publications have highlighted the biological activity of coumarin, but modification of its architecture is necessary to fully exploit this potential.^1–9^ The C-3 and C-4 positions were found to be more significant in achieving appropriate biological efficacy.

Of the series of compounds **1a**–**e**, coumarin bearing an OCH_3_ group at the C-8 position exhibited the highest activity against 3 cancer cell lines (IC_50_ 25.4–36.6 μM) remaining low in toxicity to normal cells (Figure 8). The presence of an l-menthyl moiety was significant; compound **1b** with a d-menthyl group and **1f** with a cyclohexyl group lacked activity.

**Figure 8:**
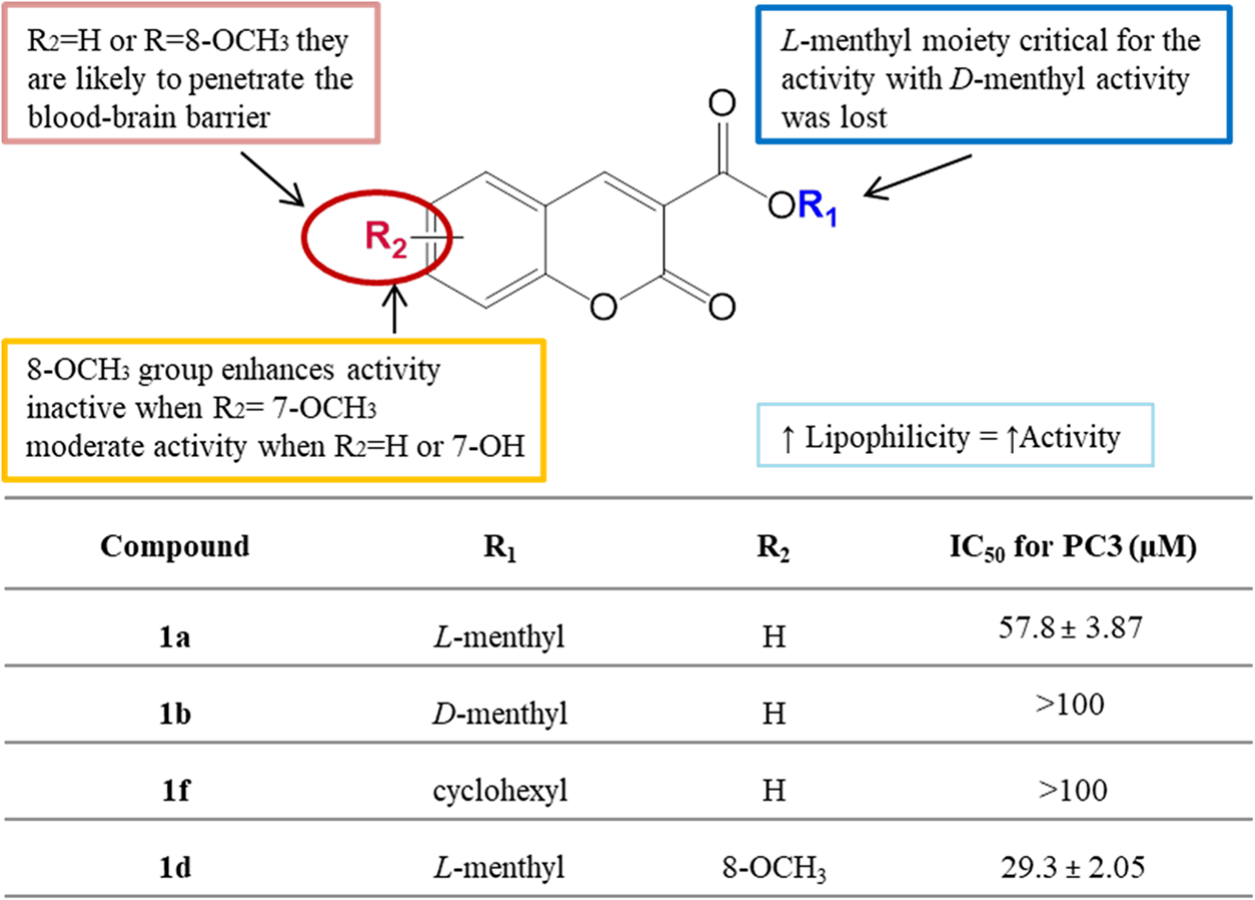
SAR study of coumarin ester **1**.

The introduction of a phosphonate group at the C-3 position of coumarin and the presence of the 8-OCH_3_ group yielded compound **2b**, which is active against human metastatic colon cancer (SW620, IC_50_ 10.2 μM).

A very promising modification of the coumarin backbone appeared to be the introduction of a diphenylphosphine oxide group into the coumarin-3-carboxylic acid menthyl esters. The resulting dihydrocoumarin derivatives **4a** and **4b**, applied as inseparable mixtures of *cis* and *trans* diastereomers, demonstrated excellent cytotoxicity on PC3 cancer cells (IC_50_ 9.8 and 9.9 μM, respectively), Figure 9. These derivatives combine high lipophilicity (Log *P* 5.54 and 5.51) due to the oxodiphenylphosphoryl moiety and menthyl ester, which likely facilitates membrane penetration and enhances biological activity.

**Figure 9:**
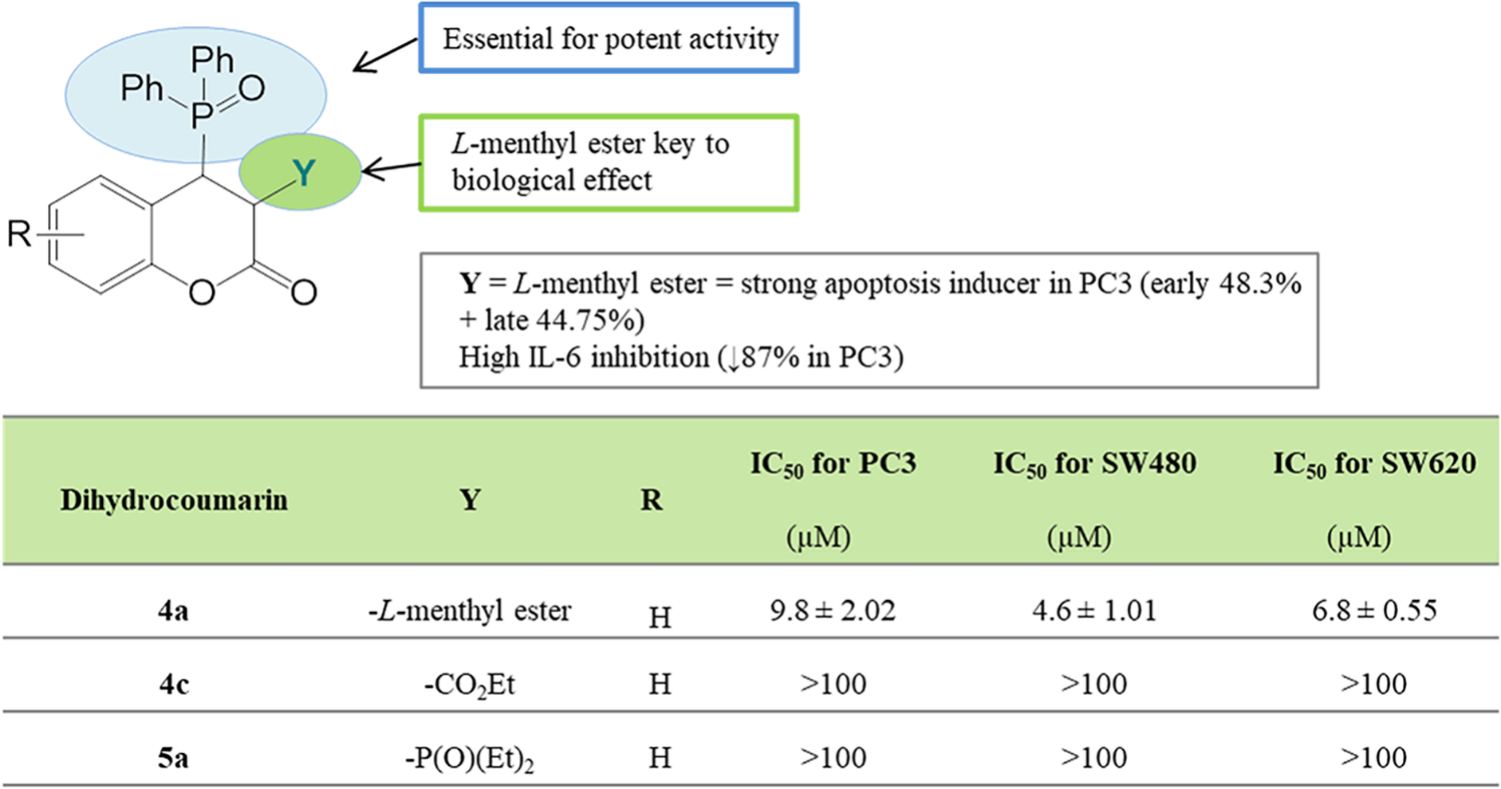
SAR study of 3,4-dihydrocoumarins.

The function of coumarin derivatives in regulating important biological pathways, such as inflammation and apoptosis, has been highlighted in recent research. Shaik and colleagues highlighted that apoptosis induction and IL-6 suppression are common mechanisms of anticancer activity for this class of compounds.^33^ These observations are consistent with our results: compound **4a**, the strongest derivative, caused both early (48.3%) and late (44.8%) apoptosis in PC3 cells in addition to reducing IL-6 levels by 83%. These results suggest that a dual anti-inflammatory and pro-apoptotic mechanism underpins its cytotoxic action.

Further structural modifications of the coumarin scaffold revealed that substitution of the menthyl group with an ethyl or diethylphosphate moiety (**4d**) resulted in loss of antitumor activity (Figure 10), especially noticeable in assays performed on PC3 prostate cancer cells.

**Figure 10:**
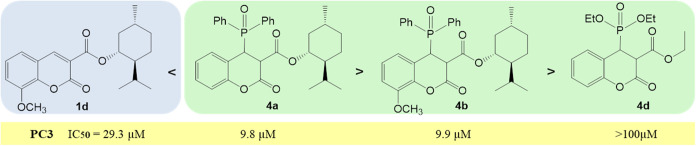
Cytotoxicity of compounds **1** and **4** on PC3 cancer cells.

## 4 Conclusion

The efficient synthesis of coumarin esters bearing a menthyl group (**1a**–**e**) and 3-phosphorylated coumarin derivatives (**2a**, **2b**, **3**) was achieved. The -P­(O)­Ph_2_ group was subsequently introduced into the structure of these compounds at the C-4 position, thus obtaining 3,4-dihydrocoumarin derivatives. This approach was intended to investigate the synergy of the coumarin backbone, the menthyl group, and the phosphoryl fragments. The synthesis of 3,4-dihydrocoumarins employed a simple Michael addition reaction, which was performed without catalysts or additives, obtaining high yields and simplicity of the procedure. The title compounds were thoroughly characterized by spectroscopic methods and then subjected to anticancer evaluation against four cancer cell lines (SW480, SW620, PC3, MDA-MB-231). Compounds **2b**, **4a**, and **4b** demonstrated significant anticancer activity against the tested cell lines and displayed good selectivity toward normal cells (HaCaT). The introduction of a phosphoryl group significantly improved the cytotoxic activity and selectivity of the compounds, especially against prostate and colon cancer cells. The introduction of a phosphoryl group significantly improved the cytotoxic activity and selectivity of the compounds, especially against prostate and colon cancer cells. Compounds **4a** and **4b** were obtained as mixtures of diastereomers *cis/trans*; the biological activity data discussed herein refer to these mixtures. The current work stage could not verify possible synergistic or antagonistic effects between the stereoisomers. We believe that using a mixture of isomers for biological testing is an appropriate strategy for the early stages of drug development. We aimed to evaluate this novel class of compounds’ biological potential in a preliminary manner. In early drug discovery, it is common practice to begin biological evaluations with racemic or diastereomeric mixtures, particularly when those mixtures show promising cytotoxic effects. Diastereomeric or racemic medications are not always less safe or effective than their pure stereoisomeric counterparts, as recent research has discussed.^33^ Furthermore, until a pertinent differentiated property warrants a chiral switch, the development of chiral drugs should continue with the mixture (racemate or diastereomeric), according to Agranat and D’Acquarica.^34^

Investigation of the mechanisms of action revealed that the selected compounds (**2b**, **4a**, and **4b**) induce apoptosis, inhibit tumor cell proliferation, and reduce interleukin-6 (IL-6) secretion. Compound **2b** appears to be promising, as it selectively inhibits the growth of metastatic colon cancer cells (IC_50_ = 10.2 μM) while exhibiting no toxic effects *in vitro* (IC_50_ > 100 μM) and *in vivo* (MTC = 100 μM). *In silico* analysis confirmed the favorable physicochemical properties, ADME, and pharmacokinetic profiles of the selected compounds, indicating their potential as candidates for further biological studies. The results presented in the publication allowed us to identify key structural elements determining the biological activity of molecules based on the coumarin skeleton. This preliminary but promising activity of the compounds provides us with a convincing justification for further research, such as exploration of the mechanism of action, separation of stereoisomers, and more detailed biological evaluation.

## Supplementary Material



## Notes

NMR spectra, MS chromatograms for newly synthesized compounds (1c–1e, 4a–4c, and 5a–5b), Table S1 with optimization of reaction conditions for the synthesis of **4a**, results from biological studies (Tables S2–S4), X-ray experimental studies (Tables S5–S6), and *in silico* prediction and ADME properties (Tables S7–S9) (PDF)
