# Supplementary material for: Design, Synthesis, Structure–Activity Relationships, and Preliminary Anticancer Properties of Menthol-Modified Coumarin Esters and 3,4-Dihydrocoumarin Derivatives
Source: ACS Omega. 2025 Oct 1;10(40):46418–34. doi: 10.1021/acsomega.5c01784 (PMC12529164; doi:10.1021/acsomega.5c01784)
Supplement: Supplementary file 1 [file ao5c01784_si_001.pdf]

Supporting Information

Design, synthesis, structure-activity relationships, and preliminary anticancer properties of menthol-modified coumarin esters and 3,4-dihydrocoumarin derivatives

Katarzyna Szwaczko[a]* Paulina Strzyga-Łach[b], Marta Struga[b], Ewelina Kiernozek-Kalińska[c], Krzysztof Szafrański[d], Adrianna Skiba[e], Anita Płazińska[f], Krystyna Skalicka-Woźniak[e] and Anna Bielenica[b]

[a]Department of Organic Chemistry and Crystallochemistry, Institute of Chemical Sciences, Faculty of Chemistry, Marie Curie-Skłodowska University in Lublin, 20-614 Lublin, Poland; [b]Chair and Department of Biochemistry, Medical University of Warsaw, 02-097 Warsaw, Poland; [c]Department of Immunology, Faculty of Biology, University of Warsaw, 02-096 Warsaw, Poland; [d]Department of Organic Chemistry, Faculty of Pharmacy, Medical University of Gdańsk, 80-416 Gdańsk, Poland; [e]Department of Natural Products Chemistry, Faculty of Pharmacy, Medical University of Lublin, 20-093 Lublin, Poland; [f]Department of Biopharmacy, Medical University of Lublin, ul. Chodzki 4a, 20-093 Lublin, Poland

Table of Contents

1. Chemistry-general information S3
1.2 H-P(O)Ph_2 addition to the coumarin 1a and optimization of the reaction S3
2. Biological studies S4
2.1 Table S2 S4
2.2 Table S3 S5
2.3 Table S4 S6
2.4 Fig. S1. Dose–response curves for IC_{50} determination S6
3. X-ray crystallography S9
4. In silico prediction and ADME properties S17
5. NMR spectra S21
6. HRMS Spectra of Compounds 1c–1e, 4a–4c, and 5a–5b S43
7. References S47

1. Chemistry-general information

All commercially available chemicals and solvents were acquired in good quality and utilized without additional purification. NMR spectra were recorded using a Bruker AV500 (^{1}H 500 MHz, ^{13}C 126 MHz, ^{31}P 202 MHz) spectrometer. All spectra were obtained in CDCl3 solutions, and the chemical shifts (δ) were expressed in ppm using internal reference to TMS. Coupling constants (J) were given in Hz. The abbreviations of signal patterns were as follows: s, singlet; d, doublet; t, triplet; q, quartet; m, multiplet; b, broad. Optical rotations were measured on a Perkin-Elmer 341LC digital polarimeter. Melting points were determined on a Buchi 510 apparatus. Thin-layer chromatography (TLC) was done on silica gel (Kieselgel 60, F254 on aluminum sheets, Merck) using UV light (254 nm). HPLC–HRMS was performed on a Shimadzu LCMS-8030 LCMS System using a reverse-phase stationary phase with water/MeCN (65:35) as an eluent, electrospray ionization (ESI), and an IT-TOF detector (Shimadzu Europa, Duisburg, Germany). All column chromatographic separations and purifications were conducted using Merck silica gel 60 (230–400 mesh).

1.1 H-P(O)Ph$_2$ addition to the coumarin 1a and optimization of the reaction

To a solution of coumarin **1a** (0.5 g) in solvent (10 ml), H-P(O)Ph$_2$ (1.0eq.) was added. The reaction mixture was stirred at room temperature for 4-8 h. Then solvent was evaporated to give the white solid of the pure product. In the case of a reaction carried out in water, the product was filtered off and the water was extracted twice with DCM.

Table S1. Optimization of reaction conditions for the synthesis of **4a**[a]

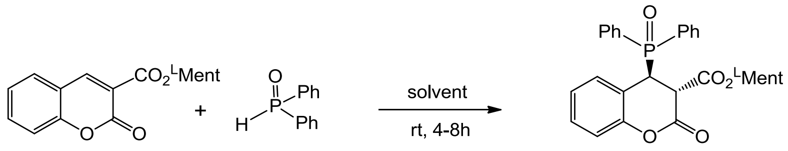

Entry	Solvent	Time [h]	Yield[b] [%]
1	CH_3CN	4	90
2	CH_3CN	8	91
3	H_2O	4	80
4	H_2O	8	85[c]
5	EtOH	4	83[c]
6	DMSO	4	86
7	DCM	4	87

[a] Reaction conditions: coumarin (**1a,** 0.5 g, 1.5 mmol), diphenylphosphine oxide (0.307 g, 1.5 mmol), solvent (10 ml), rt, time 4-8 h; [b] Isolated yields; [c] OH-P(O)Ph$_2$ was observed in the reaction mixture.

2. Biological studies

2.1 Table S2

Table S2. The effect of selected compounds (**2b, 4a, 4b**) on the live SW480, SW620 and PC3 cells number and viability (%), measured by trypan blue assay. Cells were treated with studied compounds at their IC50 for 72 h. Data are expressed as the mean ± SD.

	**viability (%)**					**cell number**				
		%				x106				
	SW480			mean	standard deviation				mean	standard deviation
**Control**	99	98	98	98.33	0.57	3.8	3.4	3.6	3.6	0.2
**4a**	92	94	95	93.66	1.52	0.75	0.73	0.69	0.72	0.03
	SW620			mean	standard deviation				mean	standard deviation
**Control**	99	98	99	98.66	0.57	3.7	3.5	3.5	3.56	0.11
**2b**	99	99	98	98.66	0.57	2.2	2.1	2.5	2.26	0.20
**4a**	99	97	98	98	1	1.6	2.2	1.8	1.86	0.30
	PC3									
				mean	standard deviation				mean	standard deviation
**Control**	98	99	96	97.66	1.52	6.9	6.7	6.2	6.6	0.36
**4a**	94	95	94	94.33	0.57	2.6	2.2	2.7	2.5	0.26
**4b**	96	94	92	94	2	4.5	4.2	4.1	4.26	0.20
	HaCaT			mean	standard deviation				mean	standard deviation
**Control**	99	98	98	98.33	0.57	6.1	6.2	6.5	6.26	0.20
**2b**	95	96	93	94.66	1.52	5.5	5.6	5.9	5.66	0.20
**4a**	97	96	95	96	1	4.7	4.2	3.9	4.26	0.40
**4b**	99	99	97	98.33	1.15	5.4	5.2	5.1	5.23	0.15

2.2 Table S3

Table S3. The effect of compounds **2b, 4a** and **4b** on early and late apoptosis and necrosis, analysed by flow cytometry. Cancer and normal cells were treated with studied compounds in their IC_{50} concentrations for 72 h. Data are expressed as % of cells in the early stage of apoptosis, as % of cells in the late stage of apoptosis or necrosis. Data are expressed as the mean ± SD.****p ≤ 0.0001; ***p ≤ 0.001; **p ≤ 0.01; *p ≤ 0.05 as compared to the control.

		Compound	Early apoptosis [%]	SD	Late apoptosis [%]	SD	Necrosis [%]	SD
	**SW480**	**Control**	**2.25**	**0.35**	**5.40**	**1.41**	**0.85**	**0.21**
		**4a**	1.95	0.21	48.95****	1.34	1.80	0.14
**Cancer cell line**	**SW620**	**Control**	**1.35**	**0.35**	**2.35**	**0.21**	**0.45**	**0.07**
		**2b**	8.45****	0.77	6.50**	0.98	0.60	0.56
		**4a**	11.75****	0.35	8.80***	0.28	1.45	0.77
	**PC3**	**Control**	**5.4**	**0.70**	**2.50**	**0.28**	**0.25**	**0.07**
		**4a**	48.30****	5.79	44.75****	5.30	0.65	0.63
		**4b**	10.50	2.68	3.90	0.56	0.20	0.14
**Normal cell line**	**HaCaT**	**Control**	**4.10**	**0.14**	**0.65**	**0.07**	**0.00**	**0.00**
		**2b**	2.60	0.14	1.30	0.84	0.30	0.42
		**4a**	8.90**	0.42	3.40*	0.94	0.08	0.00
		**4b**	2.55	0.21	0.85	0.07	0.45	0.49

2.3 Table S4

Table S4. Effects of compounds **2b, 4a** and **4b** on IL-6 levels, measured by ELISA test. Data are expressed as the mean ± SD. [a]Human primary colon cancer (SW480), [b]Human metastatic colon cancer (SW620), [c]Human metastatic prostate cancer (PC3), [d]Human immortal keratinocyte cell line from adult human skin (HaCaT).

		Compound	IL-6 concentration (pg/ml)
**Cancer cell line**	**SW480**[a]	**Control**	4.3 ± 0.12
		**4a**	0.9 ± 0.33
	**SW620**[b]	**Control**	3.2 ± 0.31
		**2b**	2.1 ± 0.22
		**4a**	0.9 ± 0.27
	**PC3**[c]	**Control**	4.6 ± 0.26
		**4a**	0.6 ± 0.09
		**4b**	0.9 ± 0.03
**Normal cell line**	**HaCaT**[d]	**Control**	10.2 ± 1.87
		**2b**	11.3 ± 0.59
		**4a**	7.5 ± 1.13
		**4b**	10.5 ± 0.94

2.4 Fig. S1. Dose–response curves for IC_{50} determination

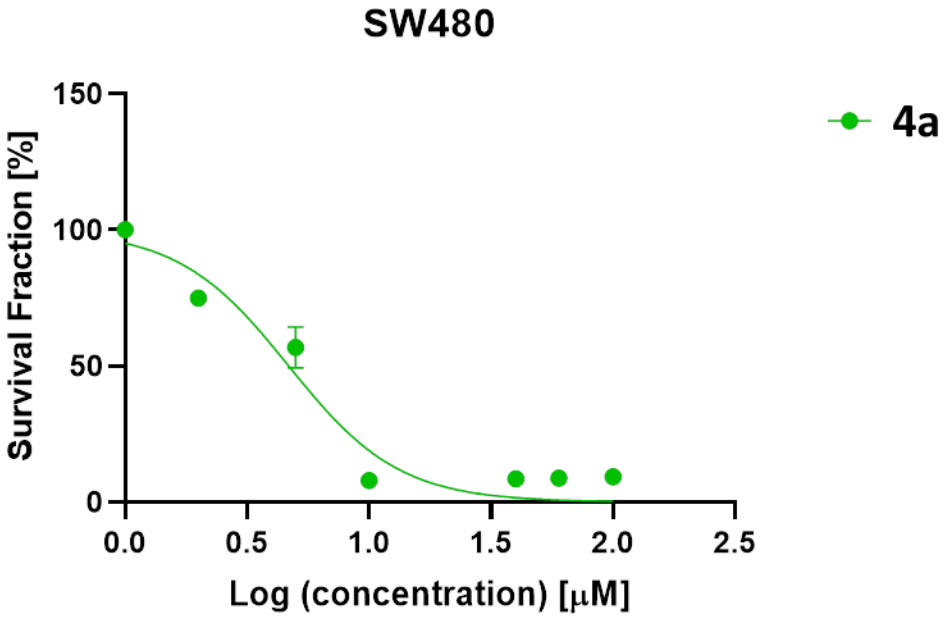

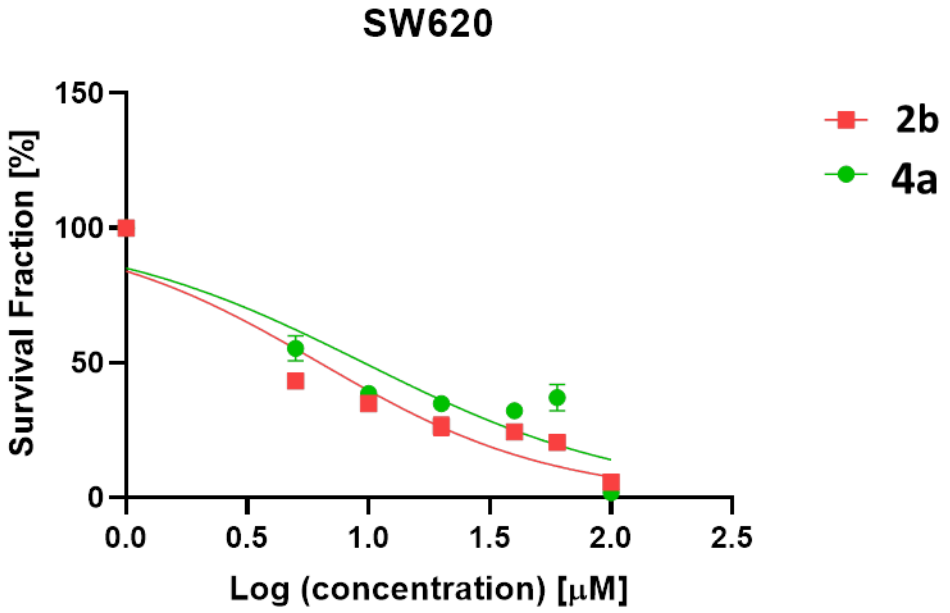

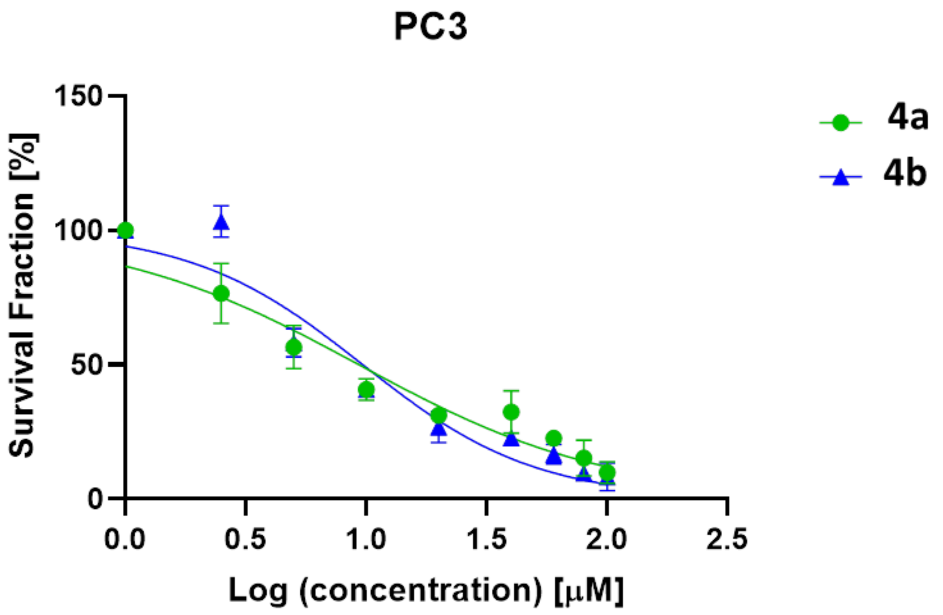
PC3
Survival Fraction [%]
150
100
50
0
0.0
0.5
1.0
1.5
2.0
2.5
Log (concentration) [μM]
4a
4b

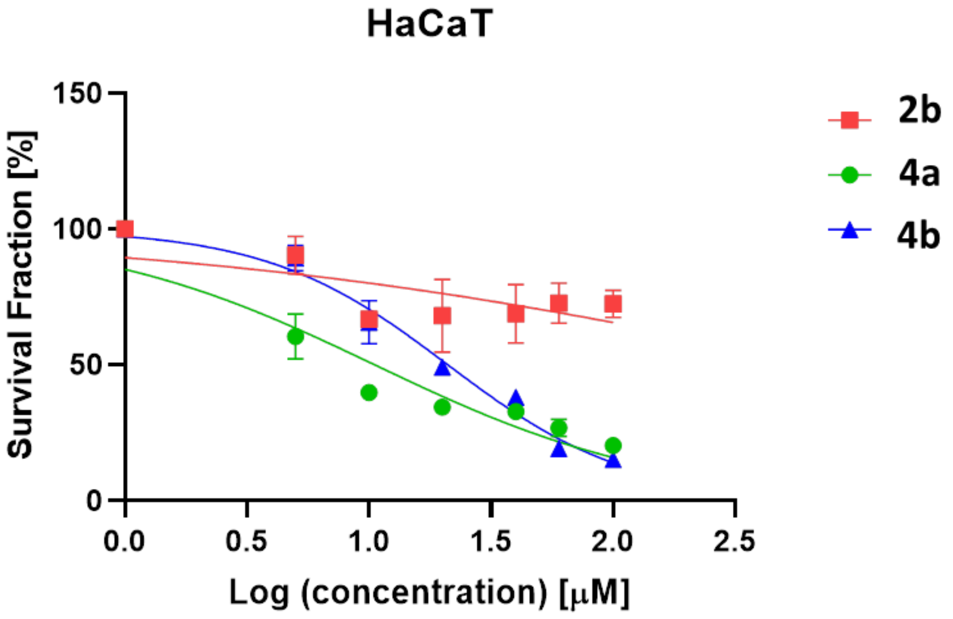
HaCaT
Survival Fraction [%]
150
100
50
0
0.0
0.5
1.0
1.5
2.0
2.5
Log (concentration) [μM]
2b
4a
4b

3. X-ray crystallography

The single crystal diffraction data for the compound **4a** was collected at room temperature with a SuperNova diffractometer (Oxford Diffraction; Agilent [1]) with the graphite monochromated CuKα radiation (λ = 1.54184 Å). The CrysAlisPro program system [2] was used for data collection, cell refinement, and data reduction. The intensities were corrected for Lorentz and polarization effects, and a multi-scan absorption corrections were applied. The crystal structure was solved by direct methods using the SHELXT program and refined by the full-matrix least squares method on F2 using the SHELXL-2018/3 program [3]. The non-hydrogen atoms were refined with anisotropic displacement parameters, H-atoms were positioned at calculated positions and refined using the riding model.

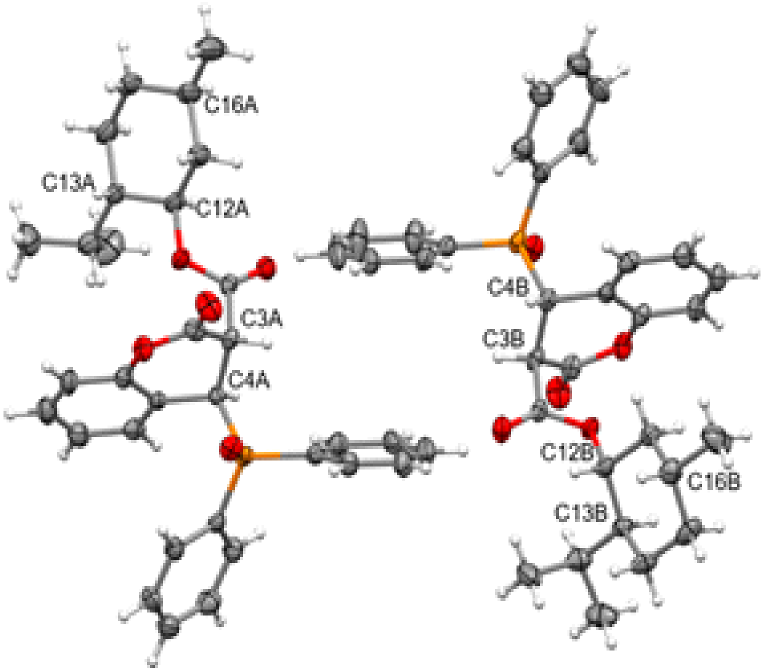

Figure S2. The molecular structure of **4a**.

Deposition Number CCDC **No. 2410610** (for **4a**),) contains the supplementary crystallographic data for this paper. These data are provided free of charge by the joint Cambridge Crystallographic Data Centre.

Table S5. Atomic coordinates (x 10^4) and equivalent isotropic displacement parameters ($Å^2$x 10^3) for **4a**. U(eq) is defined as one third of the trace of the orthogonalized U^{ij} tensor.

	x	y	z	U(eq)
C(2A)	7054(3)	4167(10)	9295(3)	46(1)
C(3A)	7126(3)	6263(9)	8995(2)	39(1)
C(4A)	6505(2)	7724(9)	8875(2)	35(1)
C(5A)	5944(3)	9552(11)	9570(3)	47(1)
C(6A)	5714(3)	9516(14)	10075(3)	62(2)
C(7A)	5824(4)	7721(15)	10437(3)	65(2)
C(8A)	6147(3)	5951(13)	10304(3)	55(2)
C(9A)	6371(3)	6023(10)	9799(2)	42(1)
C(10A)	6272(2)	7772(9)	9425(2)	34(1)
C(11A)	7759(3)	7423(9)	9357(2)	38(1)
C(12A)	8512(3)	7792(12)	10347(2)	52(1)
C(13A)	8426(3)	7878(11)	10975(2)	49(1)
C(14A)	9104(4)	8472(14)	11416(3)	68(2)
C(15A)	9639(3)	6831(18)	11392(3)	81(3)
C(16A)	9723(3)	6728(19)	10764(3)	85(3)
C(17A)	9049(3)	6238(16)	10307(3)	71(2)
C(18A)	10226(4)	5010(30)	10728(5)	140(6)
C(19A)	7843(4)	9289(14)	11015(3)	67(2)
C(20A)	7681(5)	8860(20)	11605(4)	109(4)
C(21A)	7939(6)	11689(17)	10937(5)	113(4)
C(31A)	5107(3)	7799(10)	8184(2)	40(1)
C(32A)	4929(3)	9871(11)	7971(3)	52(2)
C(33A)	4306(4)	10665(14)	7953(3)	65(2)
C(34A)	3874(3)	9412(16)	8155(3)	69(2)
C(35A)	4056(3)	7419(15)	8378(3)	66(2)
C(36A)	4659(3)	6584(13)	8396(3)	53(2)
C(41A)	6146(3)	7310(10)	7583(2)	42(1)
C(42A)	6391(3)	9356(12)	7512(3)	53(2)
C(43A)	6598(3)	9796(14)	7010(3)	62(2)
C(44A)	6550(4)	8220(17)	6573(3)	74(2)
C(45A)	6303(4)	6231(17)	6641(3)	77(2)

C(46A)	6101(3)	5752(13)	7144(3)	56(2)
O(1A)	6687(2)	4161(7)	9692(2)	48(1)
O(2A)	7299(3)	2500(8)	9215(3)	68(1)
O(3A)	5860(2)	4181(7)	8295(2)	49(1)
O(4A)	7881(2)	7020(8)	9944(2)	51(1)
O(5A)	8089(2)	8508(7)	9135(2)	49(1)
P(1A)	5888(1)	6538(2)	8228(1)	38(1)
C(2B)	7879(3)	6927(10)	5573(3)	46(1)
C(3B)	7800(3)	4841(9)	5877(2)	42(1)
C(4B)	8436(3)	3403(9)	6014(2)	38(1)
C(5B)	9046(3)	1590(11)	5358(3)	49(1)
C(6B)	9292(3)	1621(14)	4863(3)	61(2)
C(7B)	9204(4)	3407(15)	4499(3)	67(2)
C(8B)	8860(3)	5160(12)	4615(3)	53(2)
C(9B)	8611(3)	5113(9)	5105(2)	43(1)
C(10B)	8703(3)	3343(10)	5482(2)	39(1)
C(11B)	7199(3)	3579(9)	5495(2)	39(1)
C(12B)	6736(3)	2222(9)	4491(2)	42(1)
C(13B)	6168(3)	3419(10)	4047(2)	45(1)
C(14B)	5782(3)	1818(12)	3572(3)	63(2)
C(15B)	6234(4)	664(14)	3264(3)	72(2)
C(16B)	6802(3)	-493(13)	3711(3)	61(2)
C(17B)	7187(3)	1088(11)	4184(3)	50(2)
C(18B)	7256(4)	-1636(18)	3398(4)	98(3)
C(19B)	5710(3)	4676(12)	4343(3)	61(2)
C(20B)	5343(3)	3216(15)	4676(4)	78(2)
C(21B)	5213(4)	6066(13)	3879(4)	80(2)
C(31B)	9819(3)	3340(11)	6775(2)	44(1)
C(32B)	9961(3)	1256(13)	6989(3)	59(2)
C(33B)	10596(4)	426(16)	7077(3)	71(2)
C(34B)	11075(4)	1670(20)	6932(4)	81(3)
C(35B)	10930(4)	3690(20)	6702(4)	83(3)
C(36B)	10313(4)	4514(15)	6628(3)	66(2)
C(41B)	8736(3)	3870(10)	7302(2)	43(1)
C(42B)	8467(3)	1882(13)	7363(3)	58(2)
C(43B)	8256(4)	1456(16)	7860(3)	71(2)

C(44B)	8303(5)	2989(18)	8293(4)	89(3)
C(45B)	8573(6)	4963(18)	8243(4)	99(4)
C(46B)	8789(4)	5428(14)	7739(3)	72(2)
O(1B)	8271(2)	6959(7)	5193(2)	48(1)
O(2B)	7616(3)	8577(8)	5629(2)	68(1)
O(3B)	9070(2)	6955(7)	6598(2)	54(1)
O(4B)	7165(2)	3729(7)	4914(2)	46(1)
O(5B)	6845(2)	2579(8)	5714(2)	54(1)
P(1B)	9027(1)	4608(2)	6676(1)	41(1)

Table S6. Bond lengths [Ĺ] and angles [°] for **4a**.

C(2A)-O(2A)	1.195(8)
C(2A)-O(1A)	1.367(7)
C(2A)-C(3A)	1.503(8)
C(3A)-C(11A)	1.531(7)
C(3A)-C(4A)	1.549(7)
C(3A)-H(3A)	0.9800
C(4A)-C(10A)	1.497(6)
C(4A)-P(1A)	1.829(5)
C(4A)-H(4A)	0.9800
C(5A)-C(6A)	1.395(9)
C(5A)-C(10A)	1.396(8)
C(5A)-H(5A)	0.9300
C(6A)-C(7A)	1.374(12)
C(6A)-H(6A)	0.9300
C(7A)-C(8A)	1.375(11)
C(7A)-H(7A)	0.9300
C(8A)-C(9A)	1.388(8)
C(8A)-H(8A)	0.9300
C(9A)-C(10A)	1.368(8)
C(9A)-O(1A)	1.392(7)
C(11A)-O(5A)	1.190(7)
C(11A)-O(4A)	1.335(6)
C(12A)-O(4A)	1.464(6)
C(12A)-C(17A)	1.510(10)

C(12A)-C(13A)	1.522(8)
C(12A)-H(12A)	0.9800
C(13A)-C(19A)	1.533(9)
C(13A)-C(14A)	1.540(8)
C(13A)-H(13A)	0.9800
C(14A)-C(15A)	1.531(11)
C(14A)-H(14A)	0.9700
C(14A)-H(14B)	0.9700
C(15A)-C(16A)	1.522(10)
C(15A)-H(15A)	0.9700
C(15A)-H(15B)	0.9700
C(16A)-C(18A)	1.521(14)
C(16A)-C(17A)	1.535(9)
C(16A)-H(16A)	0.9800
C(17A)-H(17A)	0.9700
C(17A)-H(17B)	0.9700
C(18A)-H(18A)	0.9600
C(18A)-H(18B)	0.9600
C(18A)-H(18C)	0.9600
C(19A)-C(21A)	1.520(13)
C(19A)-C(20A)	1.531(11)
C(19A)-H(19A)	0.9800
C(20A)-H(20A)	0.9600
C(20A)-H(20B)	0.9600
C(20A)-H(20C)	0.9600
C(21A)-H(21A)	0.9600
C(21A)-H(21B)	0.9600
C(21A)-H(21C)	0.9600
C(31A)-C(32A)	1.389(9)
C(31A)-C(36A)	1.405(8)
C(31A)-P(1A)	1.797(6)
C(32A)-C(33A)	1.391(10)
C(32A)-H(32A)	0.9300
C(33A)-C(34A)	1.380(12)
C(33A)-H(33A)	0.9300
C(34A)-C(35A)	1.351(12)

C(34A)-H(34A)	0.9300
C(35A)-C(36A)	1.361(10)
C(35A)-H(35A)	0.9300
C(36A)-H(36A)	0.9300
C(41A)-C(46A)	1.387(9)
C(41A)-C(42A)	1.398(9)
C(41A)-P(1A)	1.804(6)
C(42A)-C(43A)	1.388(8)
C(42A)-H(42A)	0.9300
C(43A)-C(44A)	1.392(12)
C(43A)-H(43A)	0.9300
C(44A)-C(45A)	1.366(13)
C(44A)-H(44A)	0.9300
C(45A)-C(46A)	1.389(10)
C(45A)-H(45A)	0.9300
C(46A)-H(46A)	0.9300
O(3A)-P(1A)	1.473(4)
C(2B)-O(2B)	1.189(8)
C(2B)-O(1B)	1.376(7)
C(2B)-C(3B)	1.506(8)
C(3B)-C(11B)	1.531(8)
C(3B)-C(4B)	1.563(8)
C(3B)-H(3B)	0.9800
C(4B)-C(10B)	1.502(7)
C(4B)-P(1B)	1.831(5)
C(4B)-H(4B)	0.9800
C(5B)-C(10B)	1.381(8)
C(5B)-C(6B)	1.393(9)
C(5B)-H(5B)	0.9300
C(6B)-C(7B)	1.373(11)
C(6B)-H(6B)	0.9300
C(7B)-C(8B)	1.378(10)
C(7B)-H(7B)	0.9300
C(8B)-C(9B)	1.386(8)
C(8B)-H(8B)	0.9300
C(9B)-C(10B)	1.383(8)

C(9B)-O(1B)	1.396(7)
C(11B)-O(5B)	1.193(7)
C(11B)-O(4B)	1.336(6)
C(12B)-O(4B)	1.461(6)
C(12B)-C(17B)	1.517(8)
C(12B)-C(13B)	1.524(7)
C(12B)-H(12B)	0.9800
C(13B)-C(14B)	1.530(8)
C(13B)-C(19B)	1.549(9)
C(13B)-H(13B)	0.9800
C(14B)-C(15B)	1.525(11)
C(14B)-H(14C)	0.9700
C(14B)-H(14D)	0.9700
C(15B)-C(16B)	1.517(10)
C(15B)-H(15C)	0.9700
C(15B)-H(15D)	0.9700
C(16B)-C(17B)	1.518(9)
C(16B)-C(18B)	1.535(10)
C(16B)-H(16B)	0.9800
C(17B)-H(17C)	0.9700
C(17B)-H(17D)	0.9700
C(18B)-H(18D)	0.9600
C(18B)-H(18E)	0.9600
C(18B)-H(18F)	0.9600
C(19B)-C(21B)	1.530(10)
C(19B)-C(20B)	1.540(11)
C(19B)-H(19B)	0.9800
C(20B)-H(20D)	0.9600
C(20B)-H(20E)	0.9600
C(20B)-H(20F)	0.9600
C(21B)-H(21D)	0.9600
C(21B)-H(21E)	0.9600
C(21B)-H(21F)	0.9600
C(31B)-C(32B)	1.386(10)
C(31B)-C(36B)	1.393(9)
C(31B)-P(1B)	1.798(6)

C(32B)-C(33B)	1.393(10)
C(32B)-H(32B)	0.9300
C(33B)-C(34B)	1.387(13)
C(33B)-H(33B)	0.9300
C(34B)-C(35B)	1.364(14)
C(34B)-H(34B)	0.9300
C(35B)-C(36B)	1.359(12)
C(35B)-H(35B)	0.9300
C(36B)-H(36B)	0.9300
C(41B)-C(42B)	1.381(9)
C(41B)-C(46B)	1.382(9)
C(41B)-P(1B)	1.797(6)
C(42B)-C(43B)	1.380(9)
C(42B)-H(42B)	0.9300
C(43B)-C(44B)	1.366(13)
C(43B)-H(43B)	0.9300
C(44B)-C(45B)	1.369(15)
C(44B)-H(44B)	0.9300
C(45B)-C(46B)	1.405(11)
C(45B)-H(45B)	0.9300
C(46B)-H(46B)	0.9300
O(3B)-P(1B)	1.473(5)

4. In silico prediction and ADME properties

Table S7. Predicted physicochemical properties of compounds **1a-f**, **2a, 2b 3**, **4a-d**, **5a-b**

comp.	mean anticancer IC_{50}	M.W.	Heavy atoms	Aromatic heavy atoms	Csp3 fraction	Rotatable bonds	H-bond acceptors	Molar Refractivity	TPSA	Average (Consensus) LogP	iLOGP	XLOGP3	WLOGP	MLOGP	Silicos-IT Log P	ESOL Log S	Ali Log S	Silicos-IT LogSw
**1a**	65.525	328.4	24	10	0.5	4	4	94.91	56.51	4.29	3.5	5.45	4.41	3.78	4.29	-5.35	-6.39	-5.46
**1b**	100	328.4	24	10	0.5	4	4	94.91	56.51	4.24	3.27	5.45	4.41	3.78	4.29	-5.35	-6.39	-5.46
**1c**	100	358.43	26	10	0.52	5	5	101.4	65.74	4.29	3.82	5.42	4.42	3.44	4.36	-5.43	-6.56	-5.57
**1d**	47.825	358.43	26	10	0.52	5	5	101.4	65.74	4.25	3.64	5.42	4.42	3.44	4.36	-5.43	-6.56	-5.57
**1e**	43.725	344.4	25	10	0.5	4	5	96.94	76.74	3.82	2.84	5.09	4.12	3.22	3.81	-5.21	-6.44	-4.88
**1f**	100	272.3	20	10	0.38	3	4	75.68	56.51	3.22	2.71	3.78	3.28	2.84	3.52	-4.08	-4.66	-4.74
**2a**	100	282.22	21	10	0.35	6	5	79.63	82.74	2.25	2.70	1.93	2.69	1.09	2.27	-2.93	-3.45	-4.77
**2b**	77.55	312.25	21	10	0.36	6	6	79.73	84.78	2.14	2.73	1.93	2.69	1.09	2.27	-2.95	-3.33	-4.8
**3**	100	376.34	27	22	0.05	4	4	108.37	66.32	3.59	2.69	4.22	3.44	2.97	4.61	-5.17	-5.32	-8.73
**4a**	11.625	530.59	38	18	0.38	7	5	151.54	79.48	5.54	4.2	7.08	5.96	4.79	5.66	-7.48	-8.57	-9.11
**4b**	28.675	560.62	40	18	0.39	8	6	158.03	88.71	5.51	4.34	7.06	5.97	4.43	5.74	-7.57	-8.74	-9.2
**4c**	100	420.39	30	18	0.17	6	5	115.2	79.48	3.54	2.97	3.97	3.52	3.22	4.05	-5	-5.34	-7.83
**4d**	100	356.31	24	6	0.5	8	7	86.56	97.94	2.1	2.83	1.68	2.77	1.46	1.74	-2.76	-3.35	-3.92
**5a**	100	484.42	33	18	0.24	8	6	129.56	98.52	3.71	3.32	3.6	4.97	3.02	3.62	-4.99	-5.36	-8.37
**5b**	100	514.44	35	18	0.27	9	7	136.05	107.75	3.74	3.74	3.57	4.98	2.69	3.7	-5.07	-5.52	-8.46

Table S8. Selected in silico ADME properties prediction and drug-likeness filters of compounds **1a-f**, **2a, 2b**, **3**, **4 a-d**, **5a-b**

Compound	Pharmacokinetics predictions			CYP450 inhibition					Drug likeness filters violations				
	GIT Absorption	BBB permeation	P-glycoprotein substrate	CYP3A4	CYP2D6	Cyp1A2	Cyp2C19	Cyp 2C9	Lipiniski	Ghose	Veber	Egan	Muegge
**1a**	High	Yes	No	No	No	No	Yes	Yes	0	0	0	0	1
**1b**	High	Yes	No	No	No	No	Yes	Yes	0	0	0	0	1
**1c**	High	Yes	No	Yes	No	No	Yes	Yes	0	0	0	0	1
**1d**	High	Yes	No	Yes	No	No	Yes	Yes	0	0	0	0	1
**1e**	High	No	No	No	No	No	Yes	Yes	0	0	0	0	1
**1f**	High	Yes	No	No	No	Yes	Yes	Yes	0	0	0	0	0
**2a**	High	No	No	No	No	Yes	Yes	Yes	0	0	0	0	0
**2b**	High	No	No	No	No	Yes	Yes	Yes	0	0	0	0	0
**3**	High	Yes	No	Yes	No	Yes	Yes	Yes	0	0	0	0	0
**4a**	High	No	Yes	Yes	No	No	No	Yes	2	4	0	1	1
**4b**	High	No	Yes	Yes	Yes	No	Yes	Yes	2	4	0	1	1
**4c**	High	No	No	Yes	No	No	Yes	Yes	0	0	0	0	0
**4d**	High	No	No	No	No	No	Yes	No	0	0	0	0	0
**5a**	High	No	No	Yes	Yes	No	Yes	Yes	0	1	0	0	0
**5b**	High	No	Yes	Yes	Yes	No	Yes	Yes	1	2	0	0	0

Table S9. Radar map of bioavailability-related properties and BOILED-Egg diagram.

Left column, radar map of bioavailability-related properties. The pink area shows the optimal range for each descriptor (LIPO = -0.7 XLOGP3 5.0; SIZE = 150 g/mol < MW < 500 g/mol, POLAR = 20 Å^2 < TPSA <130 Å^2; INSOLU =: -6 < log S (ESOL) < 0; INSATU =: fraction of sp3 carbons > 0.25; and FLEX = no. of rotatable bonds <9. Right column, BOILED-Egg diagram showing predictions for passive human gastrointestinal absorption – white area; and blood-brain barrier permeation – yellow area, based on compound lipophilicity (WLOGP) and topological polar surface area (TPSA)

4a

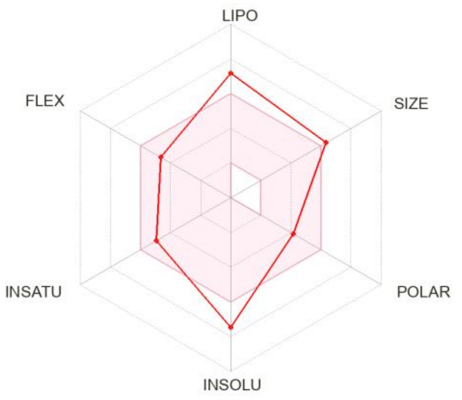

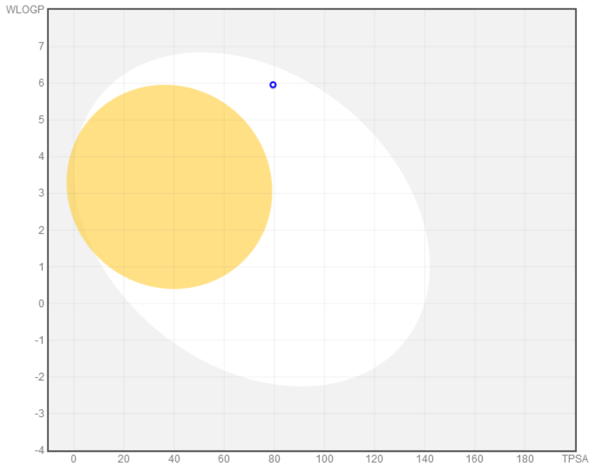

4b

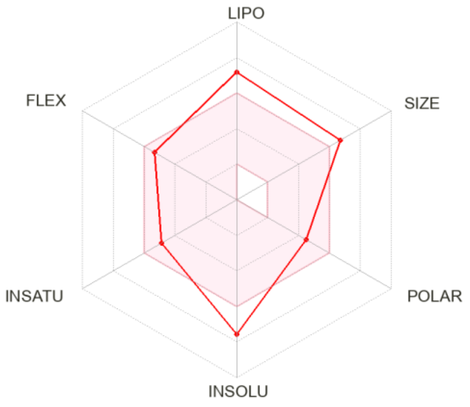

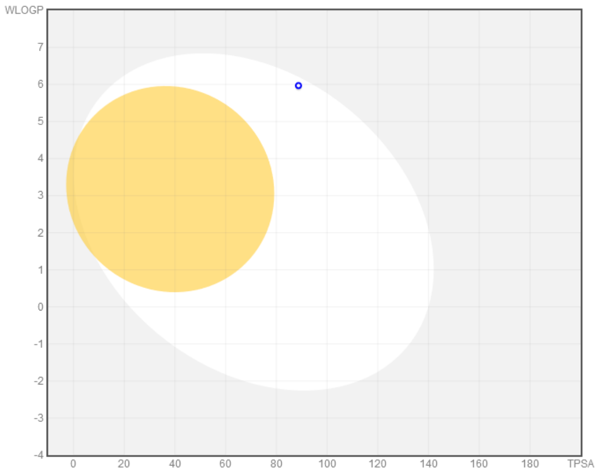

1e

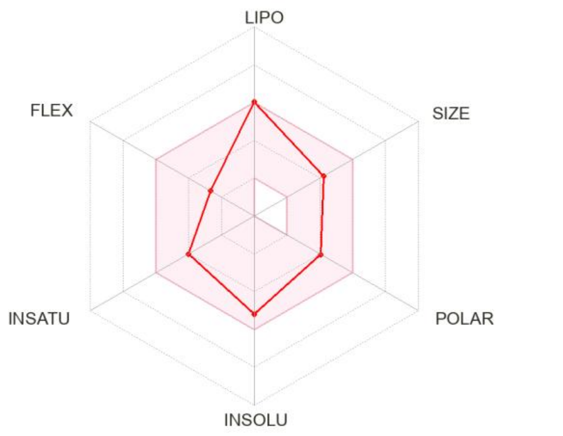
LIPO
FLEX
SIZE
INSATU
POLAR
INSOLU

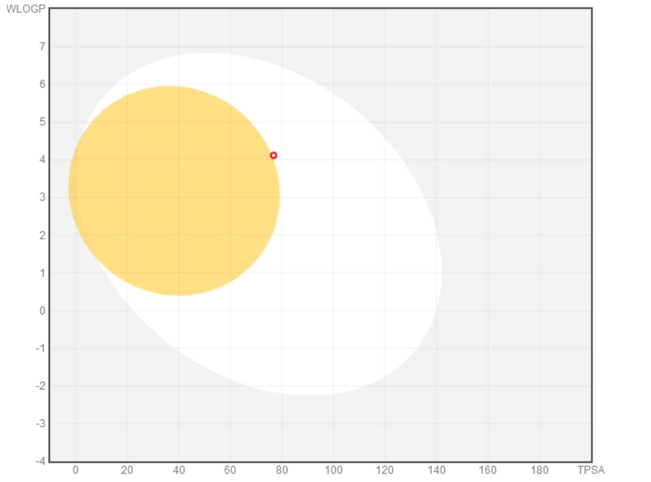
WLOGP
7
6
5
4
3
2
1
0
-1
-2
-3
-4
0
20
40
60
80
100
120
140
160
180
TPSA

1d

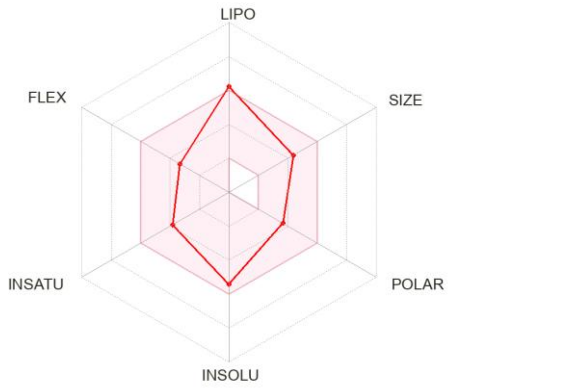
LIPO
FLEX
SIZE
INSATU
POLAR
INSOLU

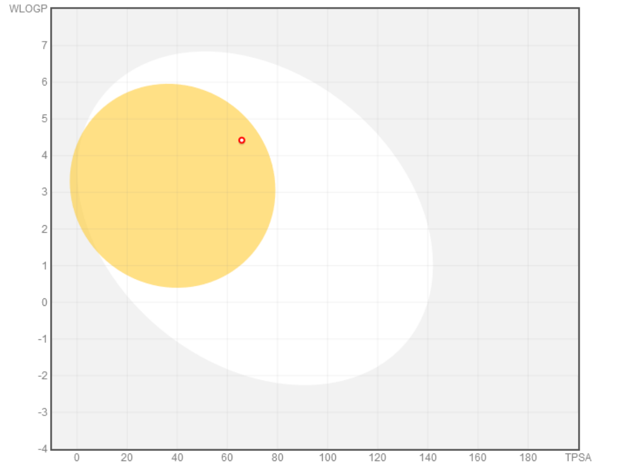
WLOGP
7
6
5
4
3
2
1
0
-1
-2
-3
-4
0
20
40
60
80
100
120
140
160
180
TPSA

1a

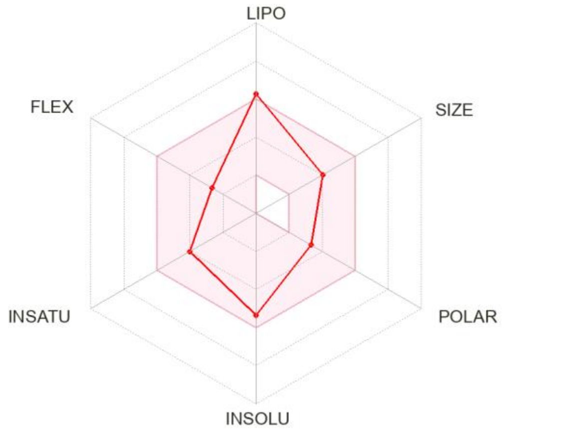
LIPO
FLEX
SIZE
INSATU
POLAR
INSOLU

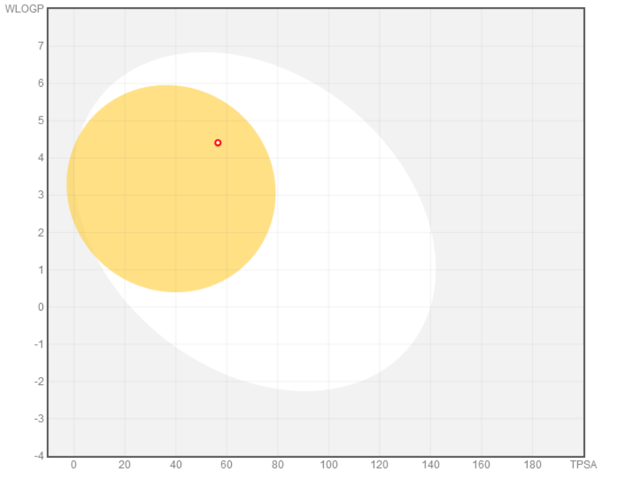
WLOGP
7
6
5
4
3
2
1
0
-1
-2
-3
-4
0
20
40
60
80
100
120
140
160
180
TPSA

2b

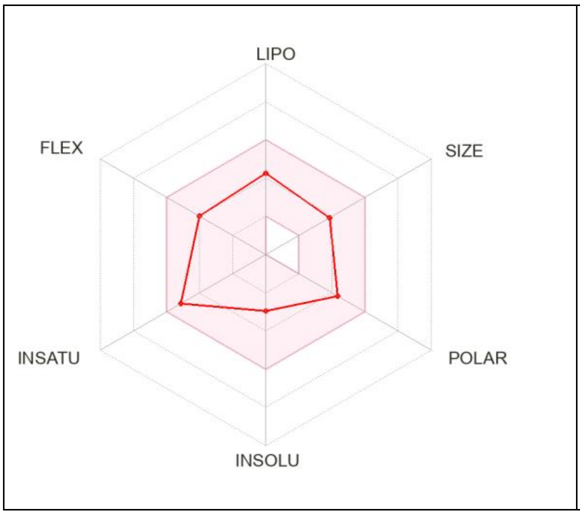

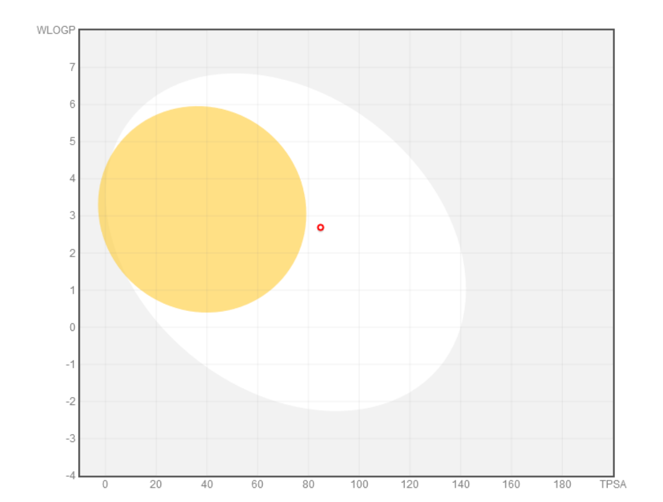

5. NMR spectra of the compounds 1-5.

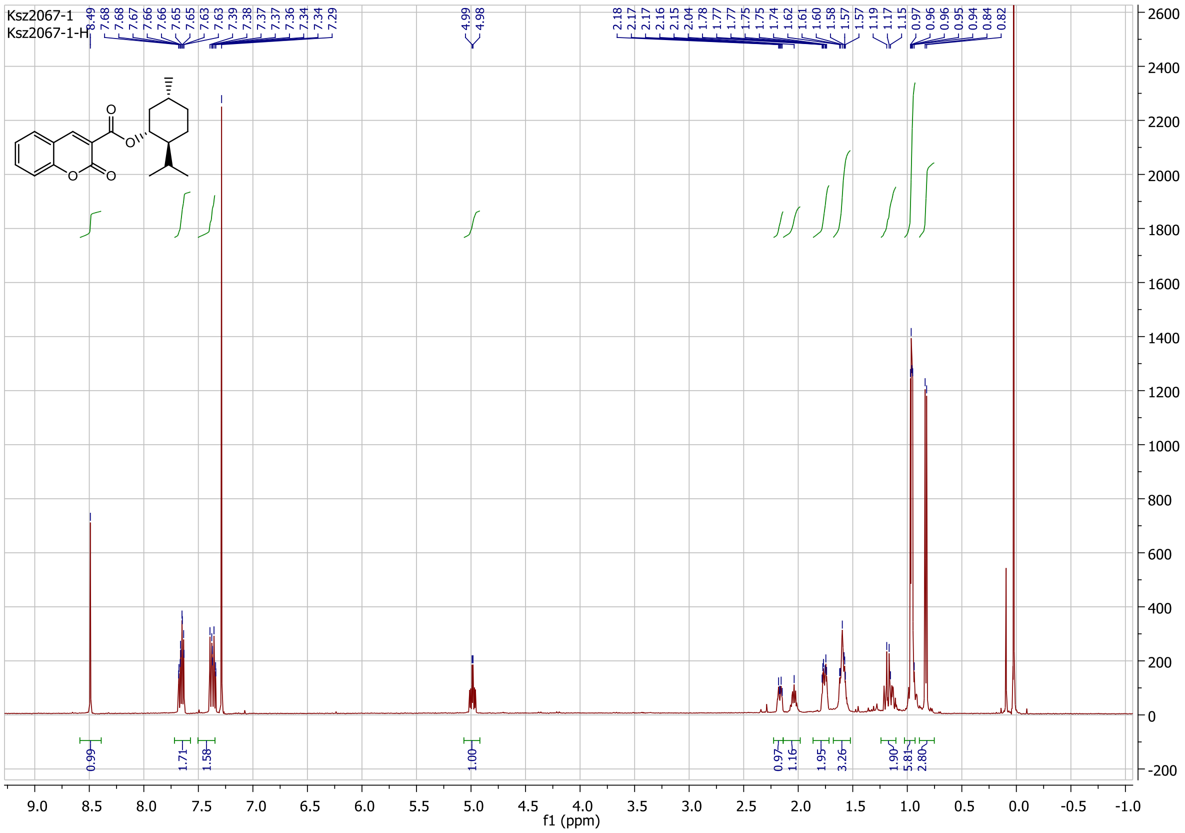

^{1}H NMR of (1*R*,2*S*,5*R*)-2-isopropyl-5-methylcyclohexyl 2-oxo-2*H*-chromene-3-carboxylate (**1a**).

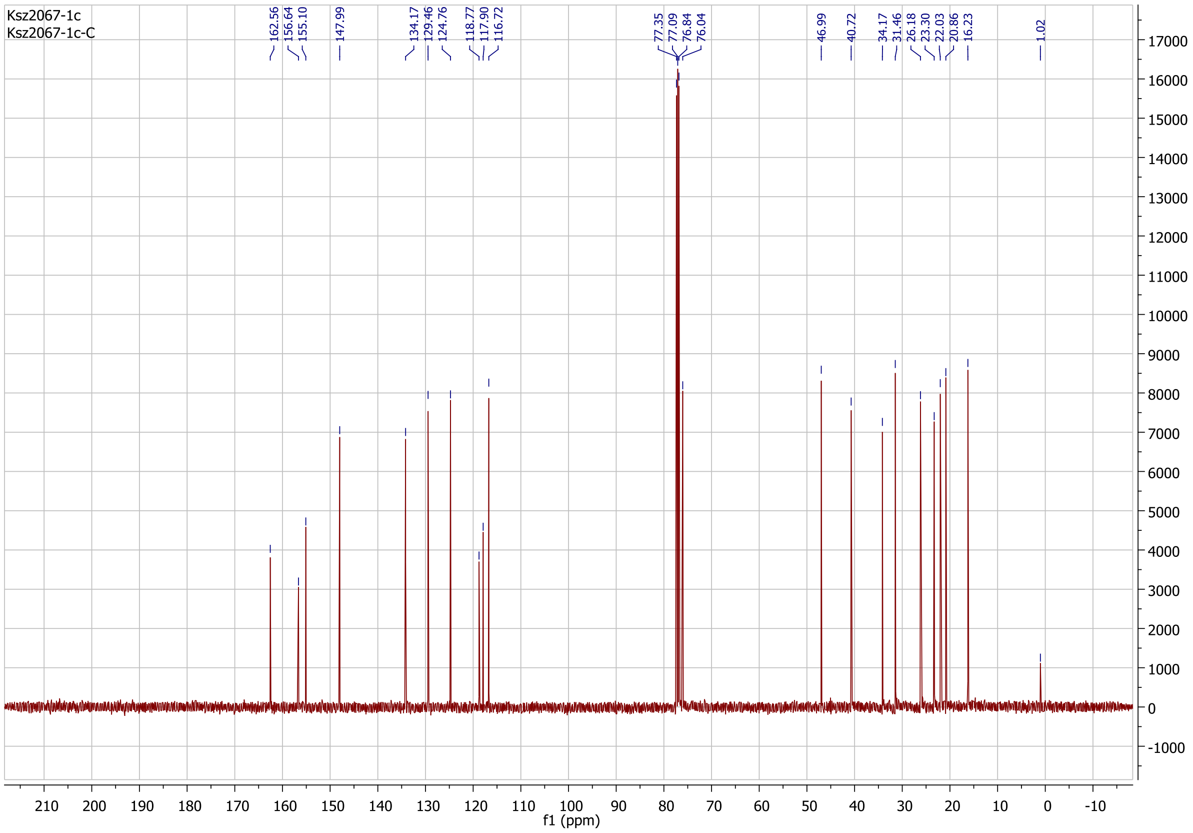

[13] C NMR of (1*R*,2*S*,5*R*)-2-isopropyl-5-methylcyclohexyl 2-oxo-2*H*-chromene-3-carboxylate (**1a**).

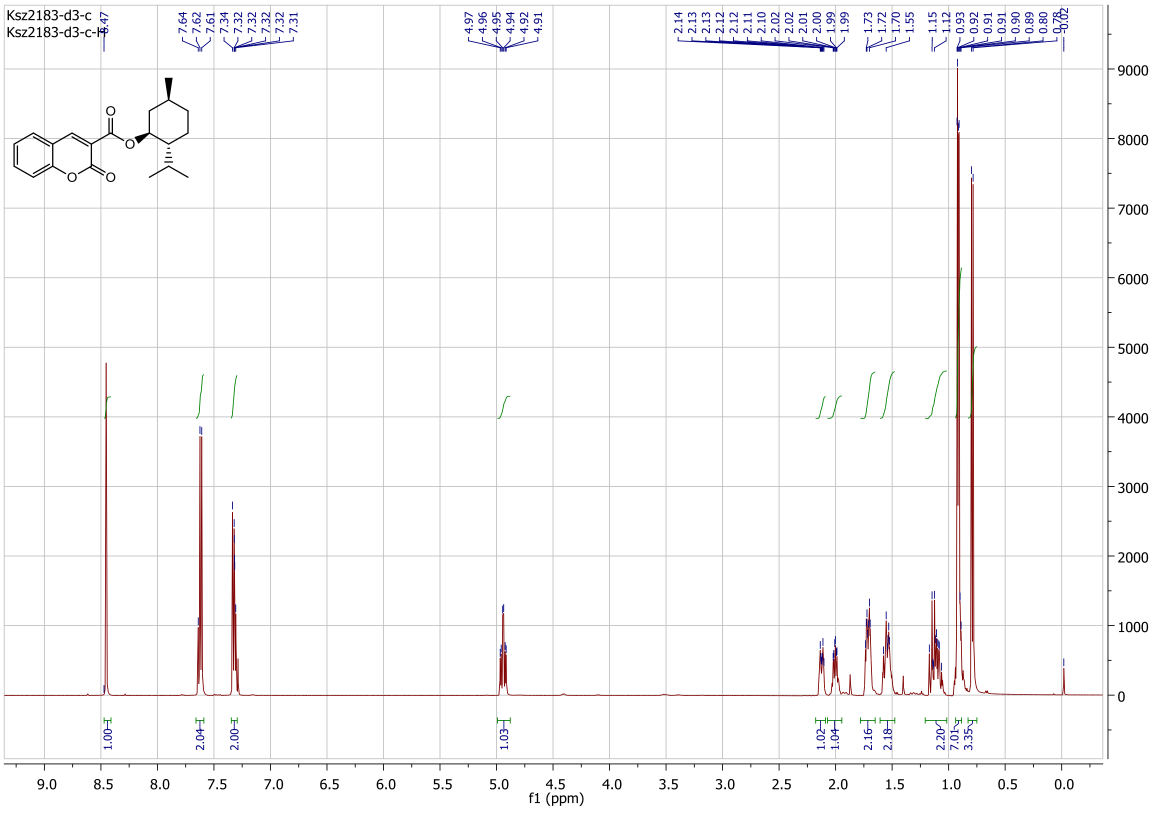

^{1}H NMR of (1*S*,2*R*,5*S*)-2-isopropyl-5-methylcyclohexyl 2-oxo-2H-chromene-3-carboxylate (**1b**)

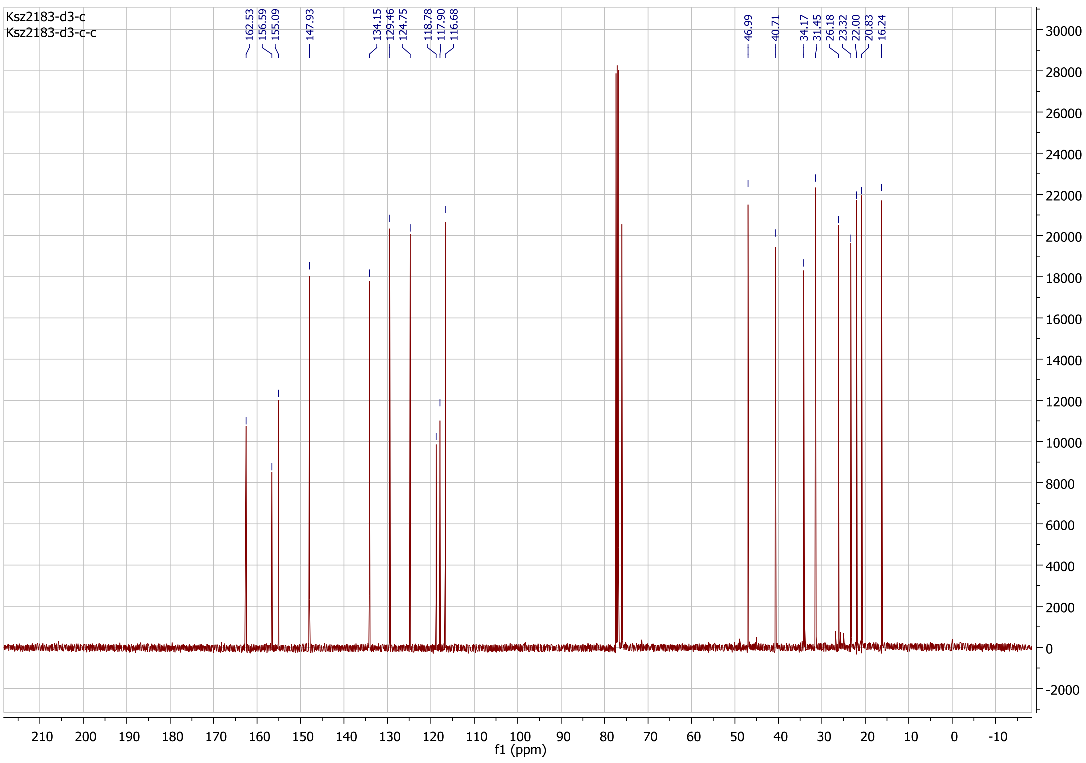

^{13}C NMR of (1*S*,2*R*,5*S*)-2-isopropyl-5-methylcyclohexyl 2-oxo-2H-chromene-3-carboxylate (**1b**)

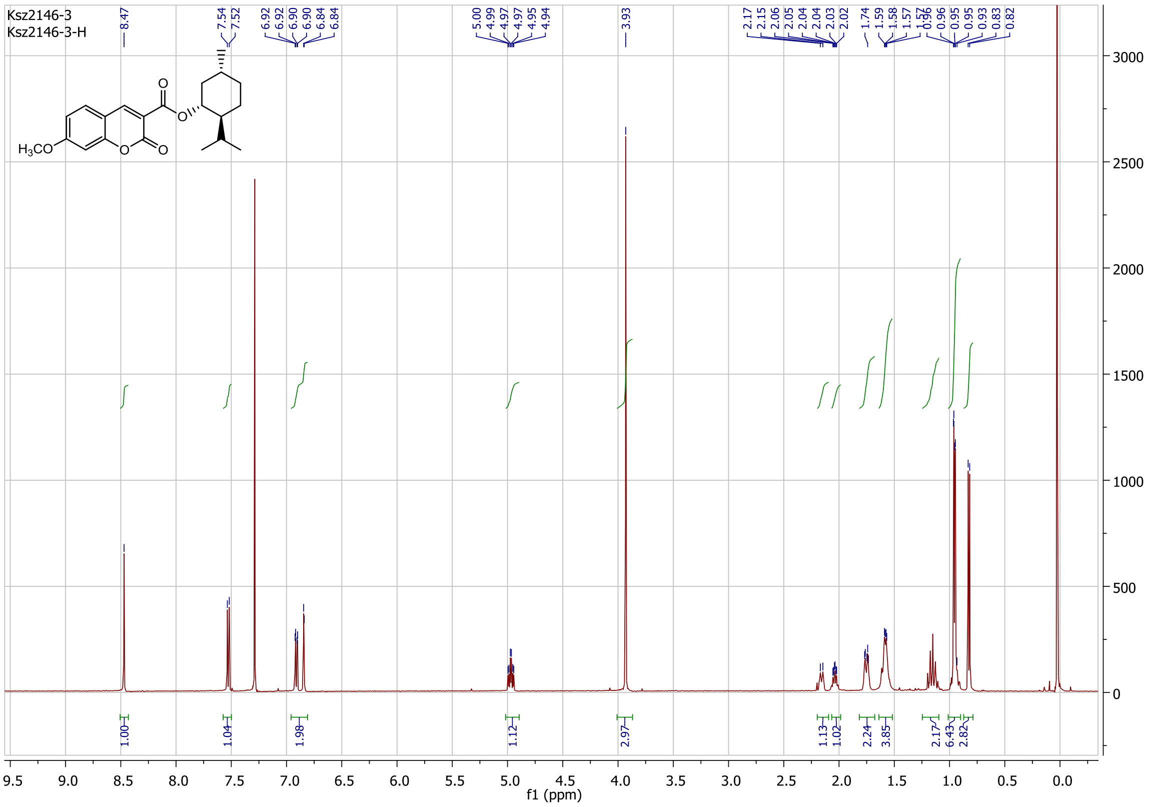

^{1}H NMR of (1*R*,2*S*,5*R*)-2-isopropyl-5-methylcyclohexyl 7-methoxy-2-oxo-2H-chromene-3-carboxylate (**1c**)

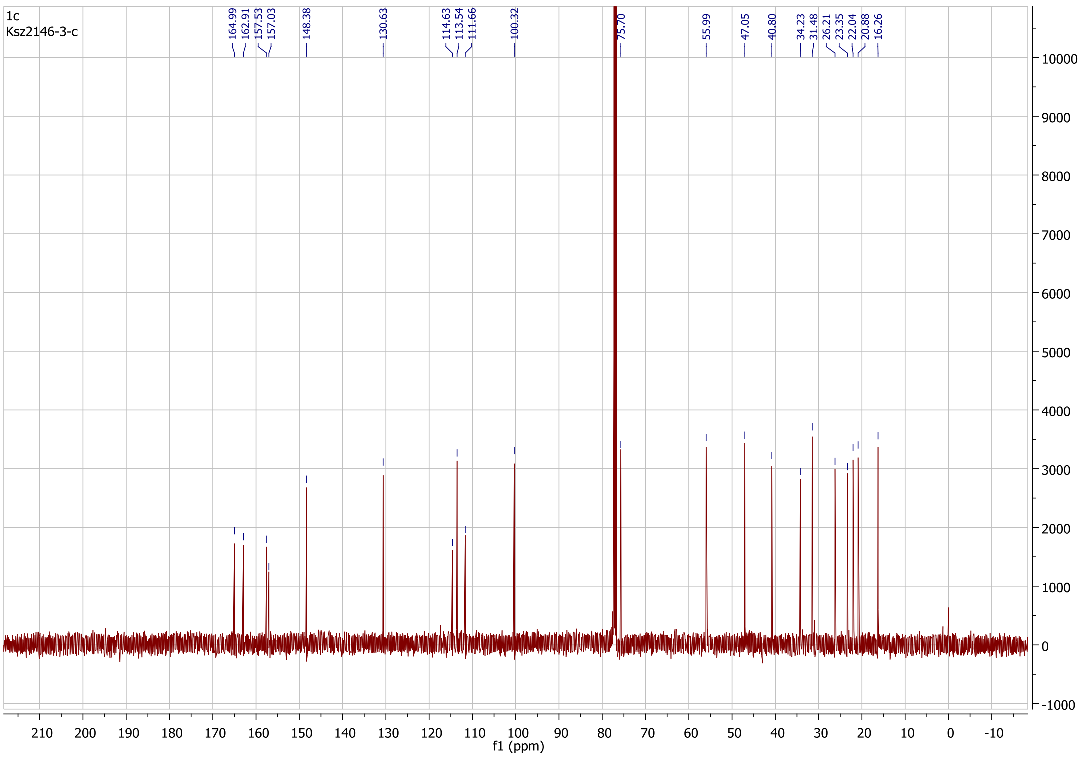

^{13}C NMR of (1*R*,2*S*,5*R*)-2-isopropyl-5-methylcyclohexyl 7-methoxy-2-oxo-2H-chromene-3-carboxylate (**1c**)

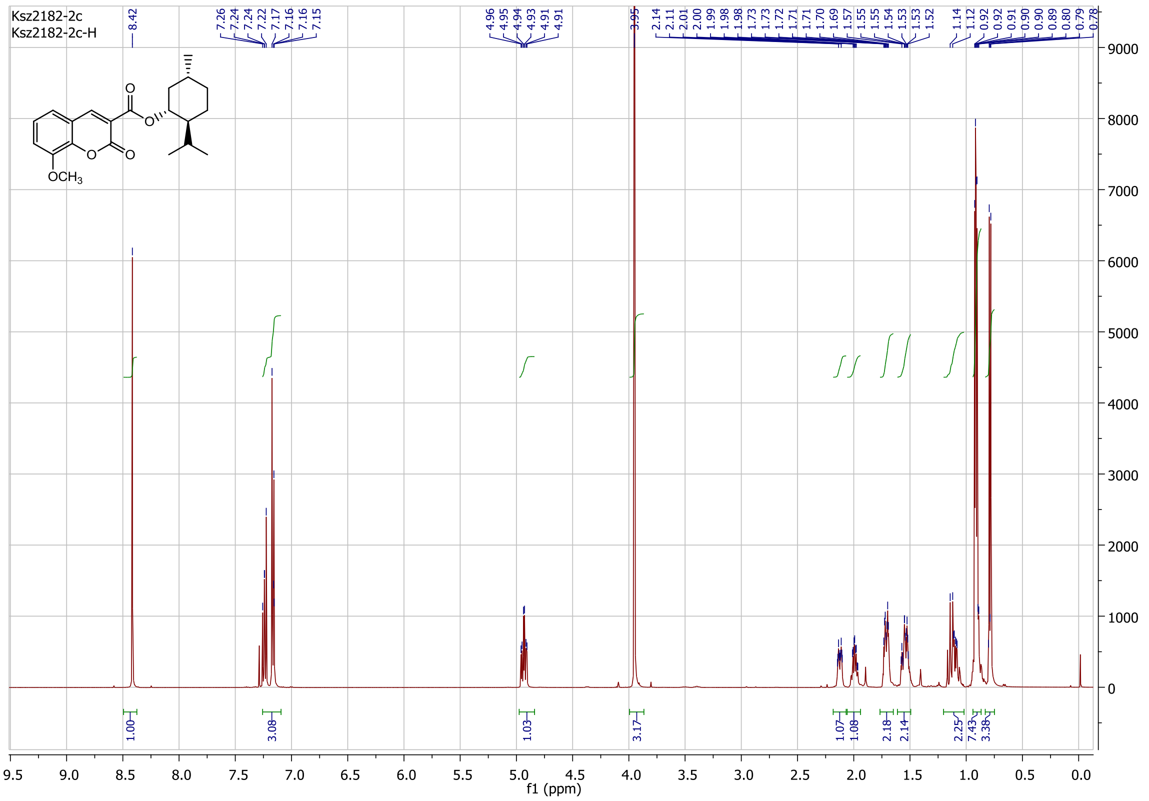

^{1}H NMR of (1*R*,2*S*,5*R*)-2-isopropyl-5-methylcyclohexyl 8-methoxy-2-oxo-2H-chromene-3-carboxylate (**1d**).

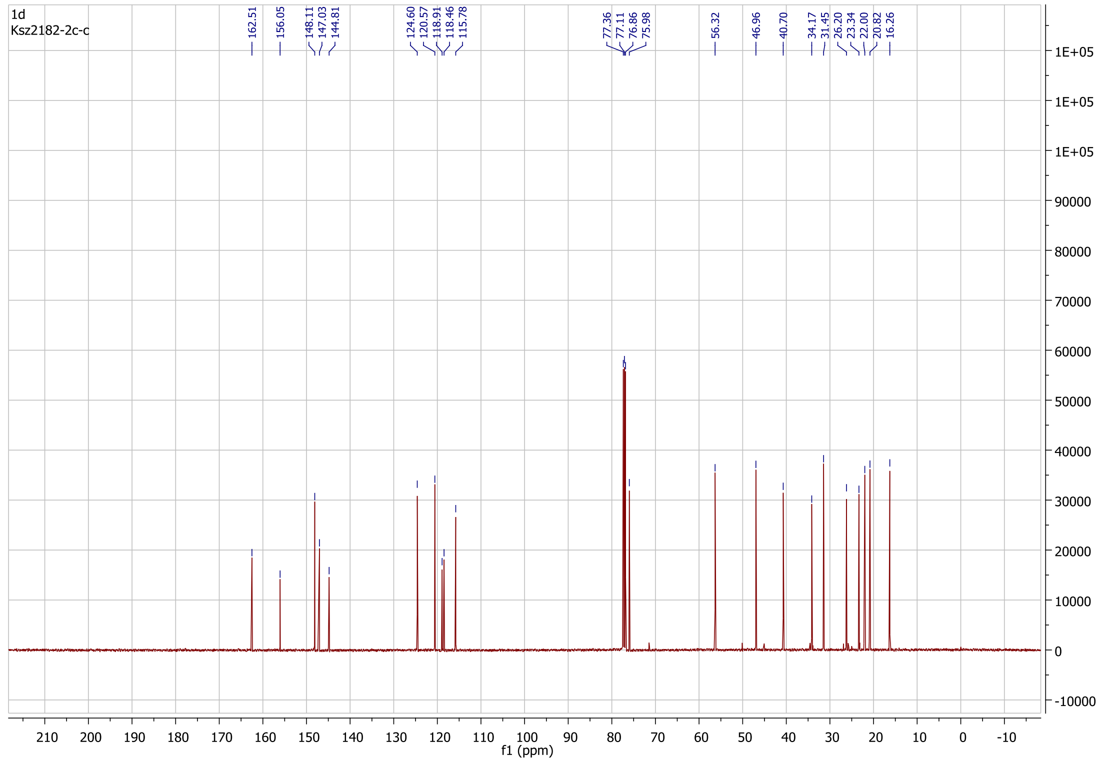

^{13}C NMR of (1*R*,2*S*,5*R*)-2-isopropyl-5-methylcyclohexyl 8-methoxy-2-oxo-2H-chromene-3-carboxylate (**1d**).

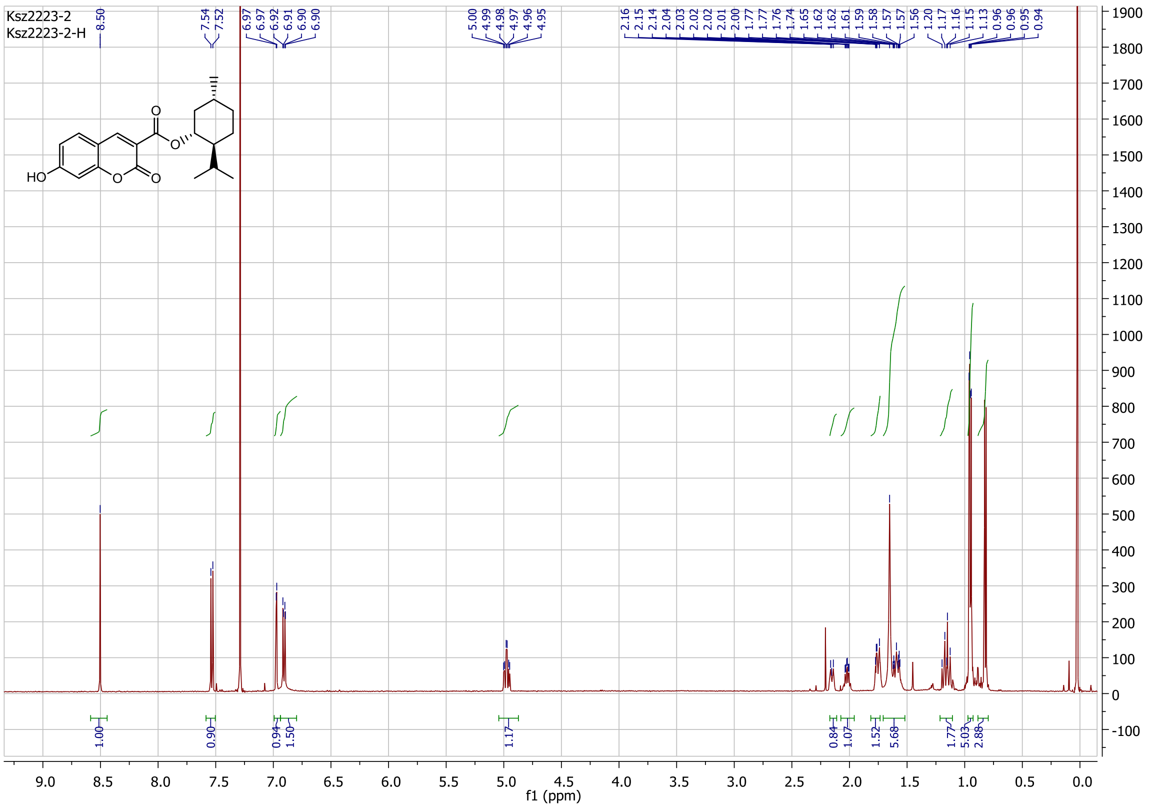

^{1}H NMR of (1*R*,2*S*,5*R*)-2-isopropyl-5-methylcyclohexyl 7-hydroxy-2-oxo-2H-chromene-3-carboxylate (**1e**).

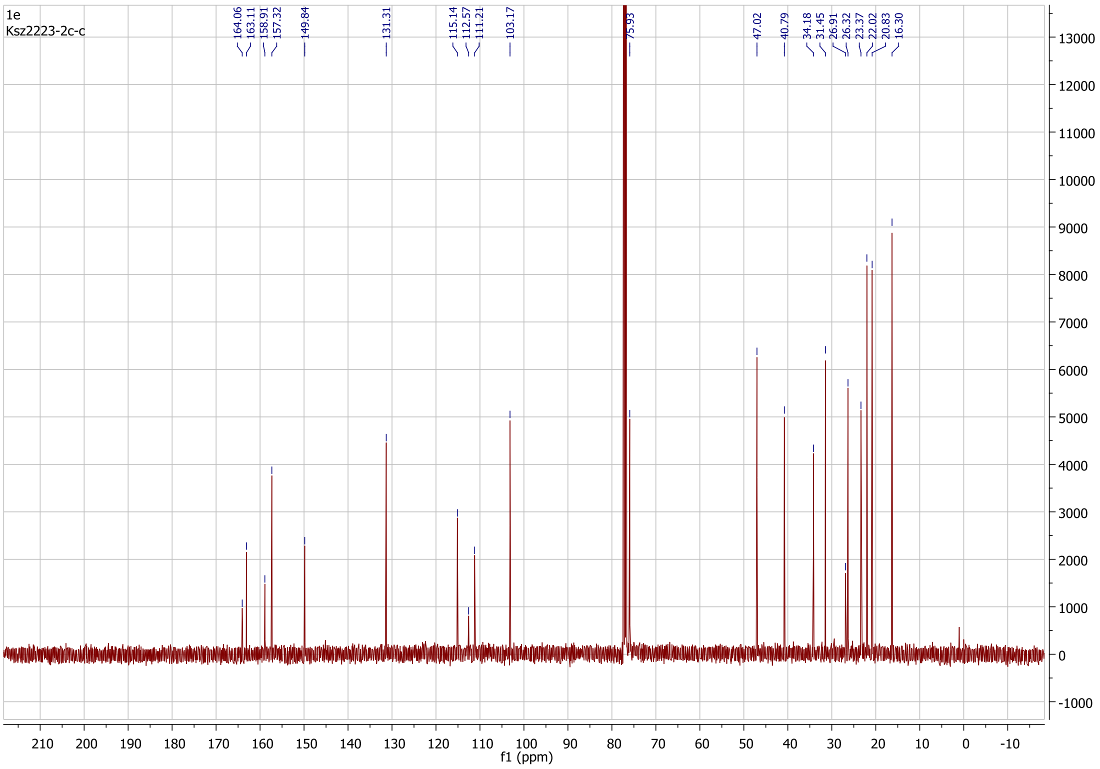

^{13}C NMR of (1*R*,2*S*,5*R*)-2-isopropyl-5-methylcyclohexyl 7-hydroxy-2-oxo-2H-chromene-3-carboxylate (**1e**).

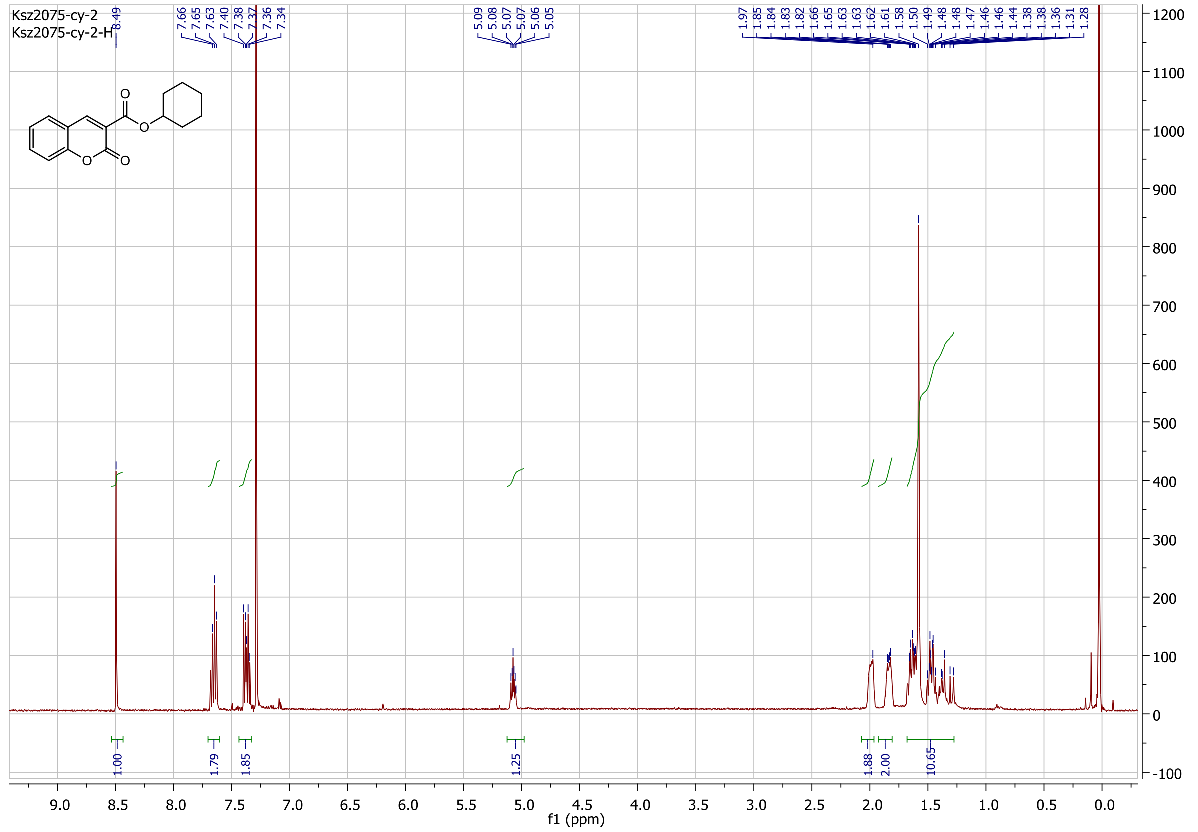

^{1}H NMR of cyclohexyl 2-oxo-2H-chromene-3-carboxylate (**1f**).

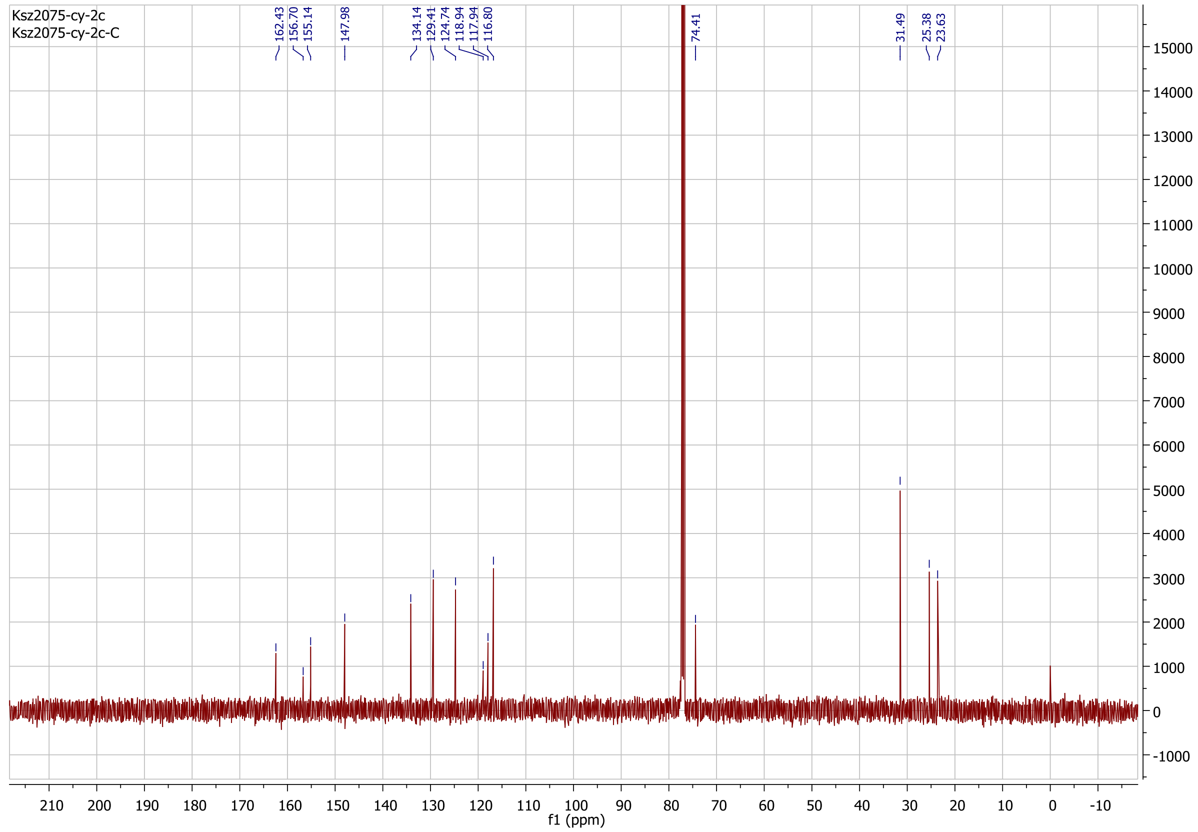

^{13}C NMR of cyclohexyl 2-oxo-2H-chromene-3-carboxylate (**1f**).

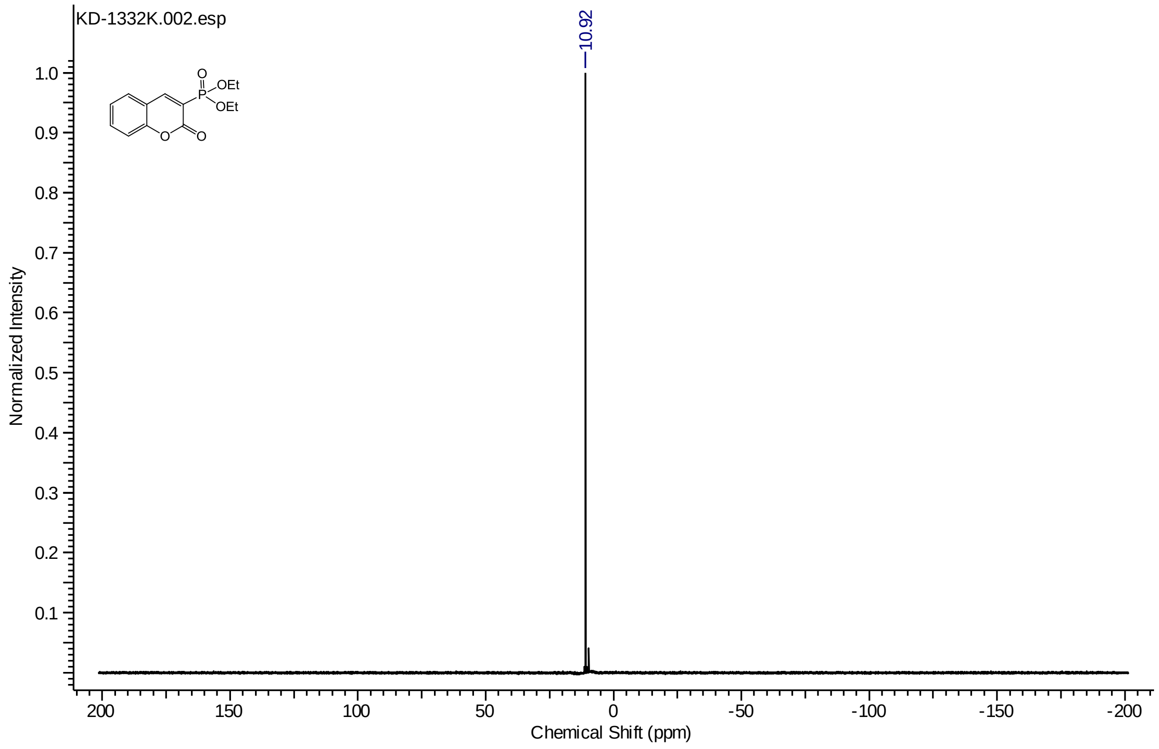

^{31}P NMR of diethyl (2-oxo-2*H*-chromen-3-yl)phosphonate **(2a).**

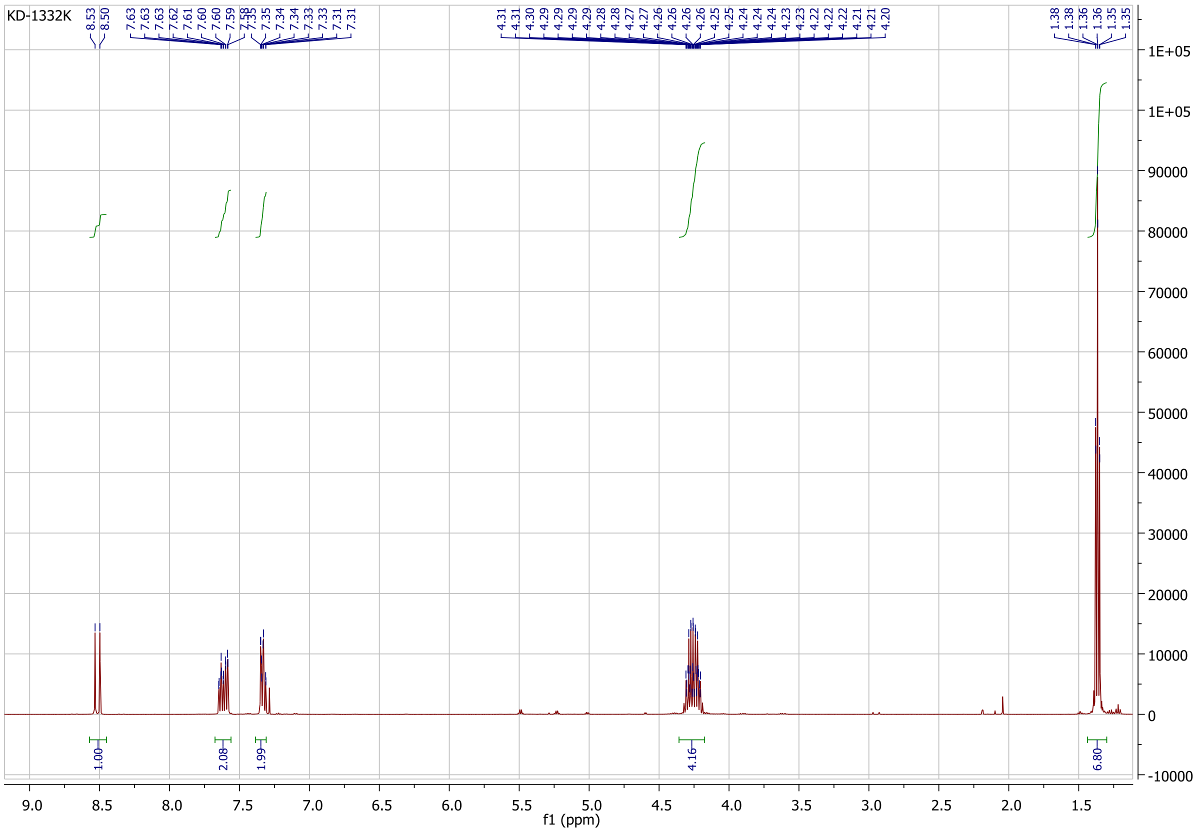

^{1}H NMR of diethyl (2-oxo-2*H*-chromen-3-yl)phosphonate **(2a).**

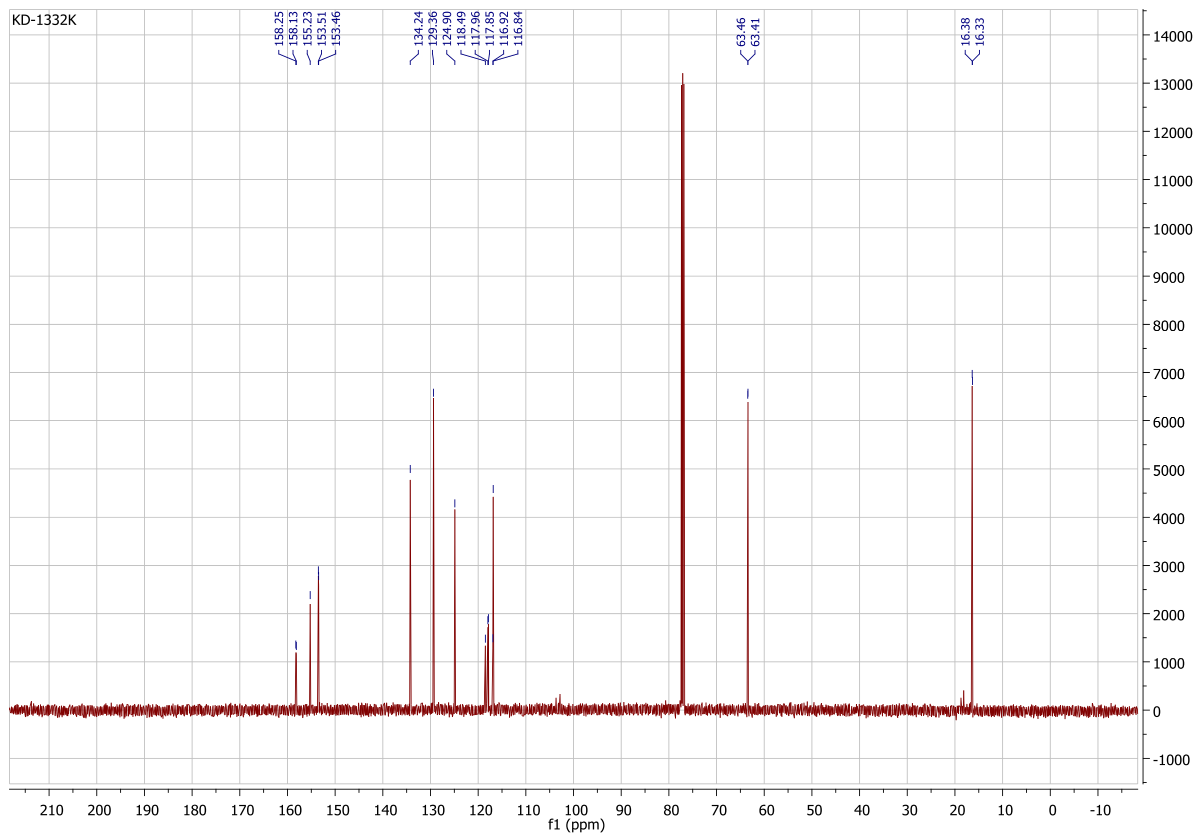

^{13}C NMR of diethyl (2-oxo-2*H*-chromen-3-yl)phosphonate **(2a).**

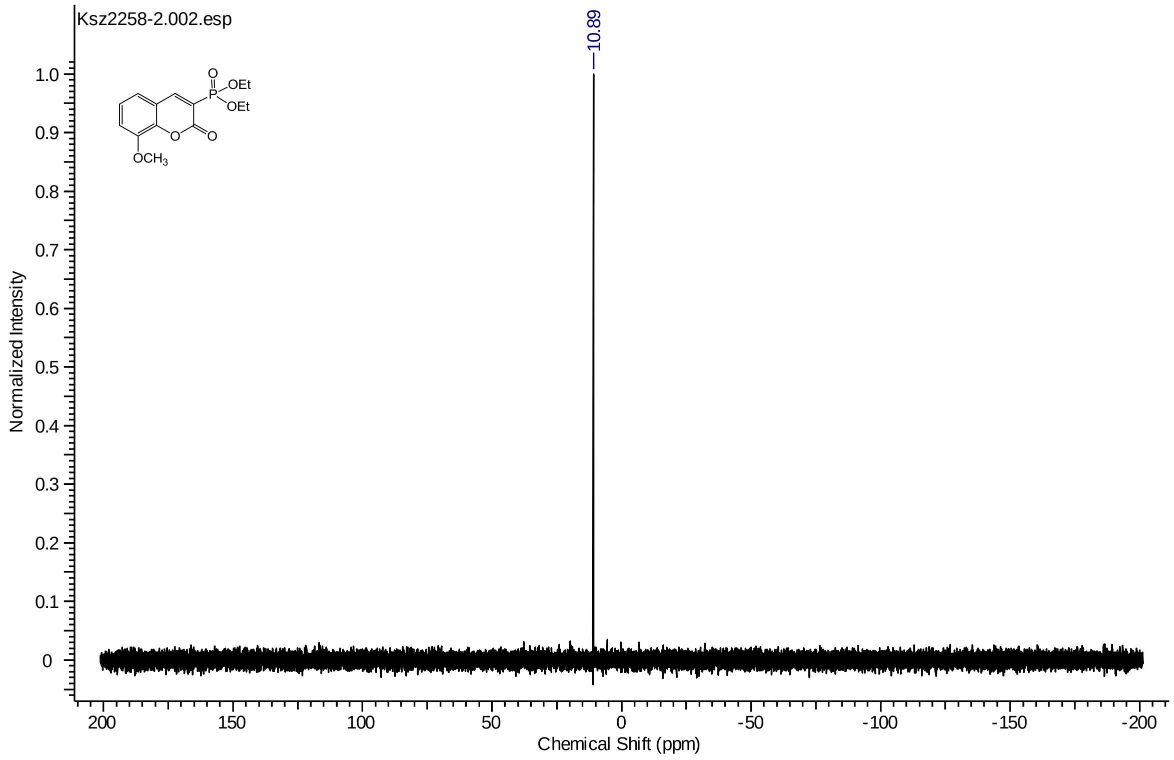

^{31}P NMR of diethyl (8-methoxy-2-oxo-2*H*-chromen-3-yl)phosphonate **(2b).**

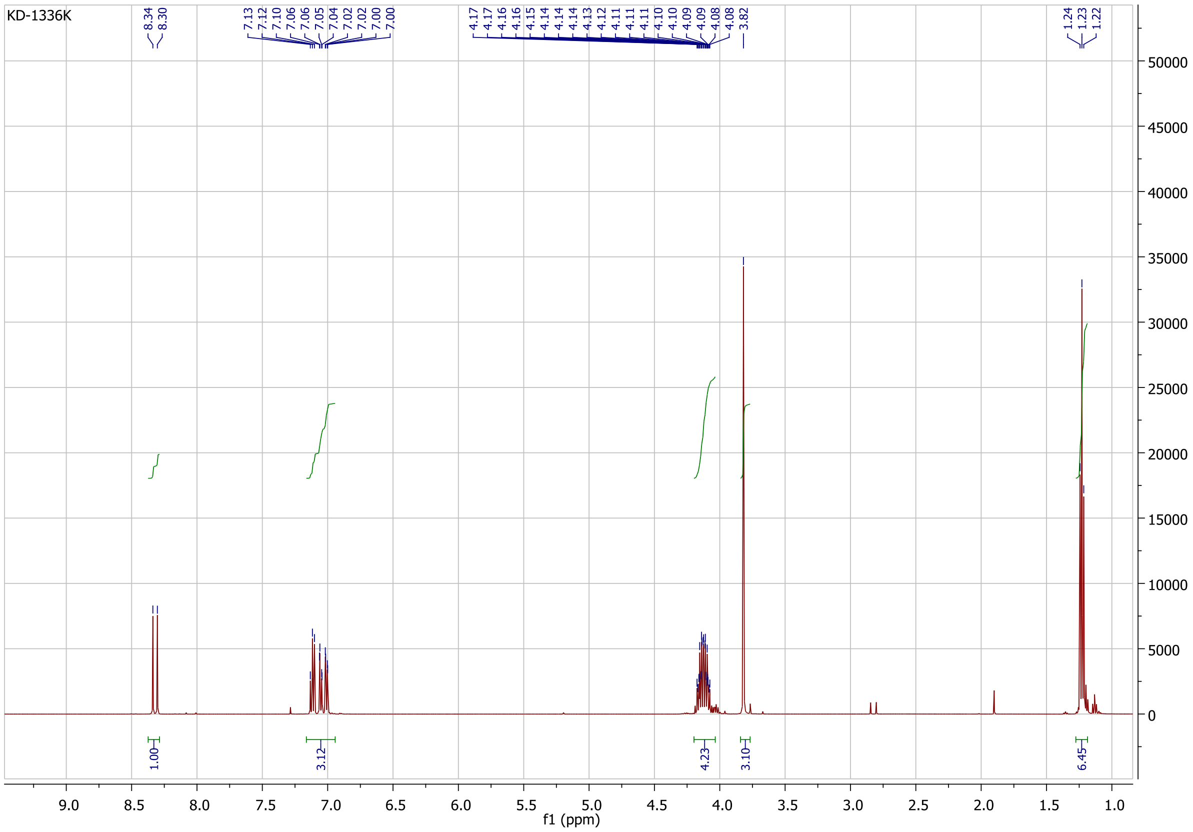

^{1}H NMR of diethyl (8-methoxy-2-oxo-2*H*-chromen-3-yl)phosphonate **(2b).**

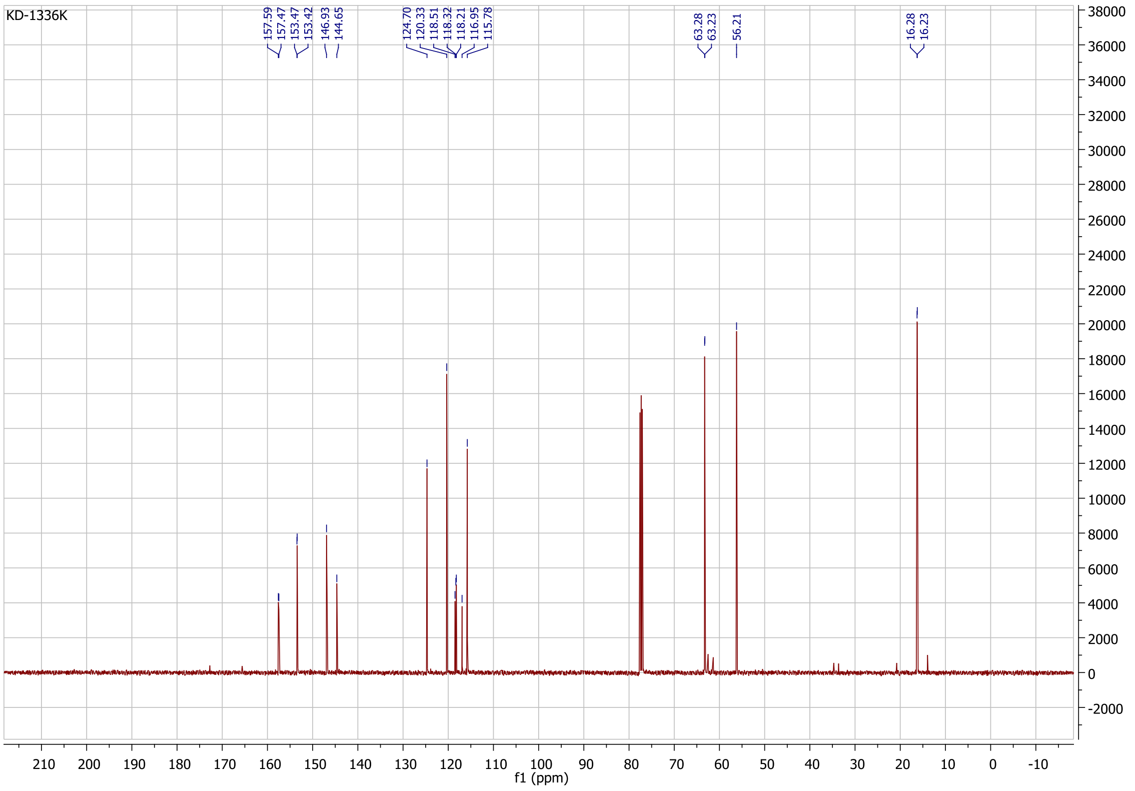

^{13}C NMR of diethyl (8-methoxy-2-oxo-2*H*-chromen-3-yl)phosphonate **(2b).**

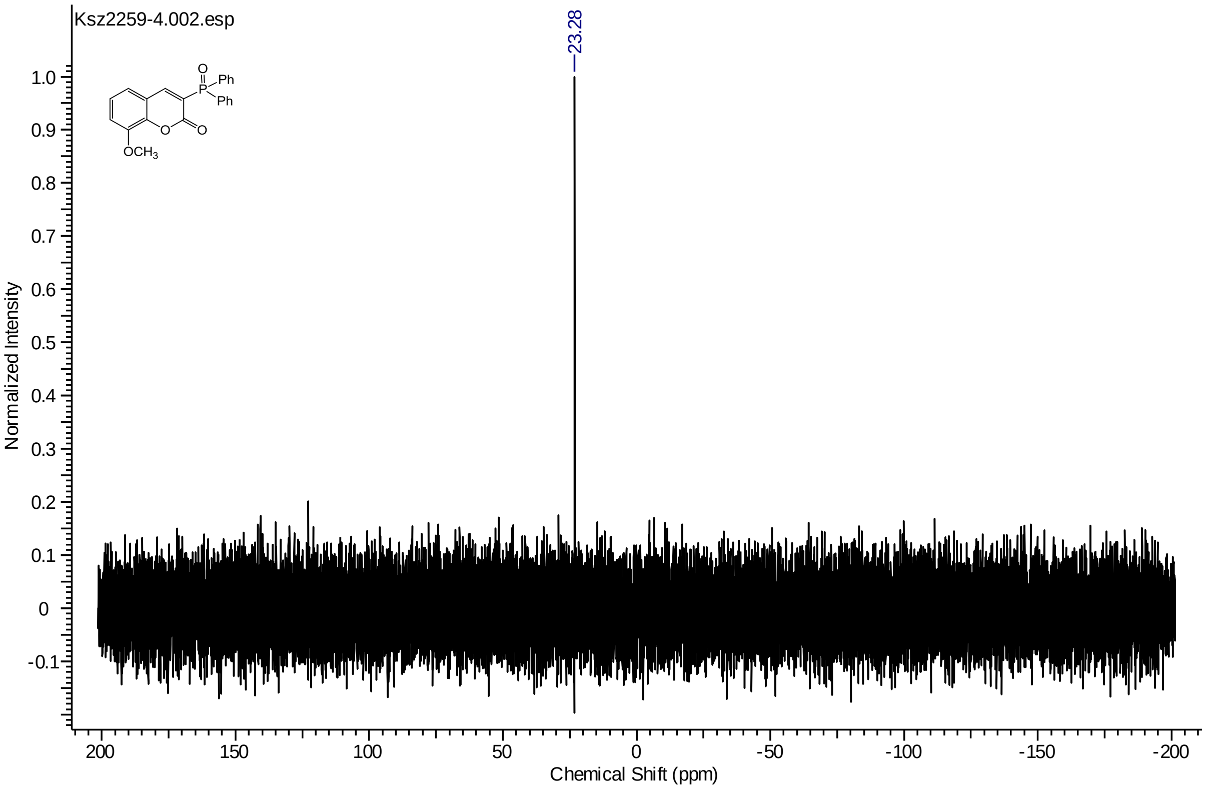

^{31}P NMR of 3-(diphenylphosphinyl)-8-methoxy-2*H*-chromen-2-one **(3).**

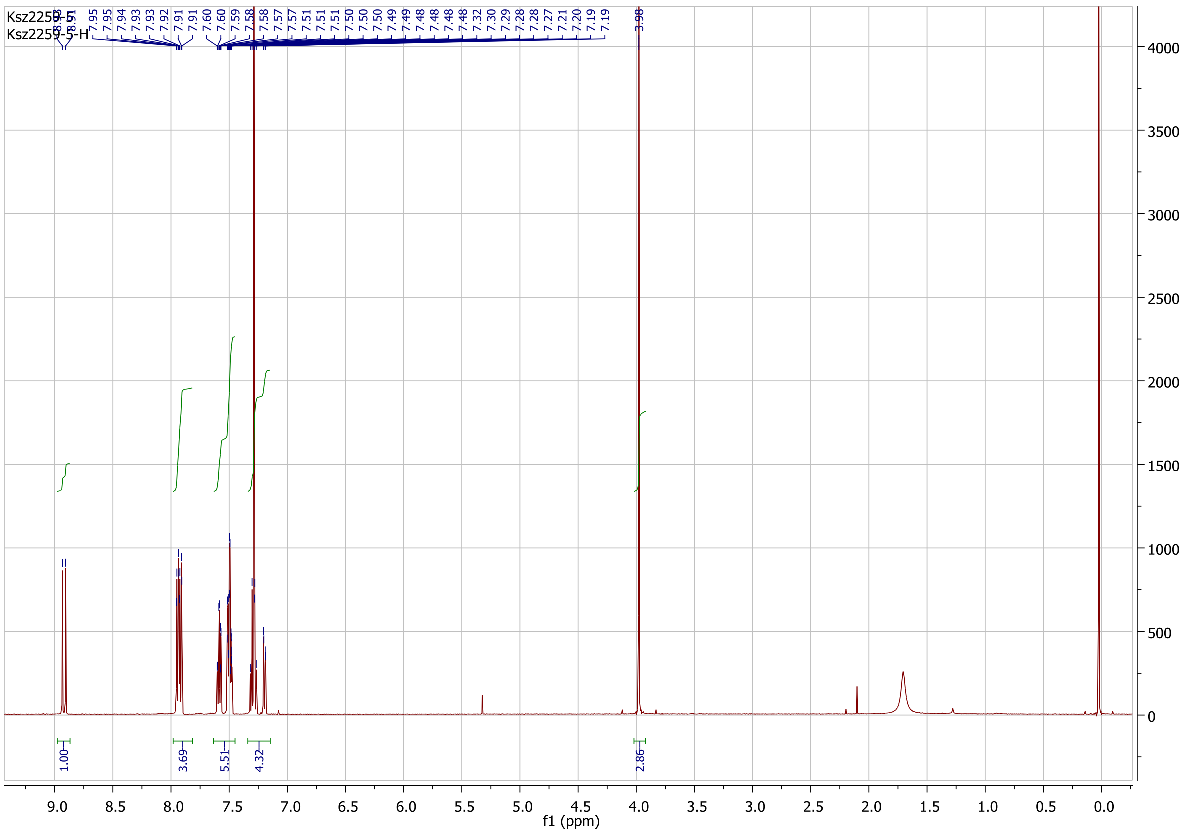

^{1}H NMR of 3-(diphenylphosphinyl)-8-methoxy-2*H*-chromen-2-one **(3).**

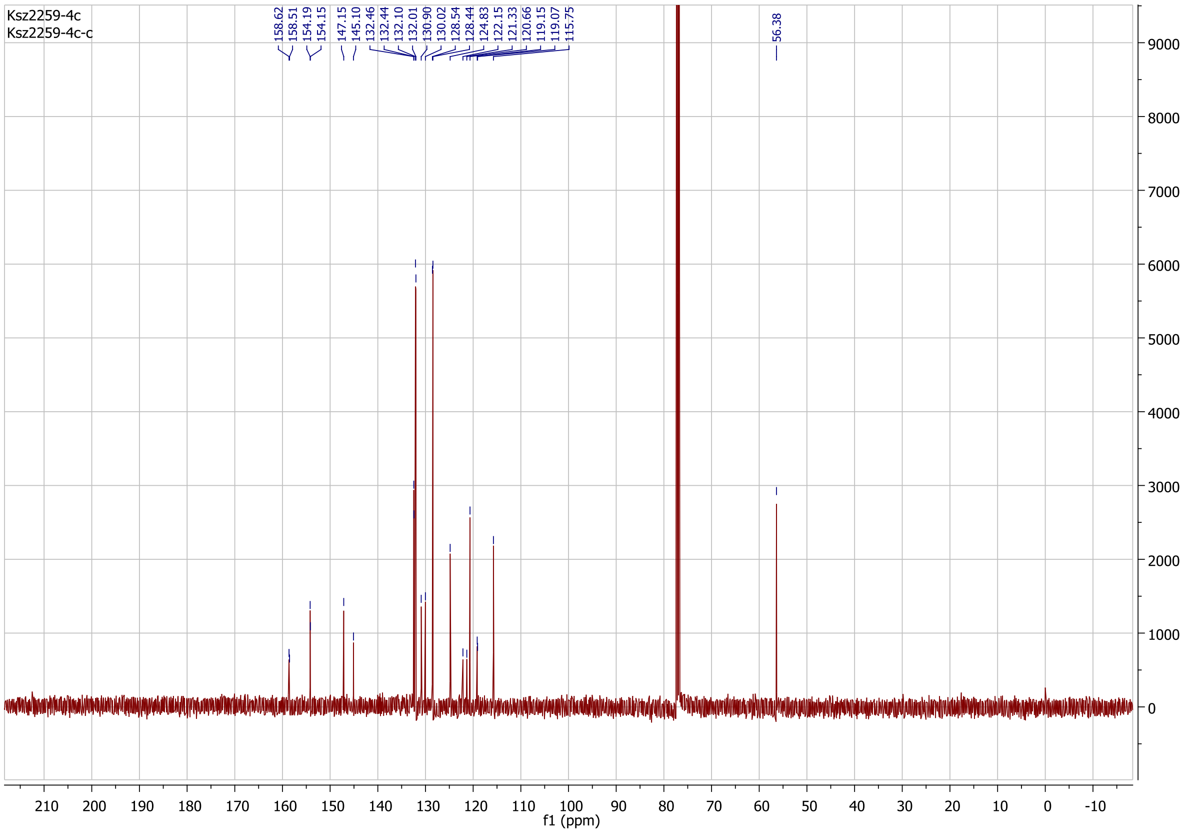

^{13}C NMR of 3-(diphenylphosphinyl)-8-methoxy-2*H*-chromen-2-one **(3).**

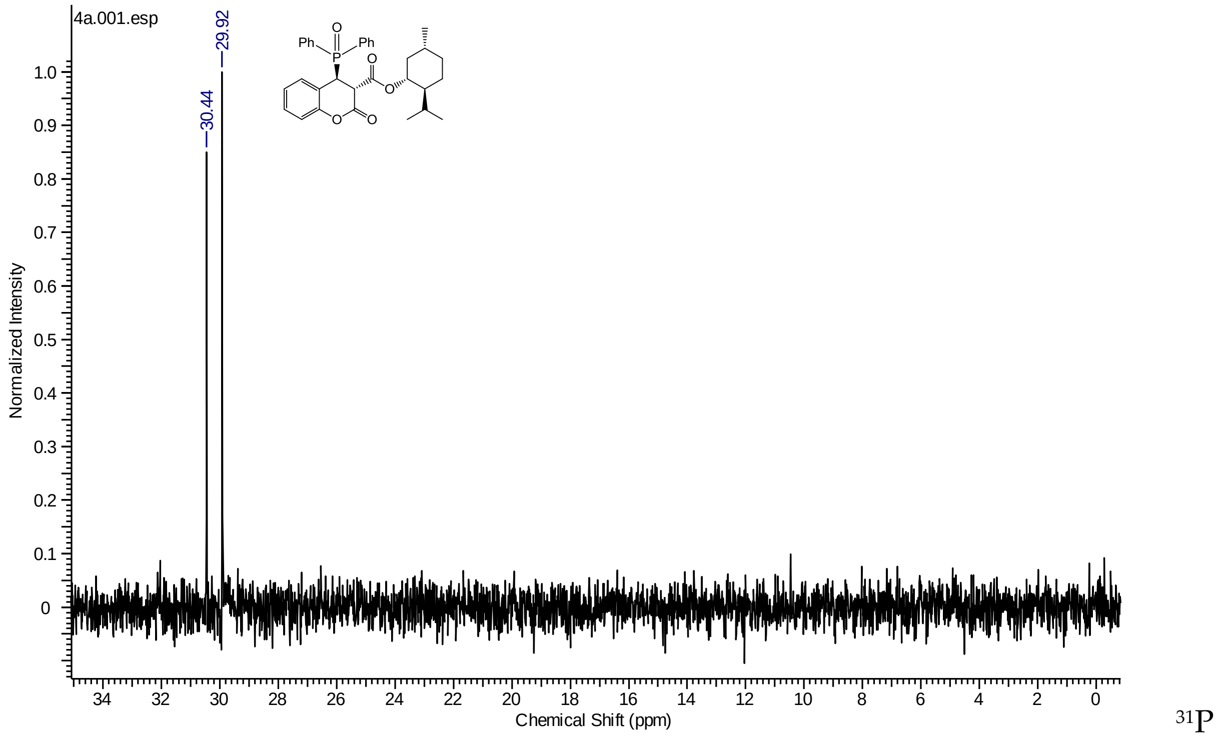

NMR of *trans/cis* mixture of (1*R*,2*S*,5*R*)-2-isopropyl-5-methylcyclohexyl-4-(diphenylphosphoryl)-2-oxochromane-3- carboxylate (**4a**).

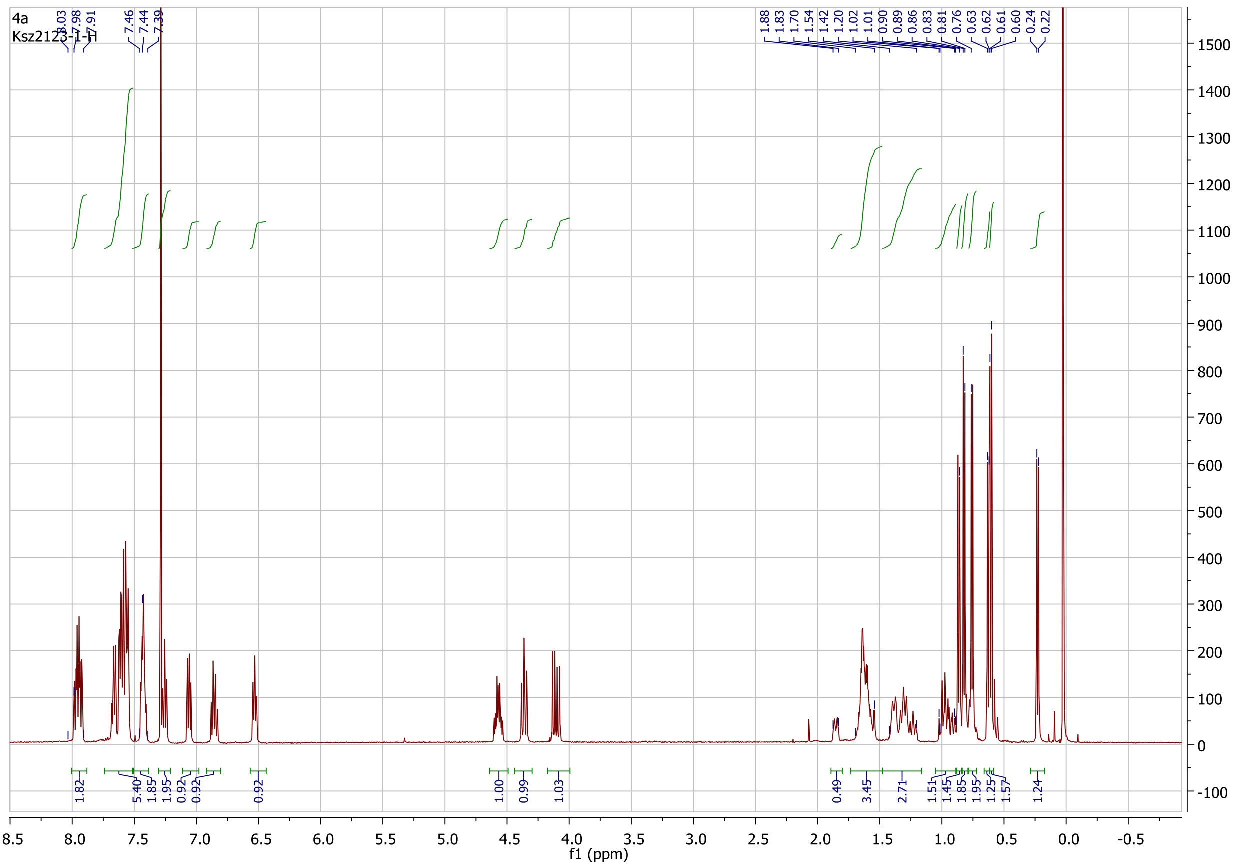

^{1}H NMR of *trans/cis* mixture of (1*R*,2*S*,5*R*)-2-isopropyl-5-methylcyclohexyl-4-(diphenylphosphoryl)-2-oxochromane-3- carboxylate (**4a**).

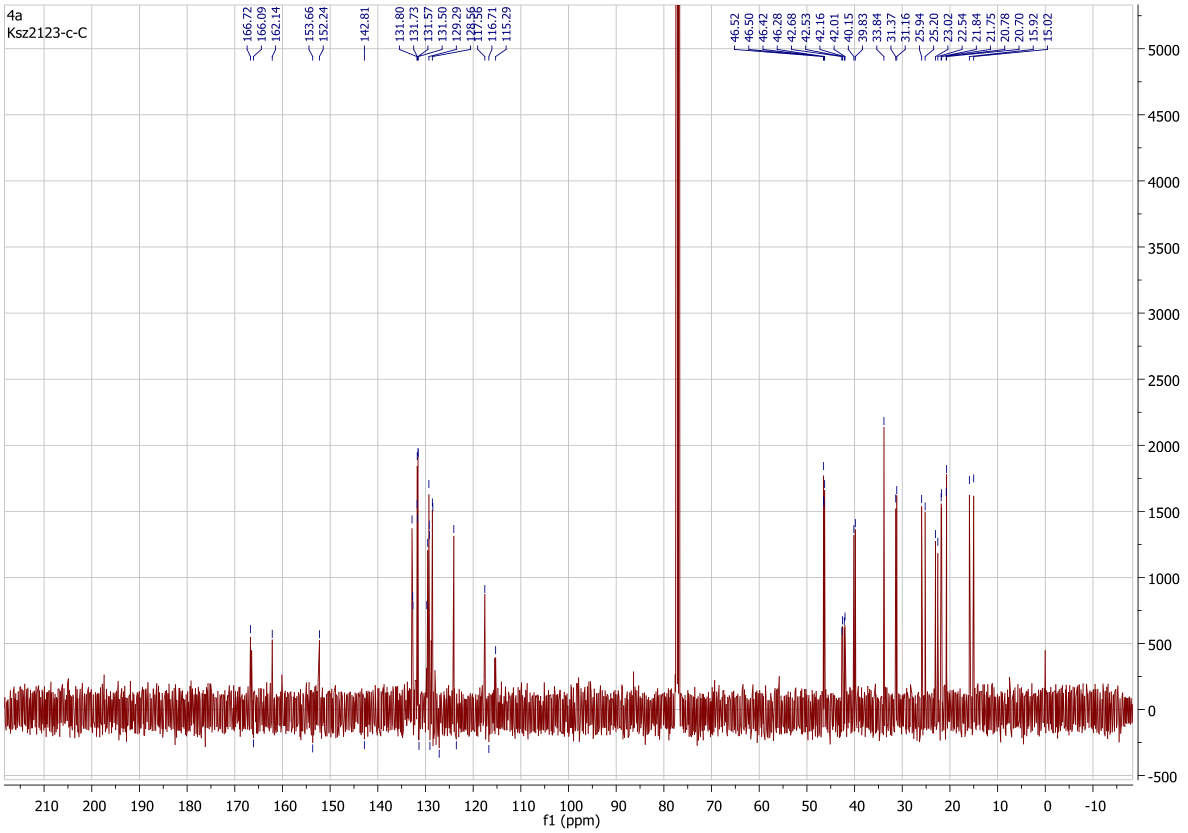

^{13}C NMR of *trans/cis* mixture of (1*R*,2*S*,5*R*)-2-isopropyl-5-methylcyclohexyl-4-(diphenylphosphoryl)-2-oxochromane-3- carboxylate (**4a**).

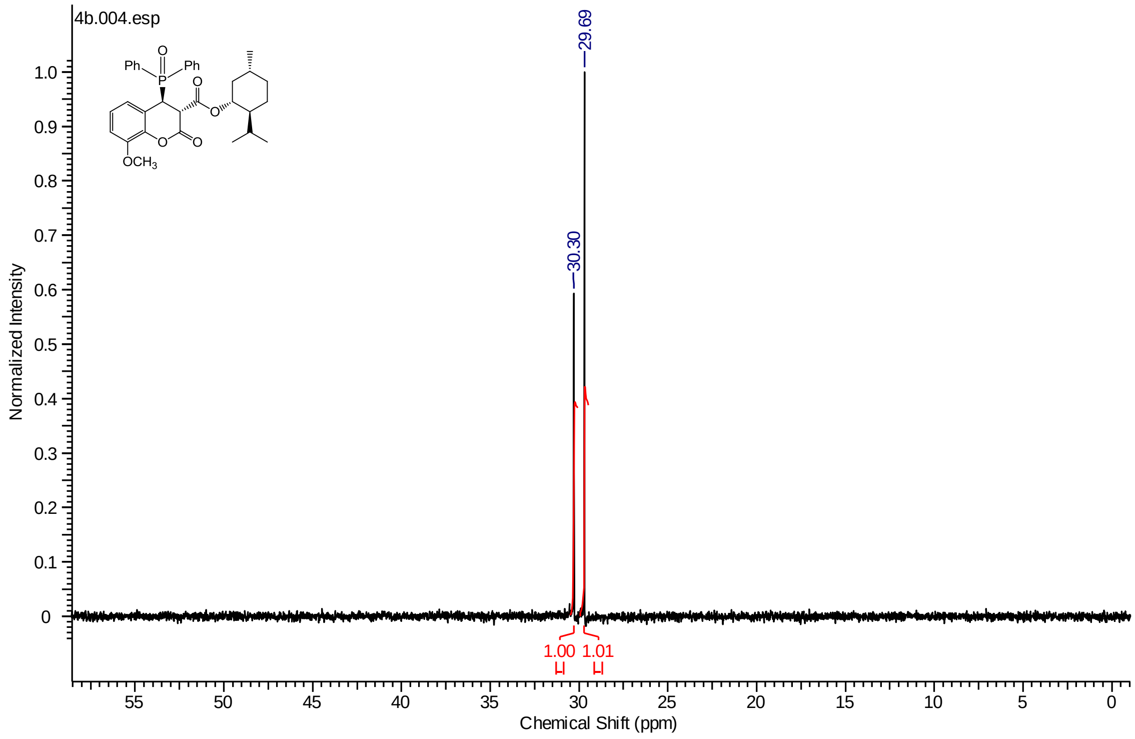

[31] P NMR of *trans/cis* mixture of (1*R*,2*S*,5*R*)-2-isopropyl-5-methylcyclohexyl-4-(diphenylphosphoryl)-8-methoxy-2-oxochromane-3- carboxylate (**4b**).

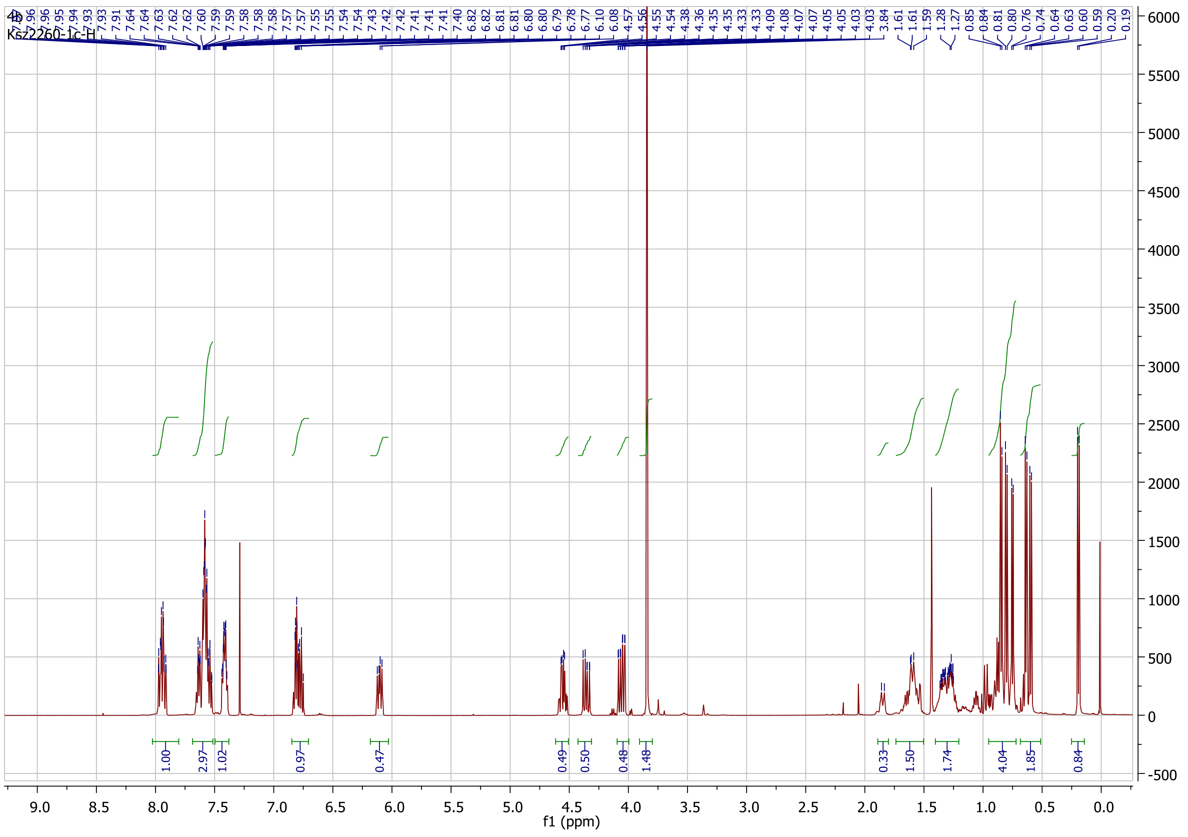

[1] H NMR of *trans/cis* mixture of (1*R*,2*S*,5*R*)-2-isopropyl-5-methylcyclohexyl-4-(diphenylphosphoryl)-8-methoxy-2-oxochromane-3- carboxylate (**4b**).

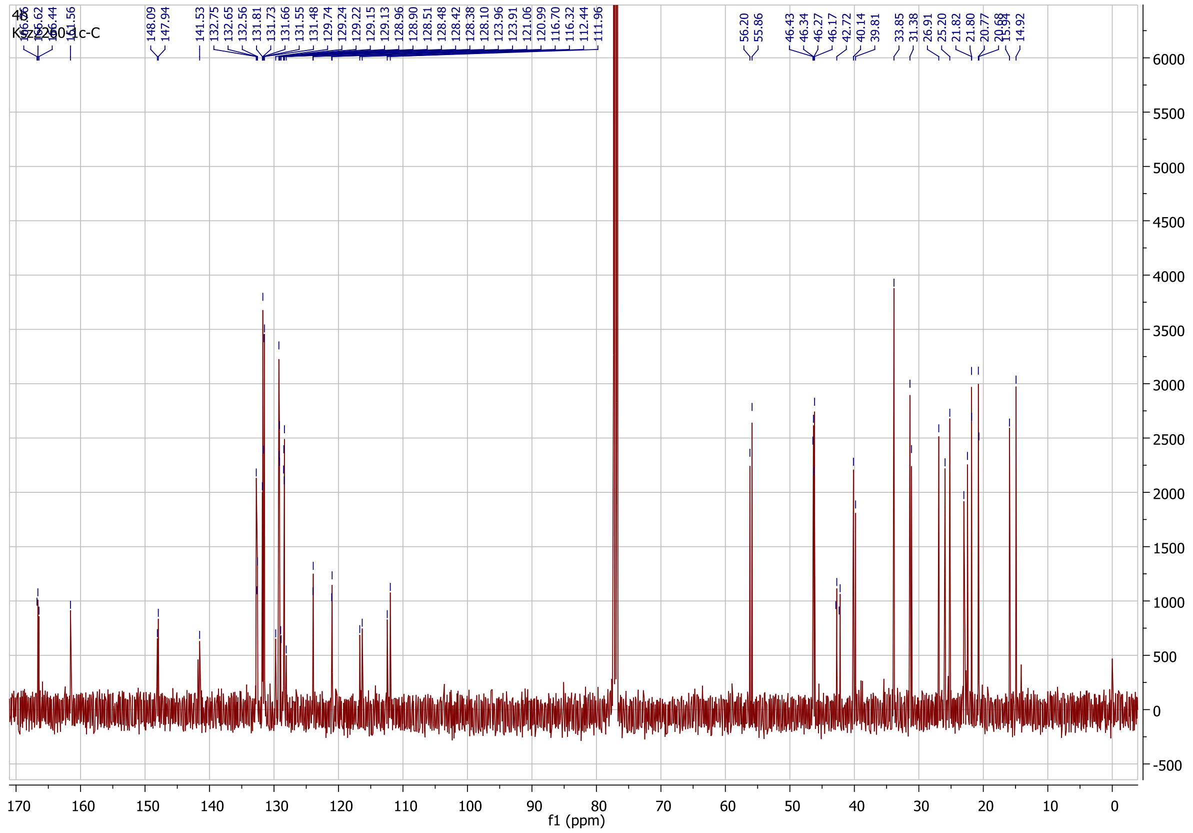

^{13}C NMR of *trans/cis* mixture of (1*R*,2*S*,5*R*)-2-isopropyl-5-methylcyclohexyl-4-(diphenylphosphoryl)-8-methoxy-2-oxochromane-3- carboxylate (**4b**).

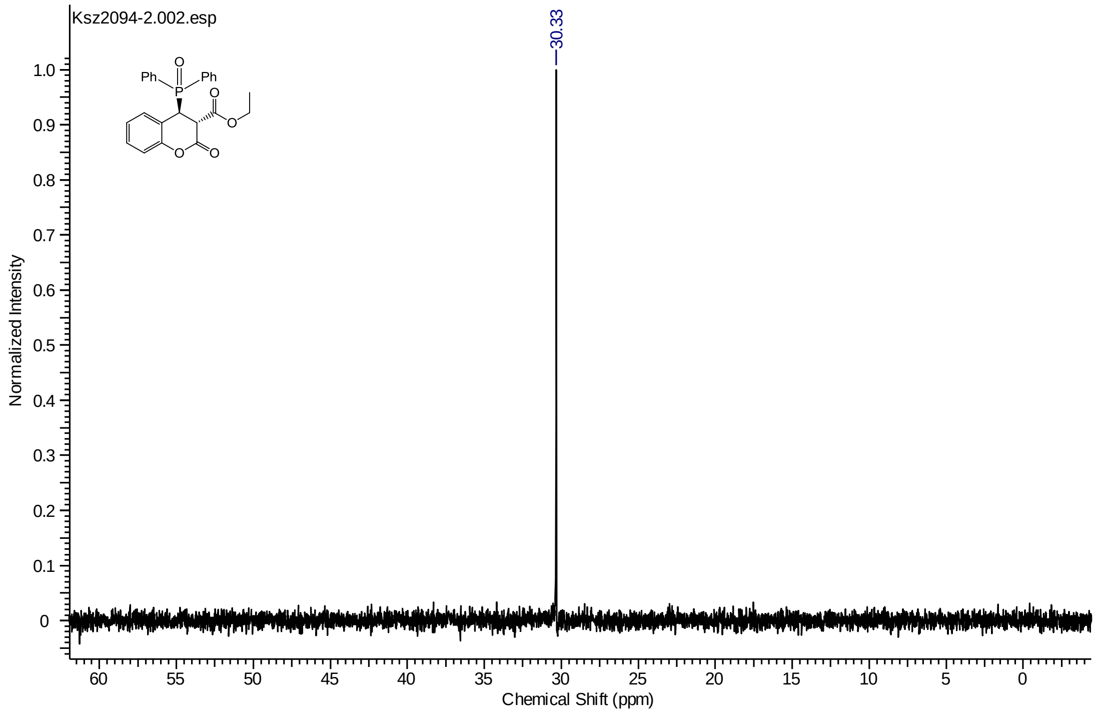

^{31}P NMR of ethyl 4-(diphenylphosphoryl)-2-oxochroman-3-carboxylate (**4c**).

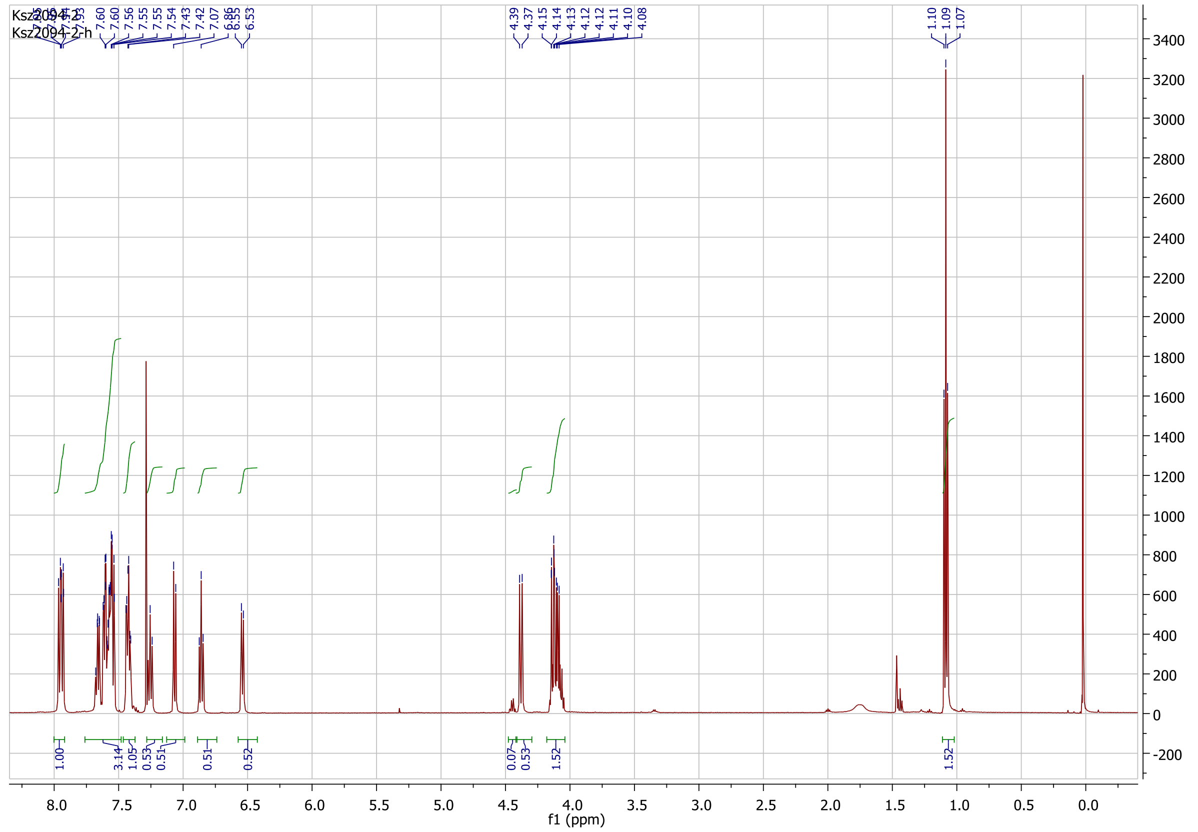

^{1}H NMR of ethyl 4-(diphenylphosphoryl)-2-oxochroman-3-carboxylate (**4c**).

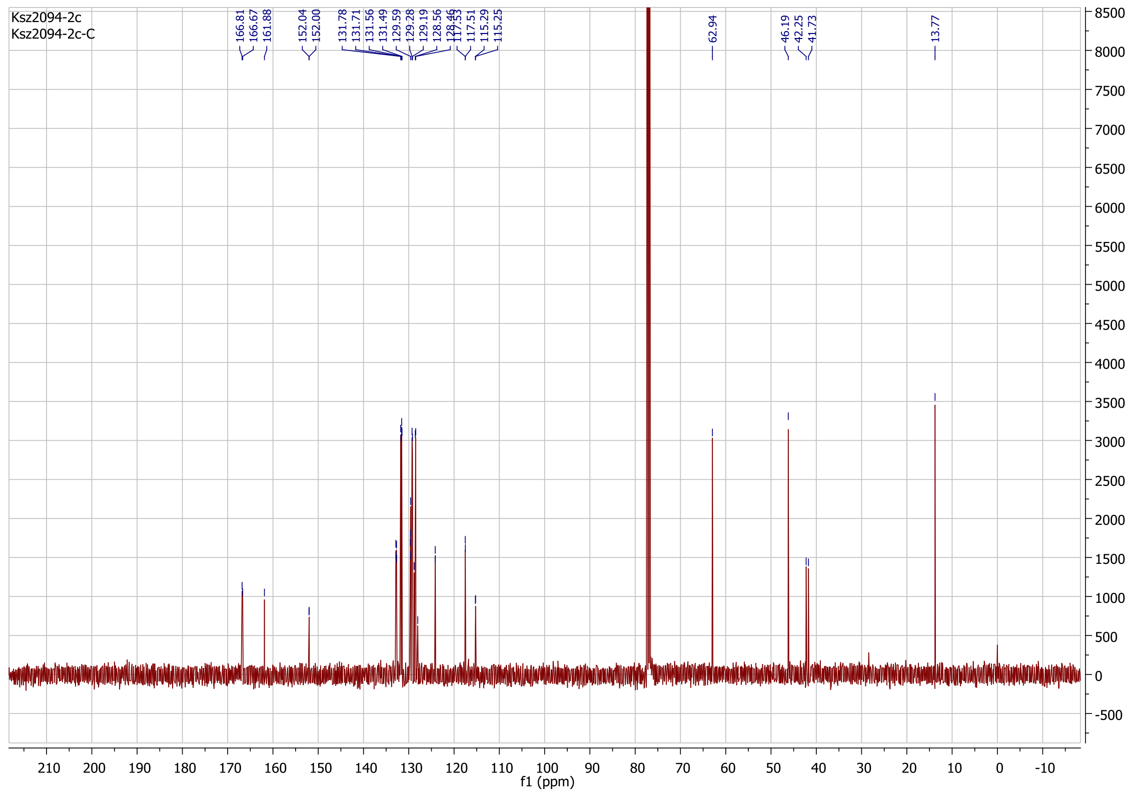

^{13}C NMR of ethyl 4-(diphenylphosphoryl)-2-oxochroman-3-carboxylate (**4c**).

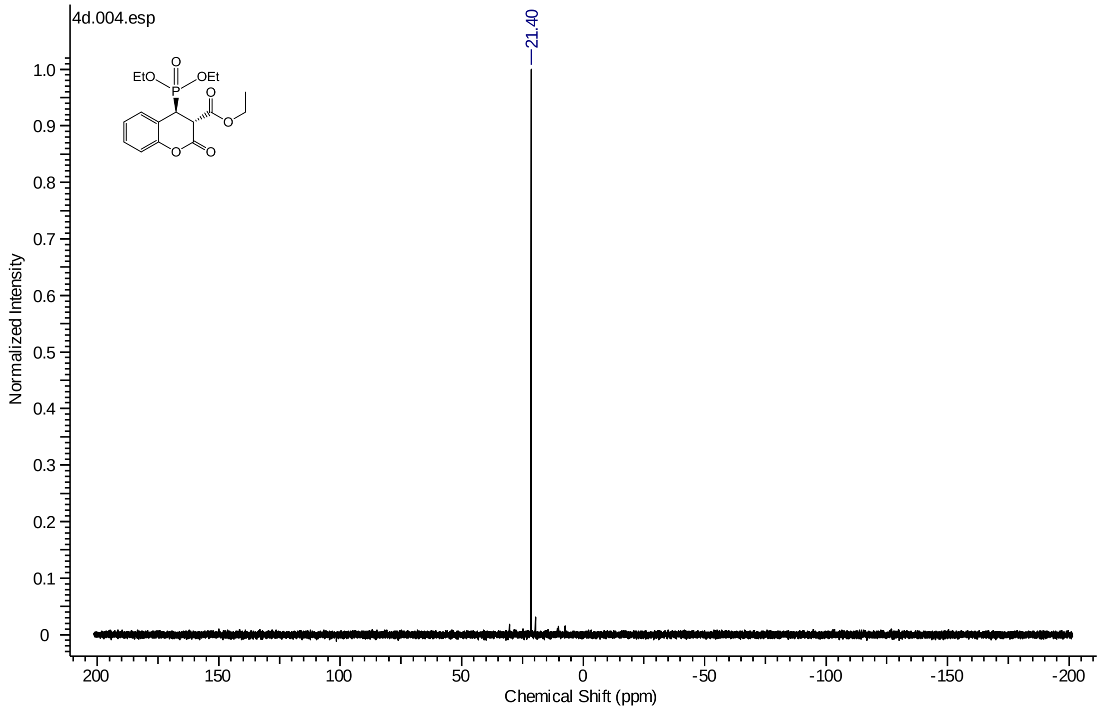

^{31}P NMR of ethyl 4-(diethoxyphosphoryl)-2-oxochroman-3-carboxylate (**4d**).

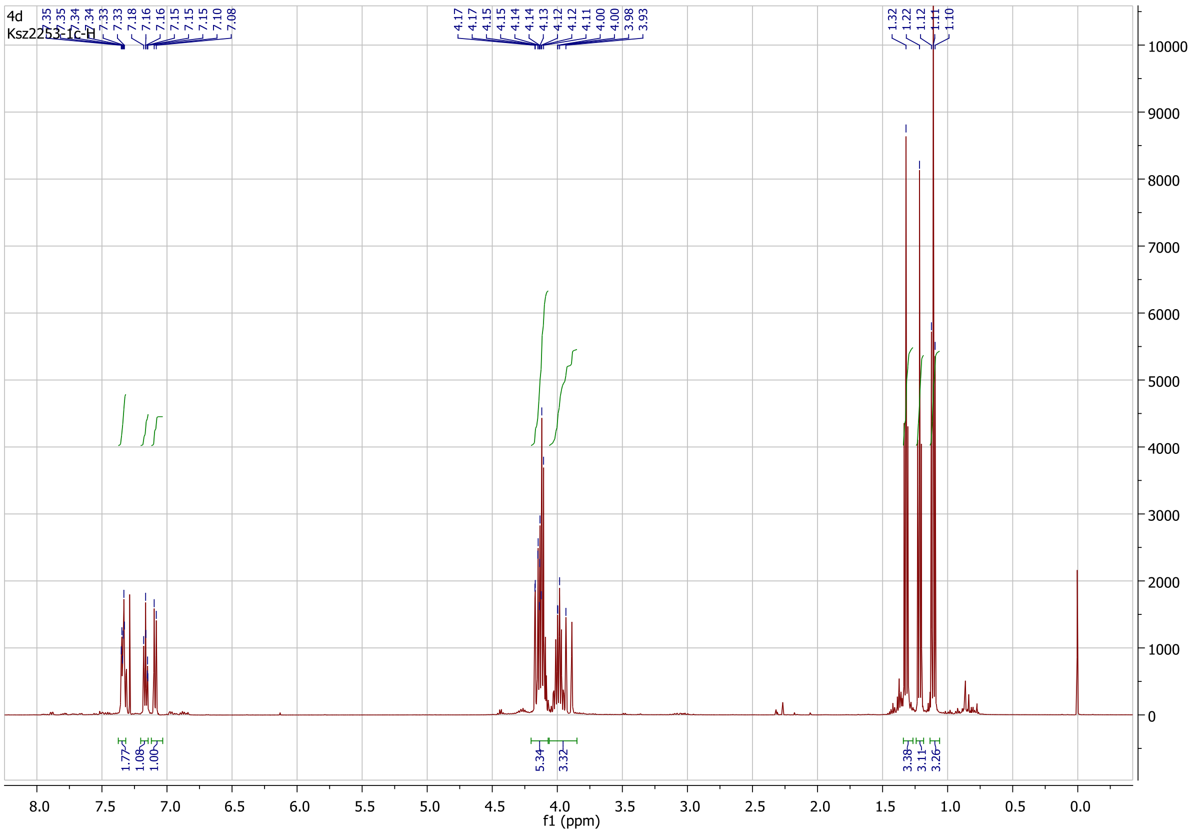

^{1}H NMR of ethyl 4-(diethoxyphosphoryl)-2-oxochroman-3-carboxylate (**4d**).

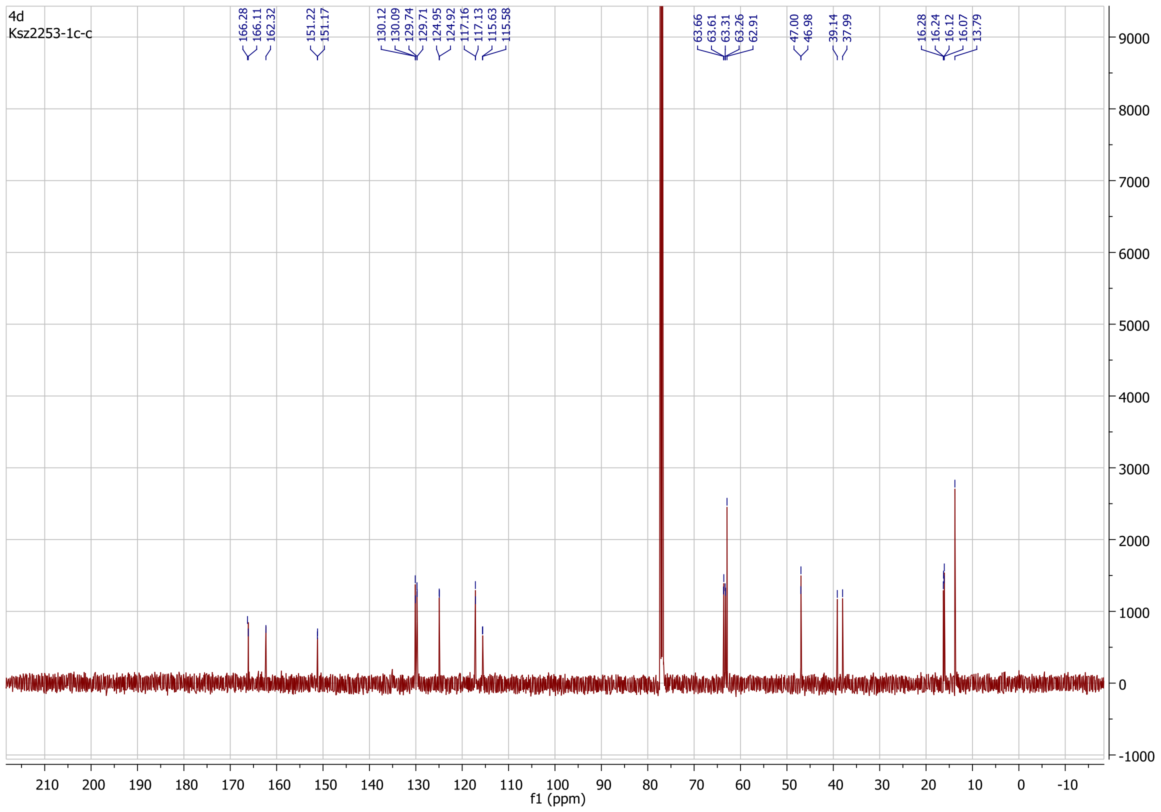

^{13}C NMR of ethyl 4-(diethoxyphosphoryl)-2-oxochroman-3-carboxylate (**4d**).

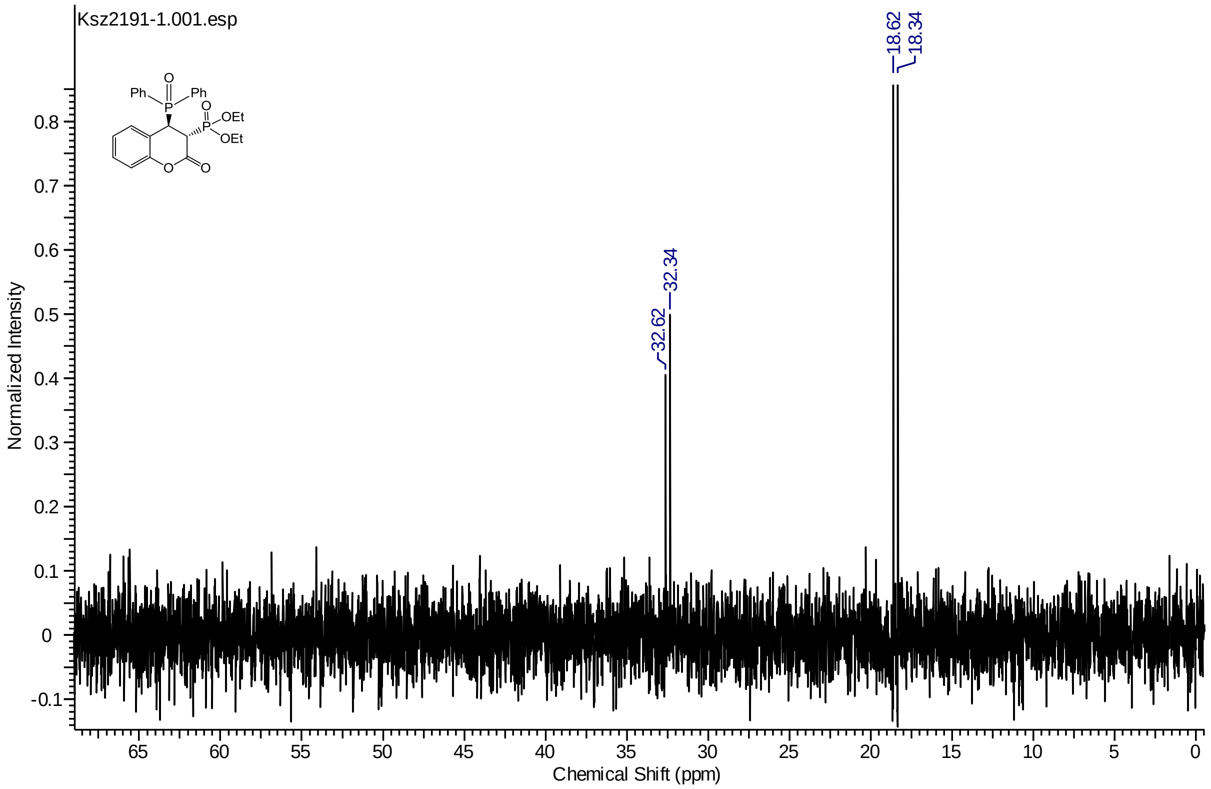

^{31}P NMR of diethyl-4-(diphenylphosphoryl)-2-oxochroman-3-yl)phosphonate (**5a**).

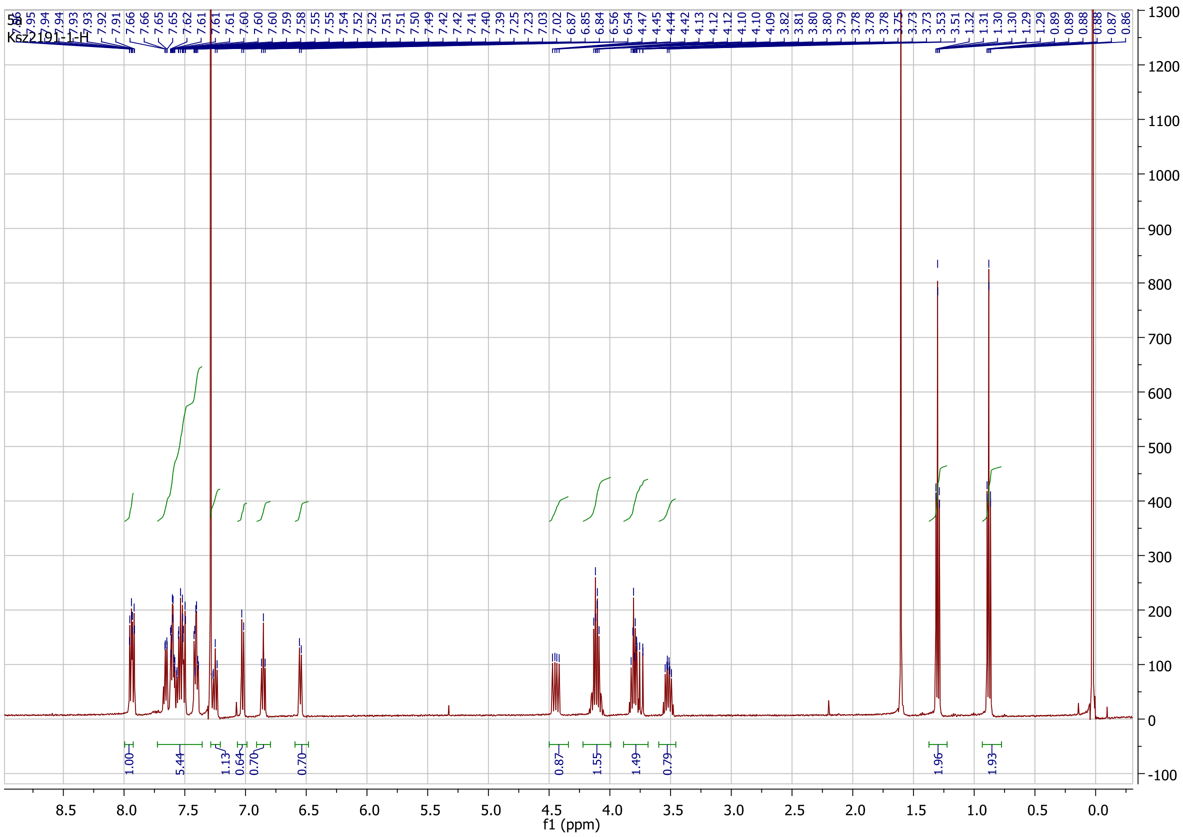

^{1}H NMR of diethyl-4-(diphenylphosphoryl)-2-oxochroman-3-yl)phosphonate (**5a**).

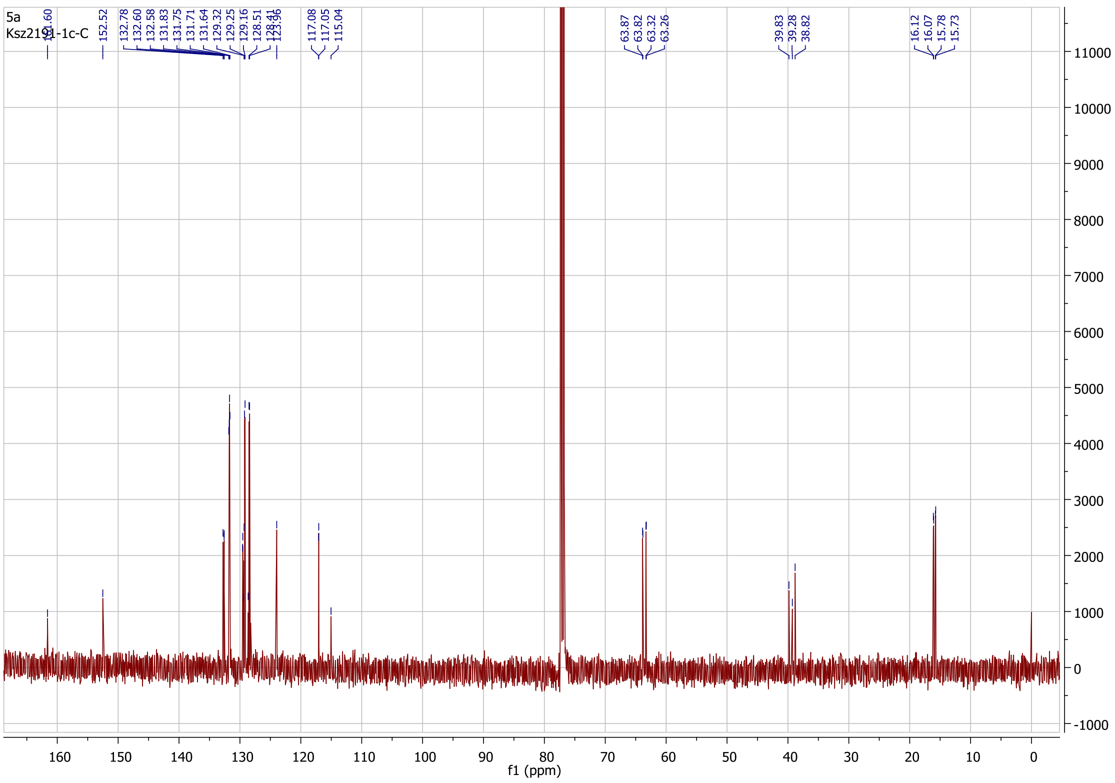

^{13}C NMR of diethyl-4-(diphenylphosphoryl)-2-oxochroman-3-yl)phosphonate (**5a**).

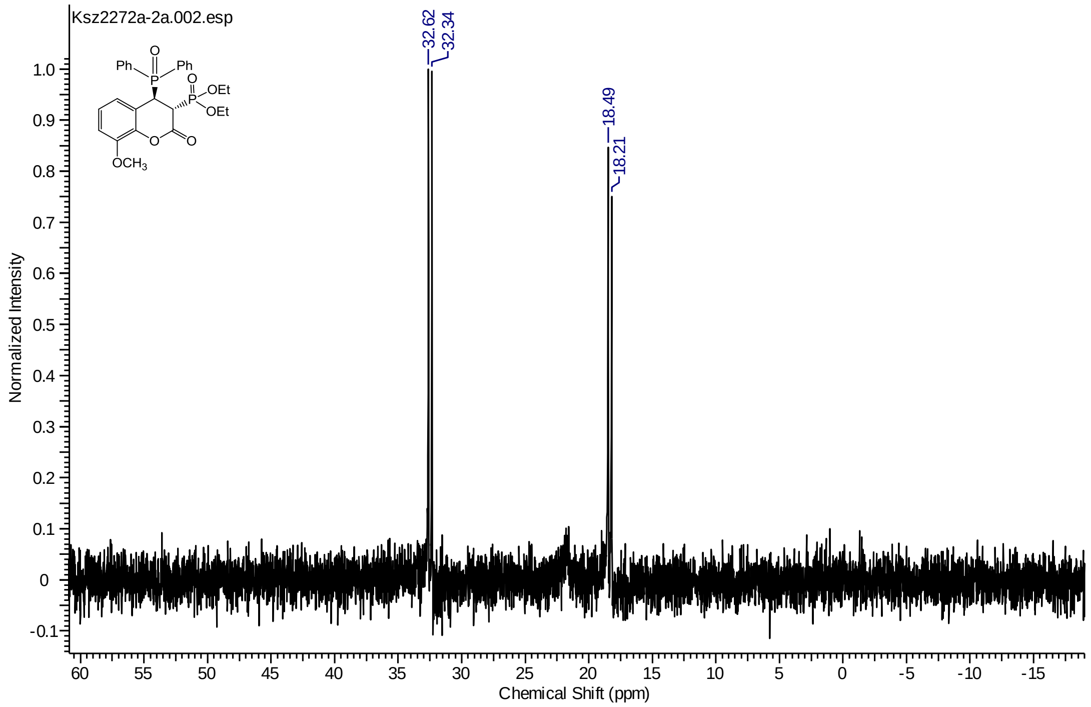

^{31}P NMR of diethyl (4-(diphenylphosphoryl)-8-methoxy-2-oxochroman-3-yl)phosphonate (**5b**)

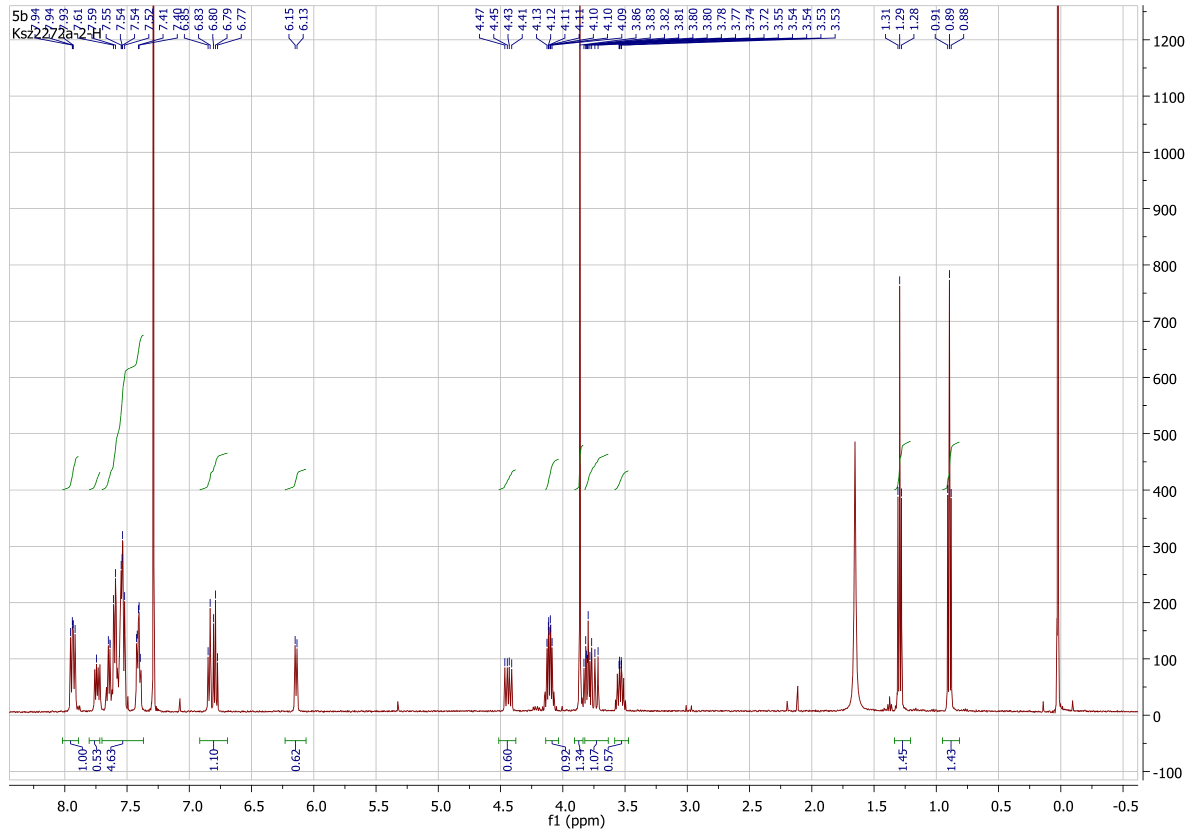

^{1}H NMR of diethyl (4-(diphenylphosphoryl)-8-methoxy-2-oxochroman-3-yl)phosphonate (**5b**).

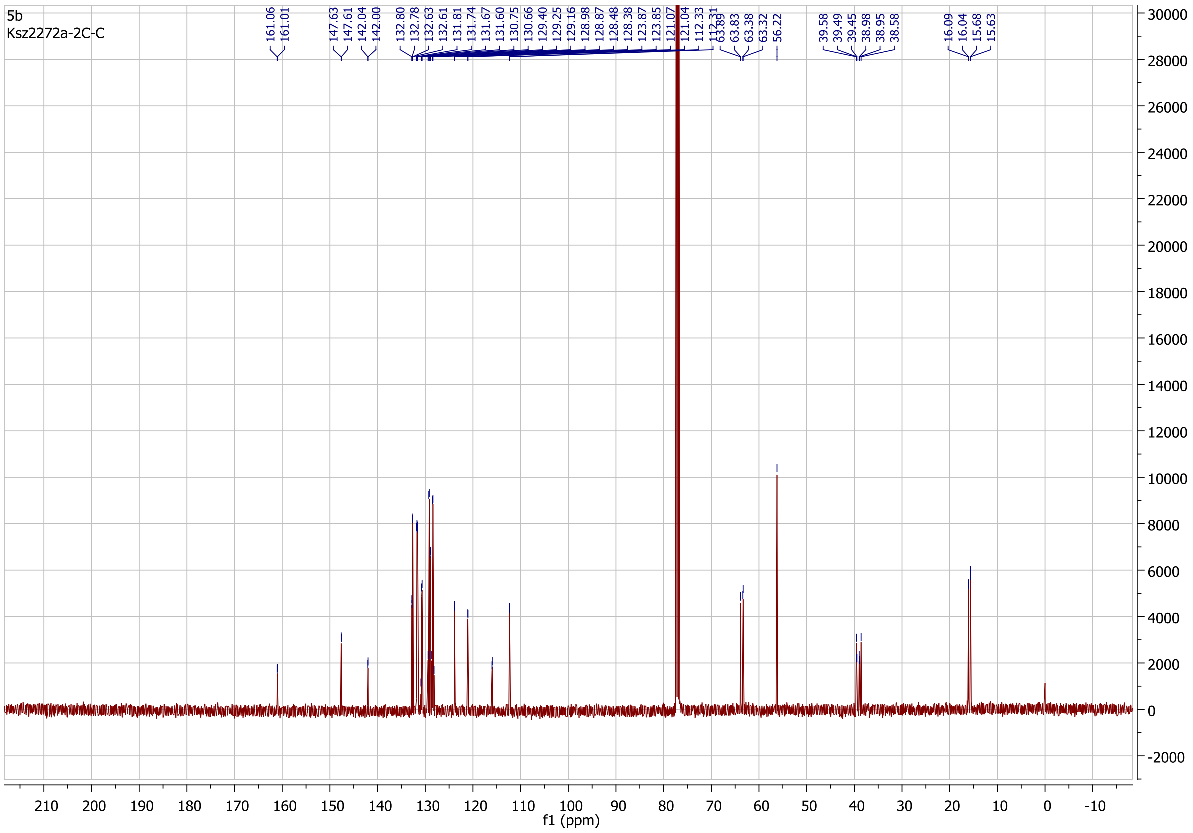

^{13}C NMR of diethyl (4-(diphenylphosphoryl)-8-methoxy-2-oxochroman-3-yl)phosphonate (**5b**).

6. HRMS Spectra of Compounds 1c–1e, 4a–4c, and 5a–5b

No.	Molecular formula	Theoretical mass [M+H]+ (Da)	Experimental mass [M+H]+ (Da)	Δ mDa	Δ ppm
1C	$C_{21}H_{27}O_5$	359.18585	359.18594	0.09	0.25

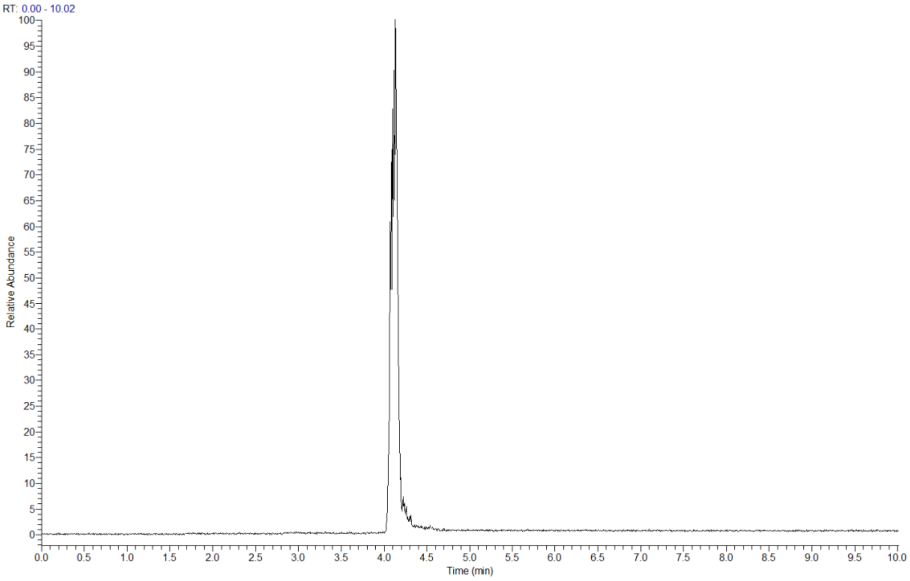

No.	Molecular formula	Theoretical mass [M+H]+ (Da)	Experimental mass [M+H]+ (Da)	Δ mDa	Δ ppm
1D	$C_{21}H_{27}O_5$	359.18585	359.18575	-0.10	0.28

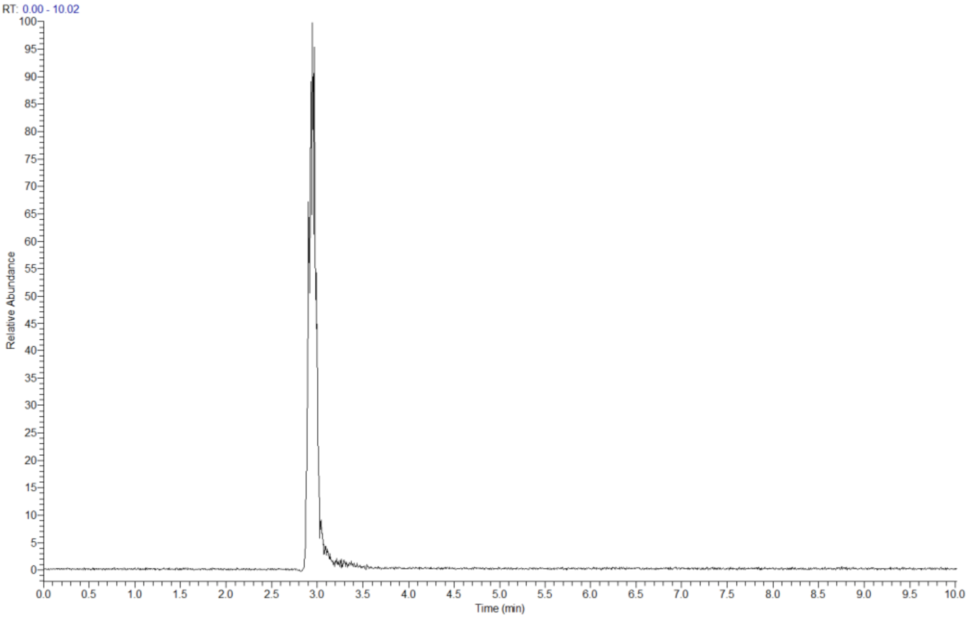

No.	Molecular formula	Theoretical mass [M+H]+ (Da)	Experimental mass [M+H]+ (Da)	Δ mDa	Δ ppm
1E	$C_{21}H_{27}O_5$	359.18585	359.18575	-0.10	0.28

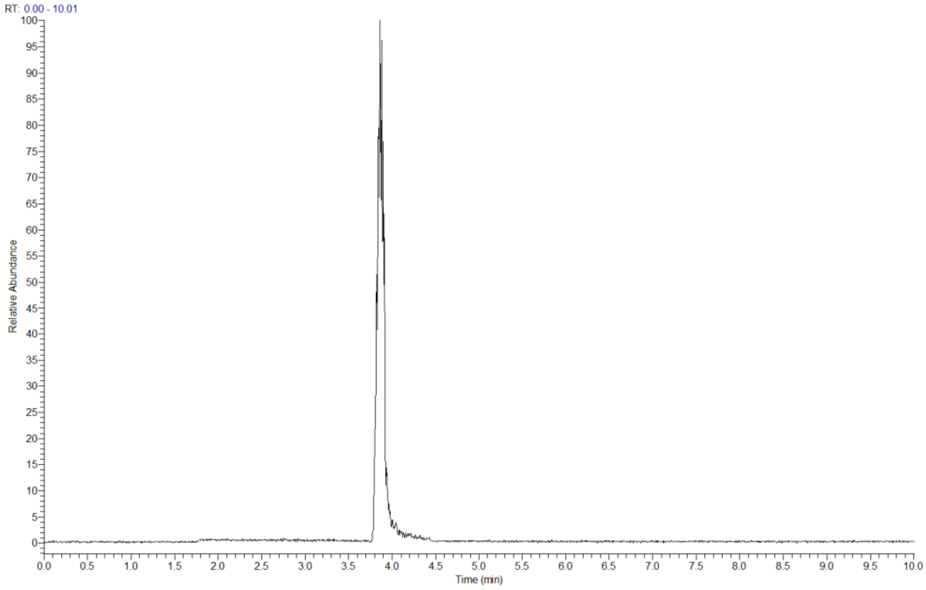

No.	Molecular formula	Theoretical mass [M+H]+ (Da)	Experimental mass [M+H]+ (Da)	Δ mDa	Δ ppm
4A	$C_{32}H_{36}O_5P$	531.23004	531.23014	0.10	0.19

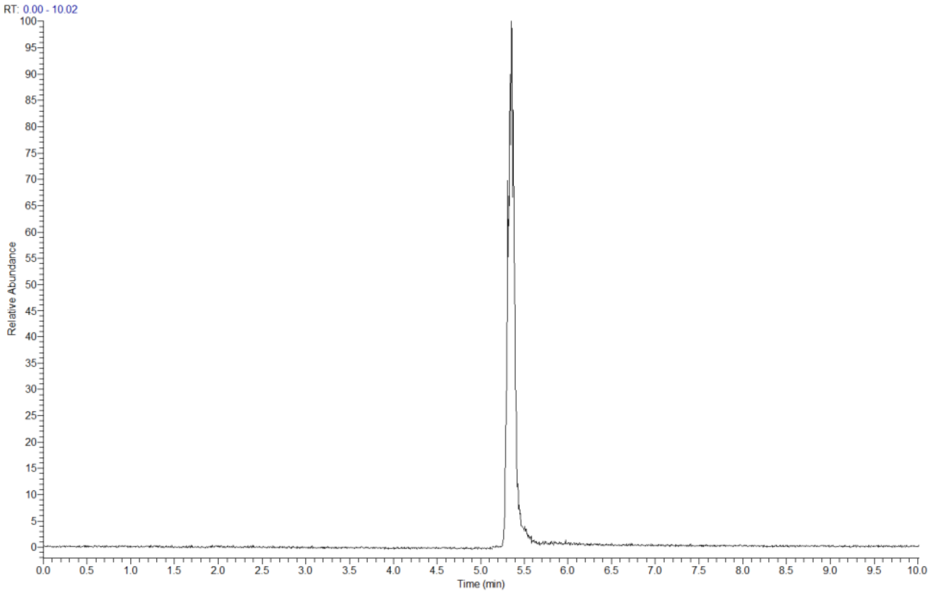

No.	Molecular formula	Theoretical mass [M+H]+ (Da)	Experimental mass [M+H]+ (Da)	Δ mDa	Δ ppm
**4B**	**$C_{33}H_{38}O_6P$**	**561.24060**	**561.24067**	**0.07**	**0.12**

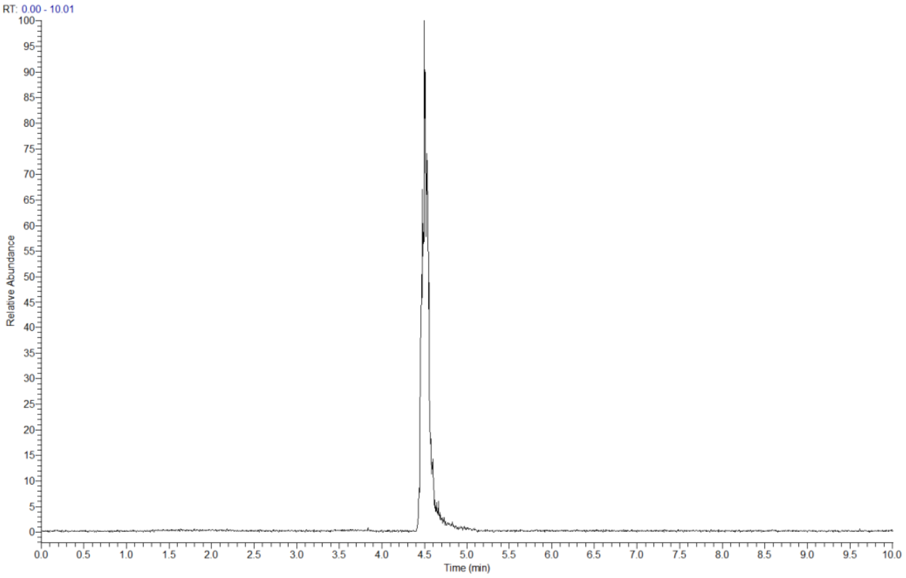

No.	Molecular formula	Theoretical mass [M+H]+ (Da)	Experimental mass [M+H]+ (Da)	Δ mDa	Δ ppm
**4C**	**$C_{24}H_{22}O_5P$**	**421.12049**	**421.12053**	**0.04**	**0.09**

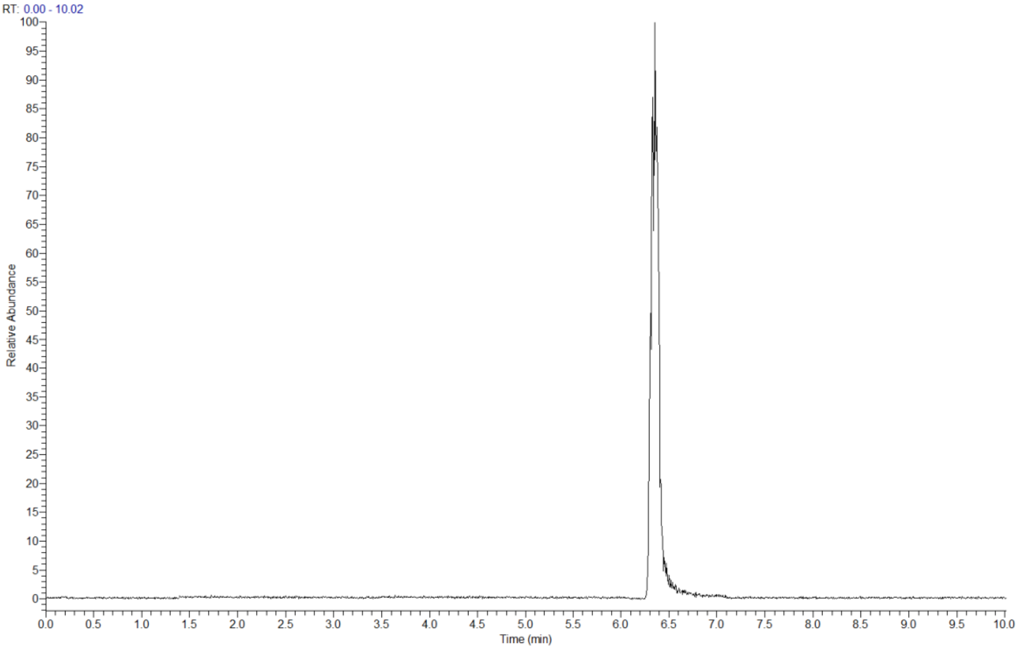

No.	Molecular formula	Theoretical mass [M+H]+ (Da)	Experimental mass [M+H]+ (Da)	Δ mDa	Δ ppm
5A	$C_{25}H_{27}O_6P_2$	485.12829	485.12819	-0.10	0.21

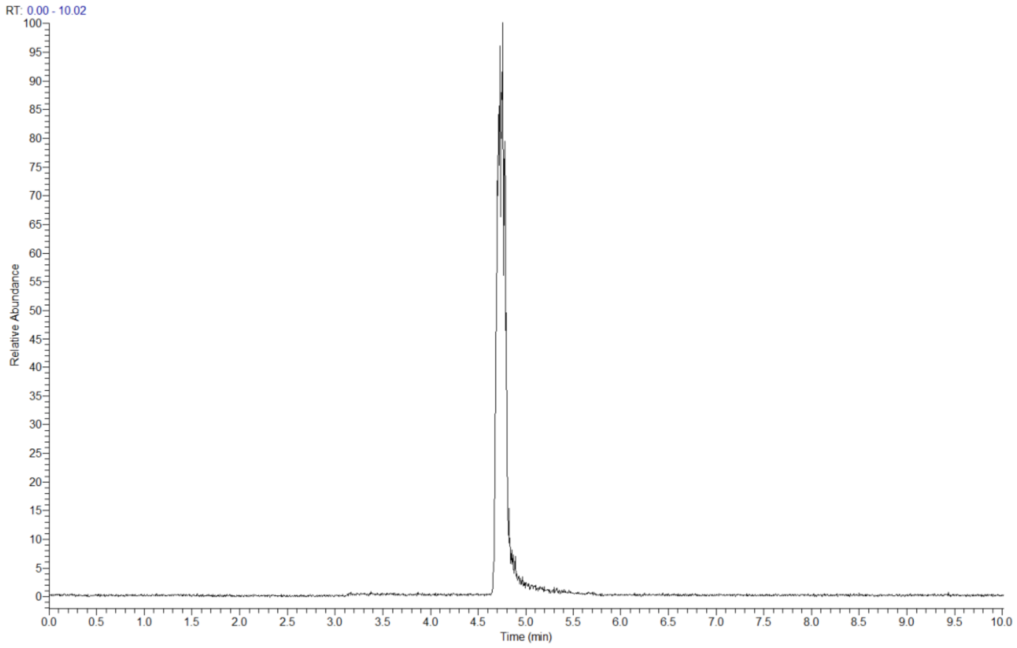

No.	Molecular formula	Theoretical mass [M+H]+ (Da)	Experimental mass [M+H]+ (Da)	Δ mDa	Δ ppm
5B	$C_{26}H_{29}O_7P_2$	515.13886	515.13873	-0.13	0.25

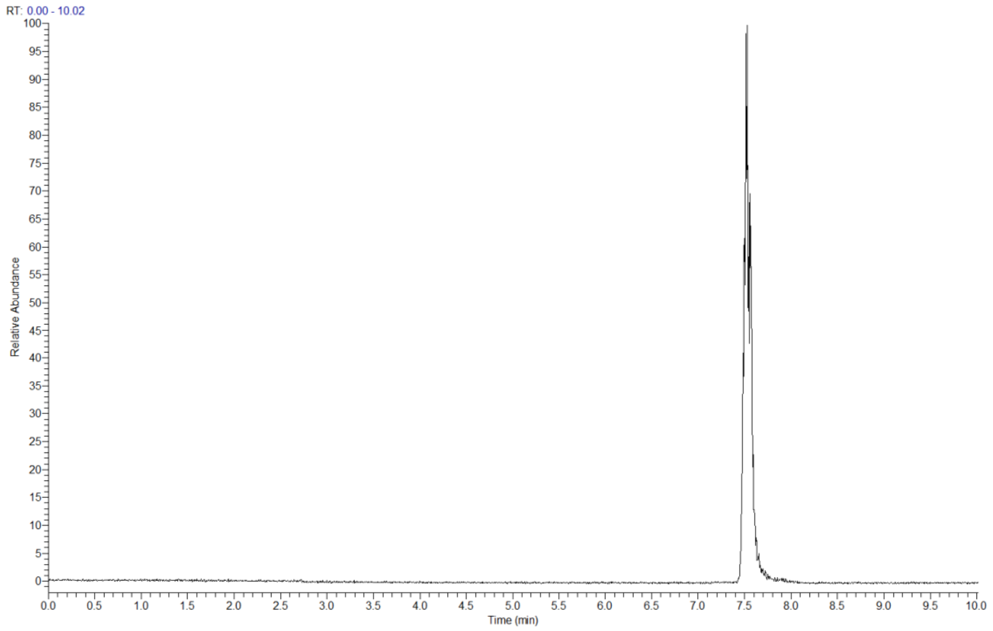

7. References

[1] Agilent Technologies Inc. (2014) Yarnton, UK.

[2] CrysAlisPro 1.171.42.79a (2022) Rigaku Oxford Diffraction, Tokio, Japan.

[3] G.M. Sheldrick, Acta Cryst. 2015, A71, 3–8
